# Lyophilized Synthetic Platelets: In Vitro Characterization and In Vivo Evaluation in Mouse Thrombocytopenia Model

**DOI:** 10.1002/advs.202600002

**Published:** 2026-04-20

**Authors:** Ujjal Didar Singh Sekhon, Dante Disharoon, Shrijal S. Desai, Baylee Traylor, Emily Gahagan, Emma Quill, Kristin Aldridge, Norman F. Luc, Sonali Rohiwal, Shruti Raghunathan, Rebecca Ahn, Sana Syed, Alexander Dornback, Bipin Chakravarthy Paruchuri, Andrew Ditto, Susan M. Shea, Philip C. Spinella, Matthew D. Neal, Michael A. Bruckman, Christa L. Pawlowski, Anirban Sen Gupta

**Affiliations:** ^1^ Haima Therapeutics LLC Cleveland OH USA; ^2^ Case Western Reserve University Department of Biomedical Engineering Cleveland OH USA; ^3^ University of Pittsburgh Trauma and Transfusion Medicine Research Center Department of Surgery Pittsburgh PA USA; ^4^ University of Pittsburgh Department of Bioengineering Pittsburgh PA USA

**Keywords:** hemostasis, platelets, lyophilized synthetic platelet, platelet transfusion, hemorrhage

## Abstract

Platelet transfusions to treat bleeding complications use donor‐derived platelets stored at room‐temperature, that have a shelf‐life of only 5–7 days due to bacterial contamination risks. Cold‐stored and freeze‐dried platelets are being investigated for extending shelf‐life, but these still have the inherent challenge of donor‐dependency. To address this, we developed a liposome‐based synthetic platelet (SP) nanoconstruct that mimics the primary hemostatic mechanisms of platelets, and have previously reported its efficacy. However, a logistical limitation of SP is its susceptibility to environment‐induced physico‐chemical instabilities that reduces shelf‐life when stored as aqueous suspension, impacting its availability and bioactivity. Lyophilization of liposomal nanotherapeutics utilizing anhydrobiosis‐inspired lyoprotectants can enhance shelf‐life, but this has not been explored for synthetic platelets. With these considerations, we report advancing SP into a lyophilized product (Lyo‐SP) that can be rapidly aqueous‐reconstituted for on‐demand use. Lyo‐SP retained morphology, size, and charge in long‐term storage at various temperatures, and conserved platelet‐mimetic functions in multiparametric assays with human plasma and blood. Lyo‐SP demonstrated biosafety in its effect analysis on endothelial cells, neutrophils, RBCs and complement C3. Lyo‐SP significantly reduced bleeding in a tail‐clip model in thrombocytopenic mice. These studies establish the potential of Lyo‐SP as a shelf‐stable platelet surrogate for treating bleeding complications.

## Graphical abstract

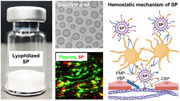

## 1 Introduction

**FIGURE 1:**
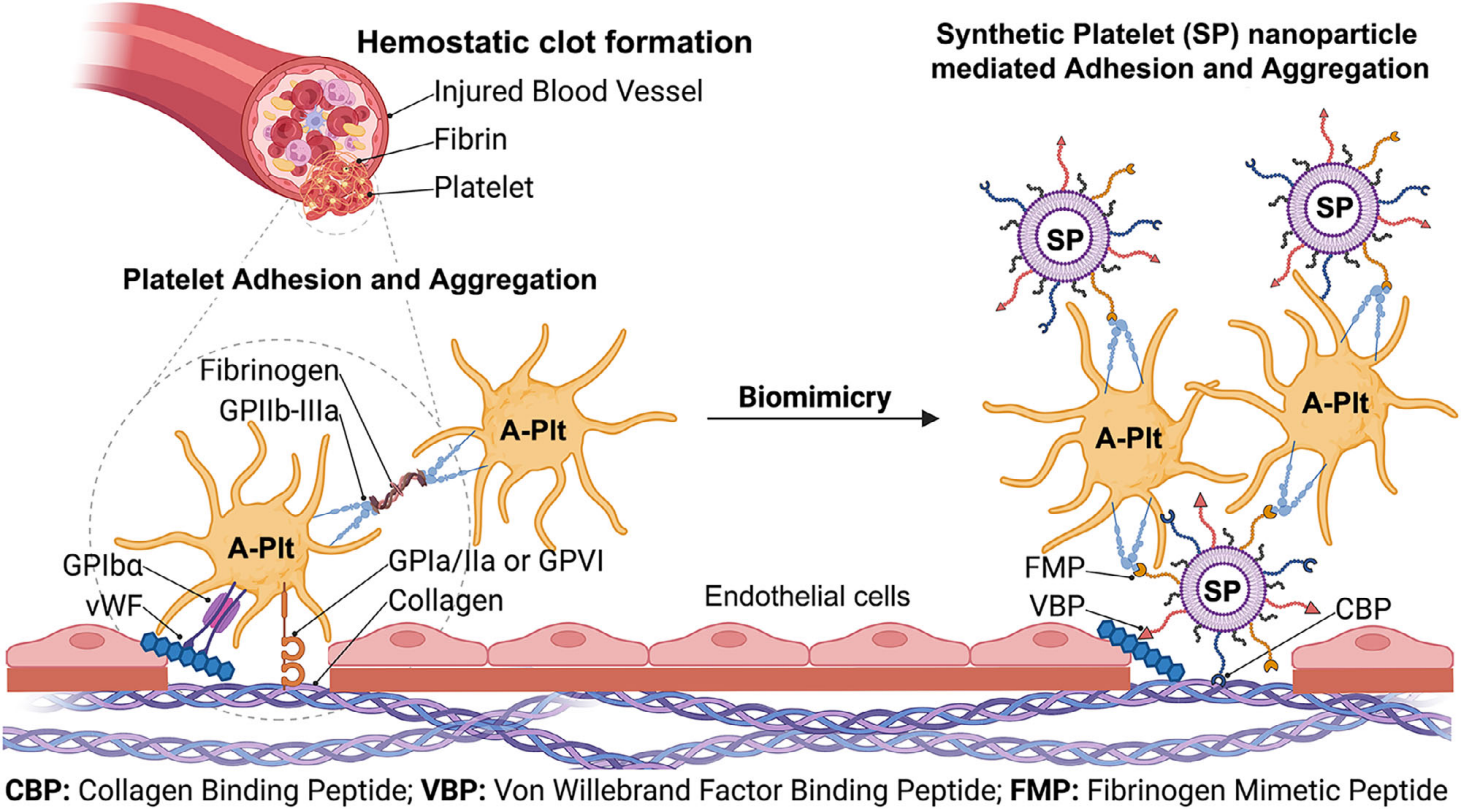
Synthetic Platelet (SP) nanoparticles mimic the primary hemostatic mechanisms of natural platelets. At the site of a vascular injury the primary hemostatic responses of natural platelets are to undergo rapid adhesion by binding to von Willebrand Factor (VWF, via platelet surface glycoprotein GPIbα) and collagen (via platelet surface glycoproteins GPIa/IIa and GPVI) leading to platelet activation; Activated platelets (A‐Plt) undergo aggregation via binding of fibrinogen to platelet surface glycoprotein GPIIb‐IIIa; Synthetic Platelet (SP) nanoparticles are peptide‐decorated liposomal constructs that mimic these hemostatic mechanisms of platelets by binding to VWF via VWF‐binding peptide (VBP), to collagen via collagen‐binding peptide (CBP) and to GPIIb‐IIIa via fibrinogen‐mimetic peptide (FMP). Figure created using Biorender.com.

The central physiological role of platelets is to form hemostatic clots to stop bleeding. Platelets do this by a complex concert of processes of which two primary mechanisms are: (1) rapid *adhesion* under flow to injury site‐exposed collagen via platelet glycoprotein GPIa/IIa and GPVI, and to von Willebrand Factor (VWF) via platelet glycoprotein GPIbα and (2) rapid *aggregation* of activated platelets via fibrinogen (Fg)‐mediated binding to platelet glycoprotein GPIIb/IIIa (Figure 1) [1, 2] Therefore, reduced platelet count or dysregulated platelet function results in bleeding complications [3, 4, 5]. Current treatments of such scenarios involve prophylactic (to reduce bleeding risk) or emergency (to mitigate active bleeding) transfusion of platelets obtained from donors [6, 7, 8, 9, 10]. As per FDA guidelines, such donor‐derived platelets are stored at room temperature (RT, ∼22°C) with gentle agitation [11, 12]. In these conditions there is increased risk of pathogenic bacteria growth beyond 5–7 days of storage, resulting in the short shelf life (5‐7 days) of RT platelets [13, 14, 15]. Additionally, platelet collection logistics constantly suffer from donor shortages and portability challenges [16, 17, 18, 19]. Consequently, platelet transfusion capabilities remain limited within resource‐intensive large hospitals and trauma centers, and are rarely available in small hospitals and ‘outside hospital’ settings (e.g. civilian or battlefield first responder use) to treat hemorrhaging patients [20, 21].

In recent years, pathogen reduction technologies (PRT) have been established to reduce contamination of platelet products and improve safety [22]. Studies are also ongoing to increase platelet shelf‐life and availability via reduced temperature processing and storage such as cold‐stored, cryopreservation and freeze‐drying [23, 24, 25, 26, 27, 28, 29, 30]. The FDA recently approved the practice of using cold‐stored platelets (CSP, stored at 4°C) to treat hemorrhage when RT platelets are not available, and clinical studies of CSP in trauma patients have been recently published [31]. Recent efforts have also reported functional refinement of donor platelets by utilization of mRNA‐loaded lipid nanoparticles for in vitro transfection [32]. While such endeavors can improve platelet availability and performance, these products are still dependent on donor‐derived platelets and thus remain affected by persistent donor shortages. Therefore, a parallel endeavor has emerged in developing ‘donor‐independent’ options. One arm of this effort is focused on production of biologic platelets in vitro from stem cells utilizing unique bioreactor approaches [33, 34, 35, 36]. The other arm has focused on developing *artificial* platelet surrogates by decorating synthetic biocompatible particles with ligand motifs that functionally mimic specific platelet mechanisms [37, 38, 39, 40, 41, 42]. In this second approach, we have developed a ‘synthetic platelet’ (SP) nanoconstruct utilizing heteromultivalent surface‐decoration of a liposomal nanoparticle with a combination of three peptides, namely VWF‐binding peptide (VBP), collagen‐binding peptide (CBP), and fibrinogen‐mimetic peptide (FMP) [43, 44]. We have already established the binding specificity of VBP to VWF and CBP to collagen for combinatorially mimicking the *adhesion* functionality, and of FMP to activated platelet integrin GPIIb/IIIa for mimicking the *aggregation* functionality of platelets [45, 46, 47]. These peptides are essentially considered the active pharmaceutical ingredients (API) in the SP design, and the integration of the platelet‐mimetic adhesion and aggregation functionalities results in SP uniquely recapitualing the primary hemostatic mechanisms of platelets (Figure 1).

The SP technology technology was initially manufactured as a sterile aqueous suspension and it has shown promising hemostatic capabilities in vitro and in several animal models of bleeding in vivo [48, 49, 50, 51, 52]. However, an inherent logistical limitation of SP is that like many other liposomal therapeutics it can suffer from physico‐chemical instabilities and reduced shelf‐life when stored as an aqueous suspension, which affects its translation into a storage‐stable platelet surrogate. Additionally, it is challenging for such sterile suspensions to be easily portable, especially in remote civilian and battlefield settings, due to large carry volume and environmental susceptibility. For several liposomal therapeutics, the pharmaceutical industry has established unique lyophilization (freeze‐drying) processes to enhance shelf‐stability while reducing the storage footprint and the need for specialized (e.g. cold‐chain) storage conditions [53, 54, 55]. An important aspect of the liposome lyophilization processes is the utilization of anhydrobiosis‐inspired carbohydrate‐based lyoprotectants that impart thermodynamic stability to the lipid vesicles, thereby protecting them from membrane packing instabilities and ice crystal induced damages during freeze‐drying [56, 57, 58]. In this framework, majority of lyophilized liposomal formulations that currently use lyoprotectant enrichment, are made of traditional *undecorated* phospholipid vesicles predominantly utilized for drug encapsulation, and there are only very limited reports on lyophilization and functional evaluation of *ligand‐decorated liposomes* [54, 59, 60, 61, 62, 63, 64]. Additionally, for the small subset of *ligand‐decorated liposomes* that have been reported to undergo lyophilization studies, the ligand molecules were used only for specific cell‐targeting, but not as APIs themselves. Also, none of these liposomal systems had heteromultivalent surface‐decoration with the combination of multiple bioactive peptides the way SP does. Based on this current state‐of‐art, we aimed to investigate the translational advancement of SP toward a lyophilized formulation (Lyo‐SP) using Hydroxy‐propyl‐beta‐cyclodextrin (HPβCD) as the lyoprotectant. HPβCD was selected from rigorous evaluation and screening of several different lyoprotectant molecules (Trehalose, Dextrose and HPβCD). A Lyo‐SP powder that is easily portable, can be stored long‐term at various ambient conditions without losing form and function, and can be rapidly reconstituted in sterile water for on‐demand use, can provide tremendous logistical benefit in therapeutic management of bleeding. With this premise, we report on the manufacture and in vitro characterization of Lyo‐SP for its physicochemical, hemostatic and biosafety properties. Furthermore, we demonstrate the hemostatic efficacy of Lyo‐SP in vivo using a tail‐clip bleeding model in thrombocytopenic mice.

## 2 Results

### 2.1 Lyo‐SP Manufacturing Preserves Particle Morphology and Stability

**FIGURE 2:**
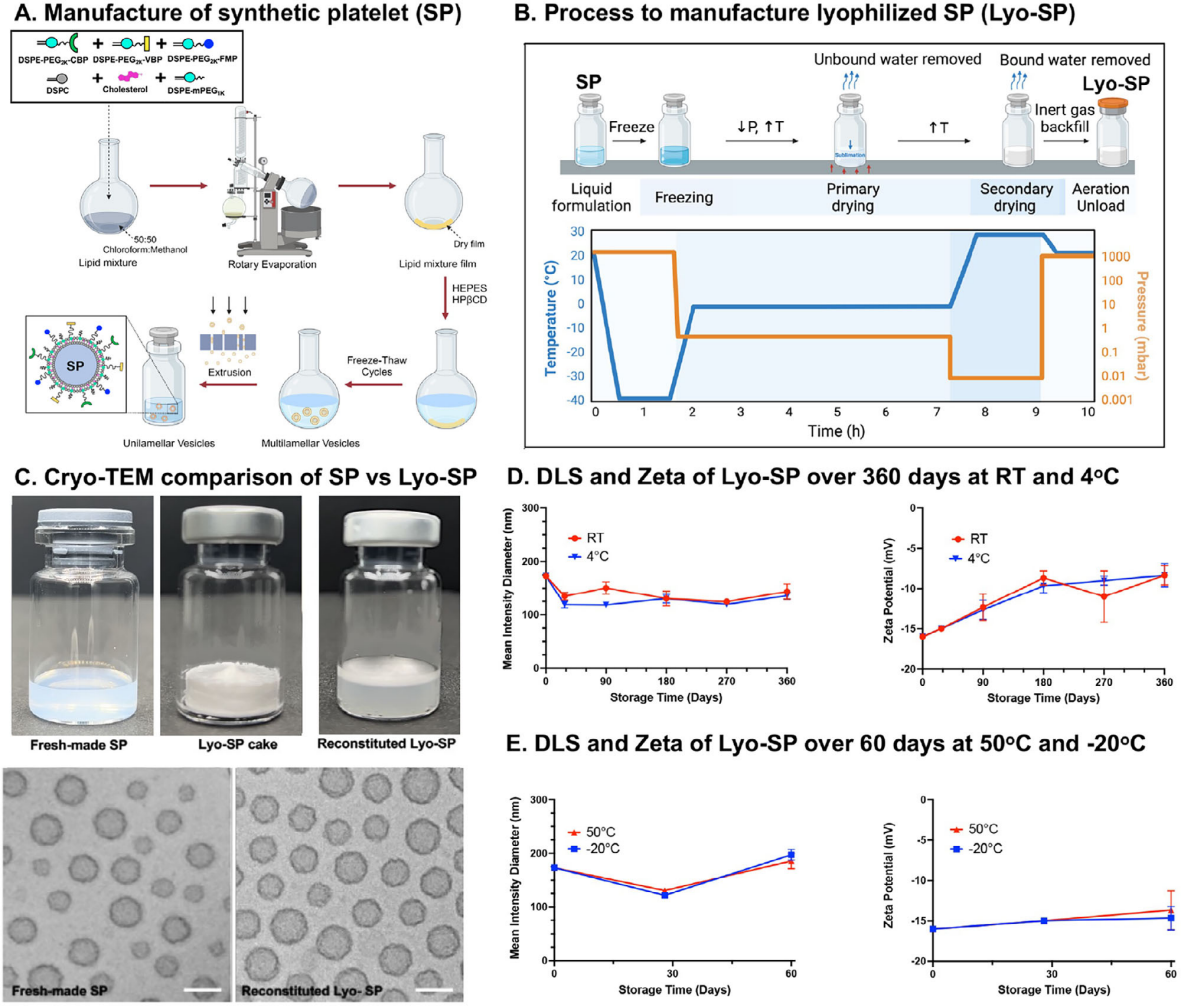
Lyo‐SP manufacturing preserves particle size charge morphology and stability over long‐term storage. (A) Manufacture of liposomal synthetic platelet (SP) nanoparticles with lyoprotectant (HPβCD in HEPES) using thin film rehydration followed by extrusion technique; (B) Process to generate lyophilized SP (Lyo‐SP) using two‐stage freeze‐drying under controlled temperature and pressure conditions (T: Temperature, P: pressure); (C) Physical appearance of SP, Lyo‐SP powder and aqueous‐reconstituted Lyo‐SP, and representative cryogenic transmission electron microscopy (cryo‐TEM) images of SP and reconstituted Lyo‐SP demonstrating that the unilamellar vesicular morphology of SP is conserved in reconstituted Lyo‐SP (scale bar = 100 nm); (D) Dynamic light scattering (DLS) and zeta potential characterization of Lyo‐SP size and charge when stored at room temperature (RT) and at refrigeration (4°C) show considerable stability for 1 year; (E) DLS and zeta potential characterization of Lyo‐SP stored at very high (50°C) and very low (‐20°C) temperatures show considerable stability of size and charge for 2 months.

The schematic for manufacturing SP is shown in Figure 2A and the process diagram for converting SP to Lyo‐SP is shown in Figure 2B. While Trehalose, Dextrose, and HPβCD were all investigated as potential lyoprotectants for Lyo‐SP manufacture, formulations with Trehalose or Dextrose failed to render optimal size conservation criteria post‐lyophilization and reconstitution, but HPβCD successfully achieved this (example size analysis data comparing SP manufactured with the three different lyoprotectants are shown in Figure S1). Representative photographs of freshly manufactured HPβCD‐enriched SP, Lyo‐SP processed therefrom, and aqueous‐reconstituted Lyo‐SP, are shown in Figure 2C along with representative cryo‐TEM images comparing fresh‐made SP vs. reconstituted Lyo‐SP. As evident from the cryo‐TEM images, SP showed unilamellar vesicular morphology and reconstituted Lyo‐SP retained this morphology, indicating membrane stability. Figure S2 shows representative DLS characterization data demonstrating comparable size distribution of HPβCD‐enriched SP vs. correspeonding reconstituted Lyo‐SP, and Figure S3 shows a table of representative DLS characterization data comparing SP vs. Lyo‐SP for five manufactured batches. As evident from these results, when manufactured with HPβCD as the lyopotectant, reconstituted Lyo‐SP maintained a hydrodynamic diameter of 156 ± 15 nm, zeta potential of ‐10 ± 2 mV, and polydispersity index (PDI) 0.20 ± 0.05 compared to SP (hydrodynamic diameter of 140 ± 13 nm, zeta potential ‐10 ± 2 mV, and PDI 0.15 ± 0.05). Movie M1 shows a representative video of Lyo‐SP reconstitution in saline, and Figure S4 shows representative data for turbidity and foam height analysis of reconstituted Lyo‐SP, indicating rapid and stable reconstitution, with foam subsiding within 5–10 min post‐reconstitution. Figure 2D shows DLS and zeta potential characterization of Lyo‐SP when stored at RT and 4°C refrigeration for 360 days, and Figure 2E shows this for Lyo‐SP stored at extreme hot (50°C) and cold (−20°C) temperatures for 60 days. As evident from this data, Lyo‐SP maintained stable size and charge when stored at RT or 4°C for at least 12 months (360 days), and at much higher (50°C) and lower (−20°C) temperatures for at least 2 months (60 days). Figure S5 shows cryo‐TEM image of Lyo‐SP after 6‐month and 12‐month storage at room temperature, further confirming that the HPβCD‐based lyophilization process allows significant morphological stability of Lyo‐SP over long storage periods, without requiring cold‐chain storage. Altogether, Lyo‐SP manufacture using HPβCD as the lyoprotectant demonstrated high reproducibility, post‐reconstitution retention of morphology, size and charge comparable to fresh‐made SP, and considerable long‐term storage stability under various temperature conditions.

### 2.2 Lyo‐SP Demonstrates Preservation of Platelet‐inspired Ligand Functions

**FIGURE 3:**
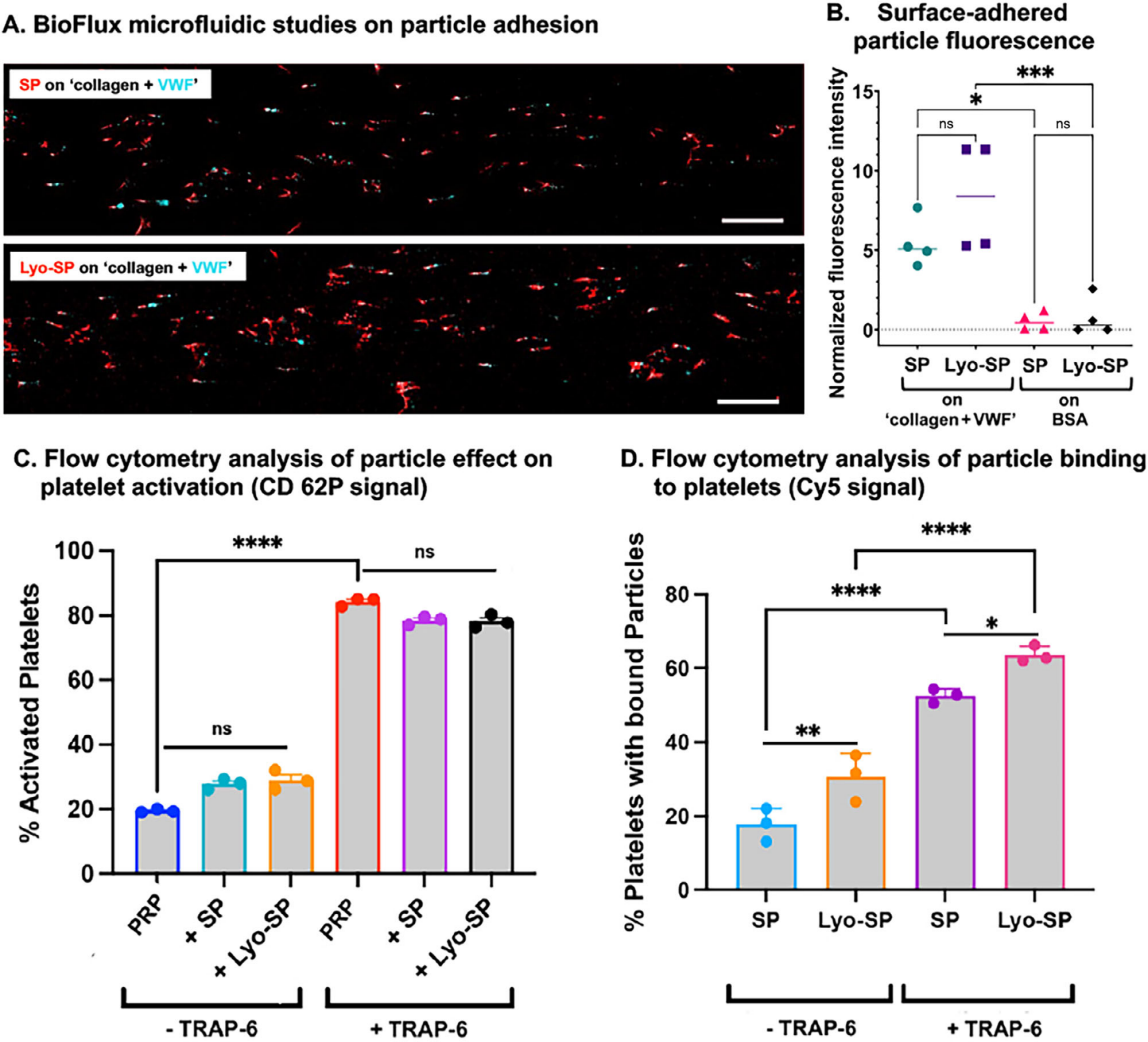
Lyo‐SP demonstrates preservation of platelet‐inspired ligand functions analyzed by microfluidics and flow cytometry. (A) Representative endpoint fluorescence images of SP vs. Lyo‐SP (red) adhesion on collagen‐coated BioFlux microfluidic channel surface in presence of soluble VWF (cyan) at high shear (60 dyn/cm^2^) demonstrating that the ‘VBP + CBP’‐decoration mediated platelet‐mimetic ‘VWF + collagen’‐adhesion properties of SP remain conserved in Lyo‐SP; (B) Quantitative analysis and comparison of surface‐adhered particle (SP vs. Lyo‐SP) fluorescence on collagen‐coated microfluidic channel in presence of soluble VWF vs. that on BSA‐coated microfluidic channel (negative control surface) confirm that both SP and Lyo‐SP undergo significantly enhanced adhesion on the ‘VWF + collagen’ compared to BSA, and the adhesion of Lyo‐SP on ‘VWF + collagen’ surface is statistically similar to that of SP; (C) Flow cytometry analysis of platelet activation (using platelet CD62P staining as activation marker) in presence of SP or Lyo‐SP shows that neither SP nor Lyo‐SP can themselves activate *predominantly inactive* platelets, and when platelet activation is induced using a standard agonist (TRAP‐6), neither SP nor Lyo‐SP inhibits platelet activation or induces additional activation, confirming that SP and Lyo‐SP do not impact platelet activation; (D) Flow cytometry analysis of particle binding to platelets (using Cy5 fluorescence from platelet‐bound particles) shows that both SP and Lyo‐SP undergo significantly enhanced binding to TRAP‐6‐activated platelets compared to platelets without TRAP‐6 activation, confirming that the FMP‐mediated enhanced binding of SP to *activated* platelets is conserved in Lyo‐SP. * p ≤ 0.05, *** p ≤ 0.001 and **** p ≤ 0.0001. ns: not significant. VWF: von Willebrand Factor. VBP: VWF‐binding peptide. CBP: Collagen‐binding peptide. BSA: Bovine serum albumin. TRAP‐6: Thrombin Receptor Activating Peptide 6.

As shown in Figure 1, the two salient platelet‐inspired bioactive properties of SP are: (i) *adhesion* to VWF (via VBP ligands) and collagen (via CBP ligands) under flow, and (ii) *aggregation* by interacting with the stimulated conformation of platelet integrin GPIIb/IIIa (via FMP ligands) [43, 44, 45, 46, 47]. Therefore, it is critical to characterize whether these ligand functionalities remain preserved in Lyo‐SP post‐reconstitution. Thus, the ‘collagen + VWF’ adhesion properties of SP vs. Lyo‐SP were compared using the BioFlux microfluidics system [65]. Figure S6 describes the BioFlux experimental set‐up for these studies. Prior to the microfluidic experiments, the collagen coating in the microfluidic channels as well as the VWF assembly on this collagen under high shear, were confirmed by fluorescence imaging. Figure S7A shows a representative image of a collagen‐coated channel showing surface‐immobilized collagen fibers (indigo blue). Movie M2 shows soluble VWF (cyan) assembly on such collagen‐coated channel surface, and Figure S7B shows a representative endpoint image of such WWF (cyan) assembled on the collagen‐coated channel (see Methods for experimental details). These data confirm the presence of both collagen and VWF in the microfluidic experiment set‐up to test the ‘VBP + CBP’‐mediated adhesion capability of SP vs. Lyo‐SP. Figure 3A shows representative endpoint images of SP vs. Lyo‐SP (Cy5 labeled, red fluorescent) adhered to collagen‐coated microfluidic channel surface in the presence of soluble VWF (cyan) at high shear flow (60 dyn/cm^2^), and Figure 3B shows quantitative analysis of surface‐bound SP vs. Lyo‐SP fluorescence normalized to SP or Lyo‐SP adhered on BSA‐coated microfluidic surface (negative control). Movies M3 and M4 show representative videos of SP and Lyo‐SP particles adhering to collagen‐coated microfluidic surface in presence of soluble VWF (particles in red, VWF in cyan). Figure S8 shows representative endpoint images and quantitatively analyzed data of 12‐month stored Lyo‐SP (Cy5 labeled, red fluorescent) adhering to the ‘collagen + VWF’‐coated microfluidic channel, confirming that the activity of the VBP and CBP peptides to bind to VWF and collagen respectively, remain preserved on Lyo‐SP upon storage. Altogether, these studies established that VBP‐mediated VWF‐binding and CBP‐mediated collagen‐binding functions of SP are conserved in Lyo‐SP.

The FMP‐mediated activated platelet‐binding functionality of SP and Lyo‐SP was tested using flow cytometry. Here, two specific aspects were analyzed: (i) whether SP or Lyo‐SP directly affects the activation ability of platelets, and (ii) whether SP has enhanced binding ability to activated platelets and this property is conserved in Lyo‐SP (see Methods for experimental details). As shown in Figure 3C, considering P‐selectin (CD62P antibody staining) as the activation marker, it was observed that in procuring *resting* platelets via serial centrifugation and resuspension steps, a small percentage of platelets gets unavoidably activated at baseline, as also reported elsewhere [66]. Incubation of such predominantly *resting* platelets (without platelet PAR‐1 agonist TRAP‐6) with SP or Lyo‐SP did not increase P‐selectin signal, suggesting that SP or Lyo‐SP did not induce additional platelet activation. When platelets were incubated with TRAP‐6, a significant percentage of platelets became P‐selectin positive, confirming high activation. Incubation of SP or Lyo‐SP with TRAP‐6‐activated platelets did not inhibit this activation or induce additional activation, indicating that SP or Lyo‐SP did not impact platelet activation. Figure 3D shows subsequent analysis of fluorescent (Cy5 labeled) SP or Lyo‐SP binding to predominantly *resting* platelets (without TRAP‐6 activation) vs. predominantly activated platelets (with TRAP‐6 activation). Without TRAP‐6 incubation, the percentage of platelets showing ‘bound nanoparticles’ was low (17.73 ± 2.6 % for SP and 30.63 ± 3.6 % for Lyo‐SP). In comparison, for TRAP‐6‐activated platelets, the percentage of platelets with ‘bound nanoparticles’ increased to 52.53 ± 1.1 % for SP and 63.63 ± 1.3 % for Lyo‐SP. These results indicate that the ability of SP to undergo FMP‐mediated enhanced binding on *activated* platelets is conserved in Lyo‐SP. An additional interesting observation in the flow cytometry analysis was that, while both SP and Lyo‐SP showed significantly higher binding to TRAP‐6‐activated platelets compared to platelets without TRAP‐6 activation, there was also a statistical difference between SP vs. Lyo‐SP binding to platelets. This difference can be attributed to two possibilities: (i) The potential variability in overall platelet activation status (and GPIIb/IIIa surface‐density on activated platelets) between donors, as the experiments were conducted with biological replicates, and (ii) Slight changes in the presentation profile of surface‐conjugated bioactive ligands in reconstituted Lyo‐SP. Irrespective of these modest variance possibilities, the consistent observation was that Lyo‐SP conserved the ability of SP to undergo enhanced binding to activated platelets, which is a critical component of its hemostatic platelet‐aggregatory mechanism. Figure S9 shows additional flow cytometry analysis of three different batches of Lyo‐SP binding to activated platelets using different donors on different days for each batch. As evident from the data, the different batches of Lyo‐SP show some donor‐dependent variability in binding to *resting* platelets vs. *activated* platelets, but overall all three batches of Lyo‐SP showed significantly enhanced binding to TRAP‐6 activated platelets compared to platelets without TRAP‐6 activation. Figure S10 shows flow cytometry data of 12‐month stored Lyo‐SP binding to predominantly resting platelets vs. to TRAP‐6‐activated platelets, confirming that the FMP activity on Lyo‐SP to bind integrin GPIIb/IIIa on activated platelets is conserved over the 12‐month storage period. Altogether, these results indicate batch‐to‐batch reproducibility and conservation of Lyo‐SP binding to activated platelets via preservation of the FMP activity.

### 2.3 Lyo‐SP Demonstrates Retention of SP Hemostatic Response in human Plasma In Vitro

**FIGURE 4:**
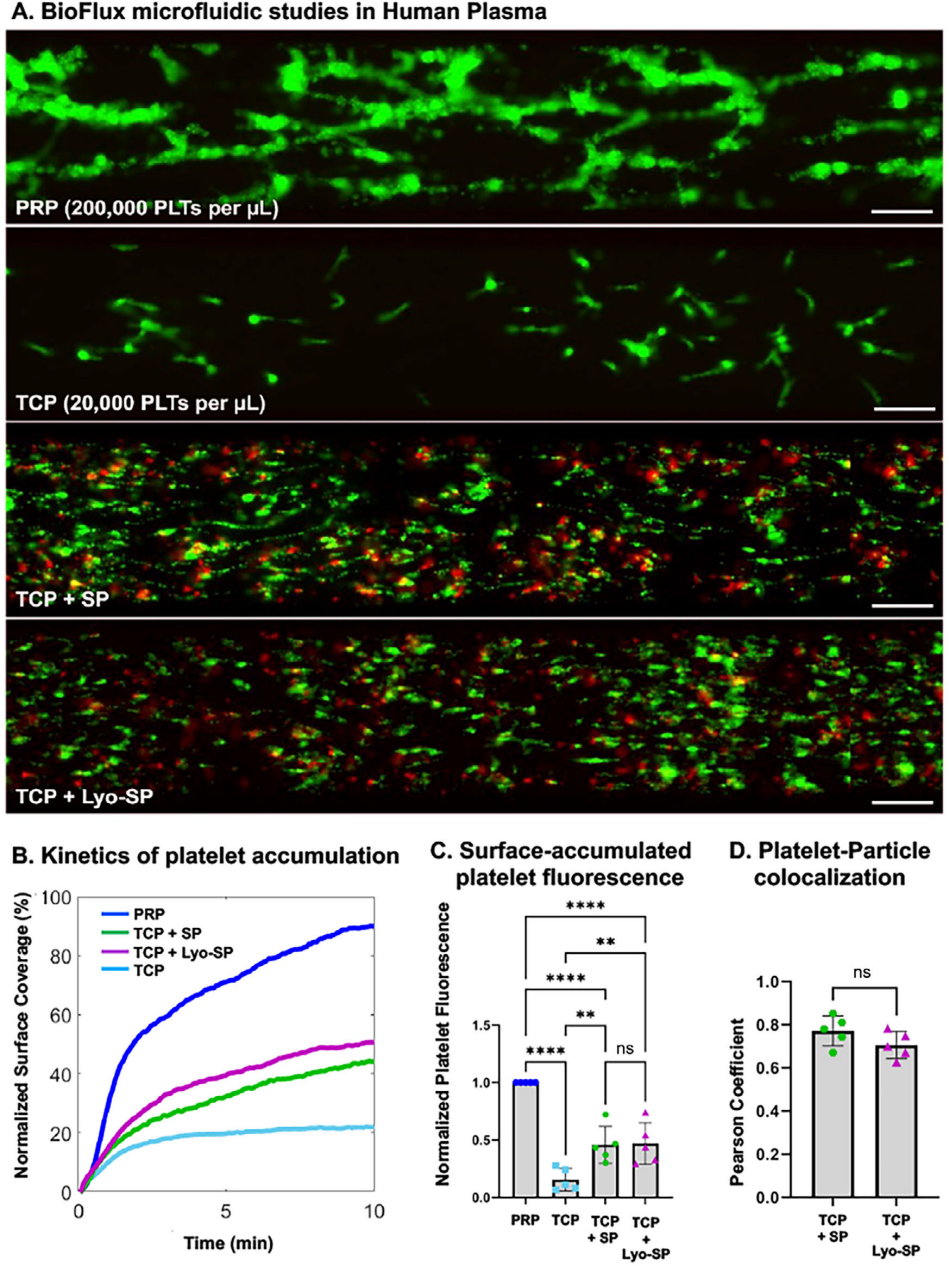
Lyo‐SP retains the hemostatic effect of SP in human plasma analyzed by in vitro microfluidic studies in a thrombocytopenic setting. (A) Representative endpoint images of BioFlux microfluidic studies showing platelet accumulation (green fluorescence from Calcein AM) on collagen‐coated microfluidic channel in presence of soluble VWF under high shear flow (60 dyn/cm^2^) of healthy platelet‐rich plasma (PRP, positive control) vs. thrombocytopenic plasma (TCP) vs. TCP treated with SP vs. TCP treated with Lyo‐SP, demonstrating that compared to PRP the platelet accumulation is substantially reduced in TCP and treatment with SP or Lyo‐SP considerably rescues platelet accumulation; (B) Representative kinetic curves of platelet accumulation on the microfluidic channels showing that the platelet accumulation kinetics is substantially reduced in TCP compared to PRP, and treatment with SP or Lyo‐SP can rescue this process; (C) Quantitative analysis of surface‐accumulated platelet fluorescence (Calcein AM signal) confirm that platelet accumulation is significantly reduced in TCP condition compared to PRP condition, and treatment of TCP with SP or Lyo‐SP can significantly rescue platelet accumulation, with the rescue effect statistically similar between SP and Lyo‐SP; (D) Quantitative analysis of fluorescence co‐localization (Pearson Coefficient) between platelets (green) and particles (red) confirm that the co‐localization remains statistically similar between SP and Lyo‐SP. ** p ≤ 0.01, and **** p ≤ 0.0001. ns: not significant.

The integrated functionality of *adhesion* (to VWF and collagen) and *aggregation* (to activated platelet GPIIb/IIIa) provides SP the capability to rescue hemostatic response even when the native platelet count is reduced (i.e. thrombocytopenia), and this functional aspect was compared between SP vs. Lyo‐SP using BioFlux microfluidics. Figure S11 describes the experimental set‐up for these studies. Figure 4A shows representative endpoint images of Calcein AM‐stained (green fluorescent) platelet accumulation and platelet co‐localization with Cy5‐labeled (red fluorescent) SP or Lyo‐SP, on collagen‐coated microfluidic channels in the presence of soluble VWF at high shear flow (60 dyn/cm^2^). As evident from the images, compared to PRP (200,000 platelets per µL), the platelet accumulation is significantly reduced in thrombocytopenic plasma (TCP; 20,000 platelets per µL), indicated by the substantial decrease in green fluorescent platelets accumulated on the channel surface. Treatment of TCP with SP considerably rescued platelet coverage on the channel surface, and treatment with Lyo‐SP showed a similar rescue of platelet coverage. Movies M5, M6, M7 and M8 show representative videos of platelet accumulation on the microfluidic channel surface in PRP, TCP, ‘TCP + SP’ and ‘TCP + Lyo‐SP’ conditions, respectively. Figure 4B shows representative kinetic data of platelet coverage on the channel surface, indicating that compared to PRP (indigo), the platelet accumulation kinetics is substantially reduced in TCP (cyan), and treatment of the TCP condition with SP (green) or Lyo‐SP (magenta) considerably rescues platelet accumulation kinetics on the channel surface. Figure 4C shows endpoint statistical analysis of surface‐adhered platelet fluorescence from such studies, where platelet fluorescence was significantly reduced in TCP condition compared to PRP (p < 0.0001). Treatment of TCP condition with SP significantly rescued surface‐accumulated platelet fluorescence (p = 0.01), and treatment with Lyo‐SP similarly rescued surface‐accumulated platelet fluorescence (p = 0.0075). Figure S12 shows additional results for the effect of three different batches of Lyo‐SP tested in TCP condition using human plasma from five different blood donors for each batch in the BioFlux microfluidic setting. As evident from the results, Lyo‐SP maintains reproducibility in its hemostatic effect of rescuing surface‐accumulated platelet fluorescence. Figure 4D shows colocalization between the green platelets and the red nanoparticles accumulated on the channel surface, demonstrating that the platelet‐to‐particle colocalization of SP remains conserved in Lyo‐SP. Figure S13A shows representative endpoint images of green fluorescent platelet accumulation in BioFlux microfluidic studies in TCP condition compared to that in ‘TCP + Lyo‐SP’ condition with Lyo‐SP freshly manufactured (0 months) vs. 6‐month stored Lyo‐SP vs. 12‐month stired Lyo‐SP. Figure S13B shows representative kinetic data of platelet coverage where compared to PRP (indigo), the platelet accumulation kinetics is substantially reduced in the TCP (cyan), and treatment of this TCP condition with freshly manufactured Lyo‐SP (magenta) vs. 6 month‐stored Lyo‐SP (brown) showed comparable rescue in the platelet accumulation kinetics. Treatment of TCP condition with 12 month‐stored Lyo‐SP showed a slighltly reduced rescue profile for platelet accumulation kinetics but still higher than the TCP condition. Figure S13C shows endpoint statistical analysis of surface‐adhered platelet fluorescence from these studies, further confirming that freshly manufactured Lyo‐SP and 6‐month stored Lyo‐SP can significantly and comparably rescue platelet accumulation in TCP condition, and the 12‐month stored Lyo‐SP also rescues platelet accumulation even though it did not reach statistical significance. Overall, these results demonstrate that treatment of the TCP condition with Lyo‐SP can rescue the hemostatically important outcome of platelet accumulation on an injury site‐simulating surface at levels similar to treatment with SP, this effect is reproducible for different batches of Lyo‐SP, and is substantially conserved in Lyo‐SP stored for long time periods (6–12 months). Therefore, the results indicate that the ‘VBP + CBP + FMP’‐mediated platelet‐inspired mechanisms of SP are extensively and reproducibly conserved in Lyo‐SP in long‐term storage.

### 2.4 Multiparamaetric Evaluation in Human Blood exhibits Hemostatic Effect of Lyo‐SP

Since the peptide‐mediated platelet‐mimetic adhesive and aggregatory functions of SP are strongly conserved in Lyo‐SP as demonstrated by the above‐described studies and Lyo‐SP is the lead therapeutic system envisioned to be ultimately clinically translated, therefore subsequent in vitro and in vivo studies were conducted with Lyo‐SP. The first goal was to assess whether the hemostatic effect of Lyo‐SP is maintained in a whole blood (WB) setting. This is important because in real‐world applications Lyo‐SP is envisioned to be dosed intravenously in patients with bleeding complications, and if WB components interfere with Lyo‐SP function, then its hemostatic benefit will be compromised. For WB‐based studies, three complementary assays were used, namely, BioFlux microfluidics, Total Thrombus Analysis System (T‐TAS) [67] and Rotational Thromboelstometry (ROTEM) [68]. In these studies healthy WB was used as the positive control, and thrombocytopenic WB (TC‐WB) was used as the negative control.

**FIGURE 5:**
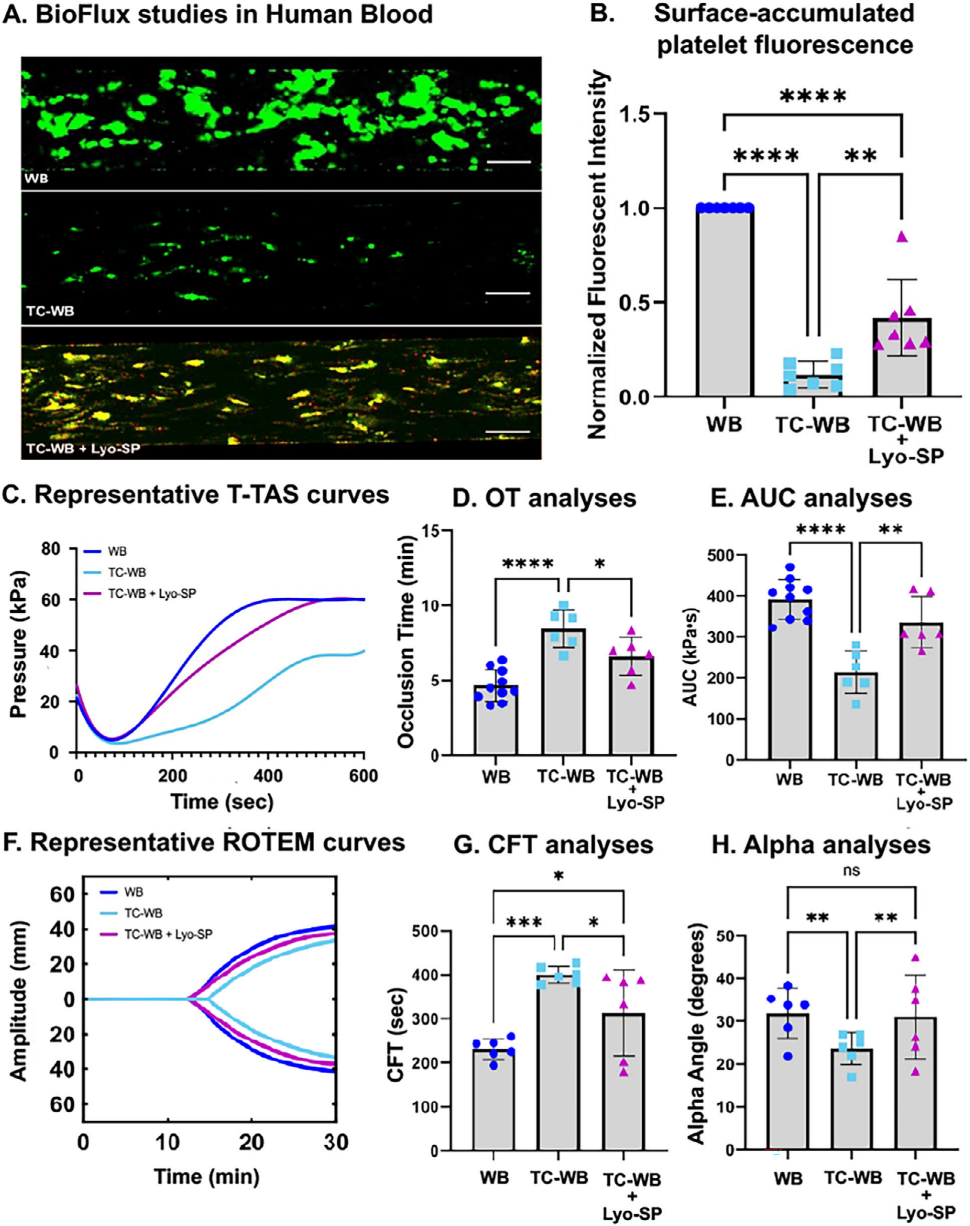
Lyo‐SP demonstrates hemostatic effect in multipramaetric evaluation using human blood in vitro in a thrombocytopenic setting. (A) Representative endpoint images of BioFlux microfluidic studies showing platelet accumulation (green Calcein AM) on collagen‐coated microfluidic channel in presence of soluble VWF under high shear flow (60 dyn/cm^2^) of healthy whole blood (WB, positive control) vs. thrombocytopenic whole blood (TC‐WB) vs. TC‐WB treated with Lyo‐SP, demonstrating that compared to WB the platelet accumulation is substantially reduced in TC‐WB and treatment with Lyo‐SP considerably rescues platelet accumulation, confirming that hemostatic effect of Lyo‐SP remains viable in whole blood; (B) Quantitative analysis of surface‐accumulated platelet fluorescence (Calcein AM signal) confirm that platelet accumulation is significantly reduced in TC‐WB condition compared to the healthy WB, and treatment of TC‐WB with Lyo‐SP can significantly rescue platelet accumulation; (C) Representative pressure vs. time curves from Total Thrombus‐formation Analysis System (T‐TAS) studies using platelet chip (PL‐Chip) on WB vs. TC‐WB vs. TC‐WB treated with Lyo‐SP demonstrate that the channel occlusion due to platelet‐mediated clot formation is substantially compromised in TC‐WB compared to WB, and treatment of TC‐WB with Lyo‐SP rescues the channel occlusion (i.e. clot formation) profile; (D) Quantitative analysis of Occlusion Time (OT) parameter in T‐TAS analysis demonstrates that OT is significantly compromised in TC‐WB condition compared to WB, and treatment of TC‐WB with Lyo‐SP rescues this parameter; (E) Quantitative analysis of Area‐under‐Curve (AUC) parameter in T‐TAS analysis demonstrates that the AUC parameter (overall clot formation) is significantly compromised in TC‐WB condition compared to WB, and treatment of TC‐WB with Lyo‐SP rescues this parameter; (F) Representative amplitude vs. time curves (TEM‐ograms) from Rotational Thromboelastometry (ROTEM) analysis on WB vs. TC‐WB vs. Lyo‐SP‐treated TC‐WB demonstrate that the clot viscoelastometric profile is substantially compromised in TC‐WB condition compared to WB, and treatment of TC‐WB with Lyo‐SP rescues this profile; (G) Quantitative analysis of Clot Formation Time (CFT) parameter in ROTEM demonstrates that CFT (reflective of initial clot formation) is significantly compromised in TC‐WB condition compared to WB, and treatment of TC‐WB with Lyo‐SP rescues this parameter; (H) Quantitative analysis of Alpha angle (α) parameter in ROTEM demonstrates that α angle (reflective of clot growth *rate*) is significantly compromised in TC‐WB condition compared to WB, and treatment of TC‐WB with Lyo‐SP rescues this parameter. * p ≤ 0.05, ** p ≤ 0.01, *** p ≤ 0.001 and **** p ≤ 0.0001. ns: not significant.

Figure 5A shows representative endpoint images of BioFlux microfluidic studies and Figure 5B shows statistically analyzed quantitative data of accumulated platelet fluorescence on the microfluidic channel surface in WB vs. TC‐WB vs. ‘TC‐WB + Lyo‐SP’ conditions in these studies. Movies M9, M10 and M11 show representative videos of platelet accumulation on the microfluidic channel surface in WB vs. TC‐WB vs. ‘TC‐WB + Lyo‐SP’ respectively. As evident from these results, the platelet accumulation on the microfluidic channel surface is significantly reduced in TC‐WB compared to WB (p < 0.0001). Treatment of TC‐WB with Lyo‐SP significantly rescued (p = 0.0017) platelet accumulation, indicating that the platelet‐mimetic hemostatic mechanisms of Lyo‐SP remain viable in a whole blood setting.

Figure S14A,B describe the instrumentation schematic and characteristic data profile for the T‐TAS assay using the platelet (PL) chip. In this assay, whole blood samples are allowed to flow through microfluidic capillary channels coated with collagen to enable the temporal evaluation of platelet‐mediated clot formation [67]. The growing platelet thrombus causes occlusion of the microcapillary channels, causing an increase in the flow pressure within the assay chip that is continuously monitored by a pressure sensor. Pressure vs. time data are calculated automatically for 10 min or until the pressure reaches 60 kPa above the baseline pressure. Figure 5C shows representative pressure vs. time curves for healthy WB, TC‐WB and ‘TC‐WB + Lyo‐SP’ conditions in T‐TAS analyses, Figure 5D shows occlusion time (OT) and Figure 5E shows area‐under‐curve (AUC) results analyzed from such studies. As evident from the results, OT was significantly increased (p < 0.0001) and AUC was significantly reduced (p < 0.0001) in TC‐WB compared to healthy WB, indicating that platelet depletion resulted in compromised clotting kinetics and clot growth. Treatment of TC‐WB with Lyo‐SP significantly rescued OT (p = 0.036) and AUC (p = 0.0022) parameters. Figure S14C also shows Occlusion Start Time (OST) analysis from these studies, demonstrating that compared to healthy WB, there was a significant delay in the initiation of channel occlusion for TC‐WB, and treatment of TC‐WB with Lyo‐SP showed a substantial rescue from this initial delay, ultimately resulting in the significant rescue of OT. These studies further confirm that the ability of Lyo‐SP to rescue clotting kinetics and clot growth in thrombocytopenic setting remains viable in whole blood.

Figure S15A,B describe the instrumentation schematic and characteristic data profile for the ROTEM assay. A typical ROTEM experiment involves placing WB in a cuvette and lowering an oscillating pin into this cuvette such that the pin oscillation is impeded by coagulating blood, and this mechanical impedance is optically detected to obtain specific parameters reflective of clot kinetics [68]. Lyo‐SP was evaluated using the Non‐activated Thromboelastometry (NATEM) mode in ROTEM, where coagulation of recalcified citrated blood occurs without the supplemental addition of any specific coagulation factor activating reagent. The rationale behind using the NATEM modality is that due to the absence of extraneous coagulation activating factors the initial kinetics of clot formation in this modality remains more sensitive to platelet‐mediated procoagulant activity and thus impacted by platelet count. Figure 5F shows representative ROTEM curves (TEM‐ograms) for healthy WB, TC‐WB and ‘TC‐WB + Lyo‐SP’ conditions, Figure 5G shows analyzed clot formation time (CFT in seconds, reflective of initial time for clot formation) and Figure 5H shows analyzed alpha angle (α, reflective of the clot growth *rate*), from these studies. As evident from the results, depletion of platelets (i.e. TC‐WB) resulted in substantial compromise of coagulation kinetics, as reflected by a significant increase (p = 0.0003) in CFT (reflecting delayed clot initiation) and a significant decrease (p = 0.0047) in α angle (reflecting reduced clot growth *rate*), compared to WB. Treatment of TC‐WB with Lyo‐SP was able to significantly rescue coagulation kinetics, by reducing CFT (p = 0.0294) and increasing α angle (p = 0.0095 value). Figure S15C also shows the analysis of ‘clot amplitude 10 min after clotting time’ (A10) parameter from these studies, demonstrating that A10 for platelet‐depleted WB (i.e. TC‐WB) was significantly reduced compared to healthy WB (indicating compromised initial clot growth), and treatment of TC‐WB with Lyo‐SP significantly rescued this parameter. Therefore, these results further establish that the hemostatic effect of Lyo‐SP to rescue clot kinetics remains viable in a human whole blood setting.

### 2.5 Lyo‐SP Demonstrates Biosafety in Relevance to Vascular Environment

As Lyo‐SP is envisioned to be administered intravenously for hemostatic therapy, it is important to assess whether it has any negative biological effects in the vascular environment, such as hemolysis of RBCs, activation of endothelial cells (characteristic of inflammatory effect), activation of neutrophils (characteristic of innate immune activation) and activation of complement factor like C3 (another characteristic of innate immune activation). Therefore, these aspects were studied in vitro by incubating therapeutic dose ranges of Lyo‐SP with human blood (for hemolysis assessment), human plasma (for complement C3 activation assessment), human vascular endothelium, and human neutrophils (see Methods for experimental details).

**FIGURE 6:**
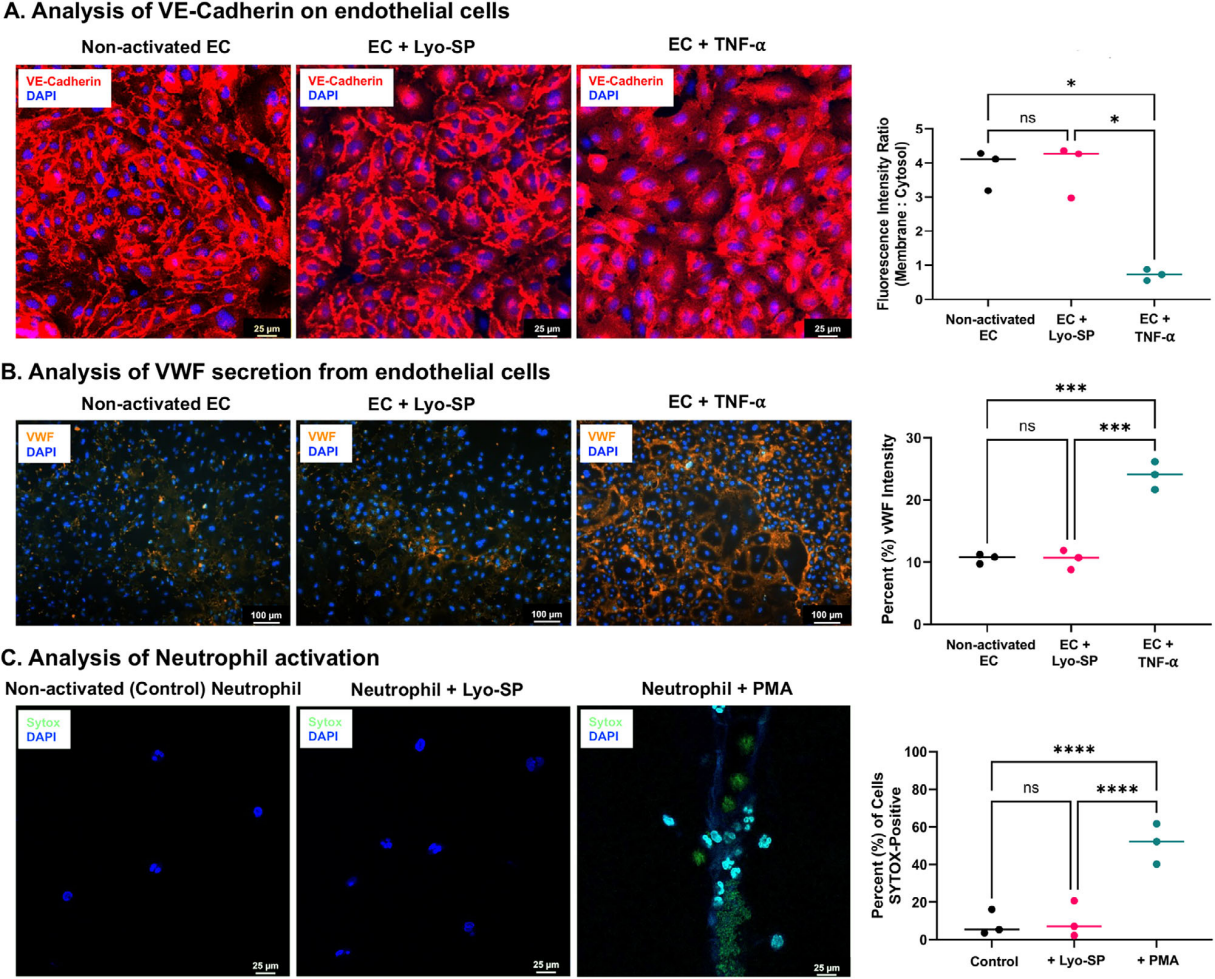
Lyo‐SP exhibits biosafety in relevance to vascular environment in vitro. (A) Representative fluorescence images and analyzed fluorescence intensity ratio of VE‐Cadherin at the junctional membrane versus cytosol of HUVECs demonstrate that incubation with Lyo‐SP maintains the junctional residence of VE‐Cadherin similar to non‐activated cells whereas for TNF‐α‐incubated cells the presence of VE‐Cadherin at cell junction is significantly reduced and its presence in cell cytosol is significantly increased as a marker of barrier disruption; (B) Representative fluorescence images and analyzed fluorescence intensity of HUVEC‐secreted VWF indicate that Lyo‐SP‐incubated cells have minimal VWF secretion similar to non‐activated cells whereas TNF‐α‐incubated cells have significantly increased VWF secretion as a marker of endothelial activation; (C) Representative fluorescence images and analyzed fluorescence intensity of SYTOX Green positive human neutrophils indicate that Lyo‐SP‐incubated neutrophils have minimal SYTOX staining similar to non‐activated neutrophils whereras PMA‐incubated neutrophils show significantly higher SYTOX staining as a marker of neutrophil activation and extracellularized DNA. * p ≤ 0.05, *** p ≤ 0.001 and **** p ≤ 0.0001. ns: not significant.

Figure 6A shows representative fluorescent images comparing VE‐Cadherin (red) staining on non‐activated endothelium vs. Lyo‐SP‐incubated endothelium vs. TNF‐α‐incubated endothelium (positive control for endothelial activation), and the corresponding quantitative analysis of VE‐Cadherin fluorescence intensity between membrane vs. cytosol of the cells. VE‐Cadherin is a crucial transmembrane protein in endothelial cells usually localized at the inter‐endothelial junctions for healthy endothelium, whereas upon endothelial activation it is shed and delocalized into the cytosol [69]. As evident from the images, incubation with Lyo‐SP did not affect the VE‐Cadherin fluorescence at the inter‐endothelial junctions while incubation with TNF‐α substantially delocalized VE‐Cadherin fluorescence into the cytosol indicating endothelial activation and loss of junctional integrity. The quantitative analysis showed that the VE‐Cadherin fluorescence intensity ratio between cell membrane and cytosol remained high for Lyo‐SP‐incubated endothelium, at levels statistically similar to non‐activated endothelium, while for TNF‐α‐incubated endothelium this ratio was significantly reduced as majority of the VE‐Cadherin fluorescence was delocalized from membrane to cytosol. Figure 6B shows representative fluorescent images comparing VWF (orange fluorescence) staining on non‐activated endothelium vs. Lyo‐SP‐incubated endothelium vs. TNF‐α‐incubated endothelium (positive control for endothelial activation), and the corresponding quantitative analysis of VWF fluorescence intensity (as a percent of imaged surface area) for the three groups. VWF is stored in the Weibel‐Palade bodies in the cytosol of healthy endothelium and is secreted only upon endothelial activation [70]. As evident from the images, VWF staining was minimal for Lyo‐SP‐incubated endothelium but was significantly enhanced for TNF‐α‐incubated endothelium. The quantitative analysis showed that the VWF fluorescence intensity for Lyo‐SP‐incubated endothelium was similar to non‐activated endothelium (low baseline fluorescence of ∼10% of surface area) but that for TNF‐α‐incubated endothelium was significantly higher. Altogether, these studies indicate that Lyo‐SP did not have any activating effect on endothelial cells, which is an important biosafety consideration.

Figure 6C shows representative fluorescent images comparing nuclear stain (blue DAPI, green SYTOX) on non‐activated neutrophils vs. Lyo‐SP‐incubated neutrophils vs. PMA‐incubated neutrophils (positive control for neutrophil activation), and the corresponding quantitative analysis of percent of neutrophils that stained positive for SYTOX across the three groups. PMA‐induced neutrophil activation results in subsequent formation of neutrophil extracellular traps (NETs) which consist of extracellularized DNA [71]. In the NET formation process for activated neutrophils the cell membrane gets permeabilized, allowing staining with SYTOX which is otherwise impermeable to healthy non‐activated neutrophils [72]. As evident from the images, Lyo‐SP‐incubated neutrophils barely showed any SYTOX staining (similar to non‐activated neutrophils), wheras PMA‐incubated neutrophils showed substantial SYTOX staining (blue DAPI merged with green SYTOX showing as turquoise) with some released NETs clearly visible. The quantitative analysis corroborate these results showing minimal SYTOX positivity for non‐activated neutrophils and Lyo‐SP‐incubated neutrophils, but significant SYTOX positivity for PMA‐incubated neutrophils. These studies indicate that Lyo‐SP did not have any activating effect on neutrophils. Furthermore, Figure S16 shows quantitative analysis of Complement C3 activation (to C3a and C3b) as analyzed by ELISA comparing control plasma vs. Lyo‐SP‐incubated plasma vs. positive control‐incubated plasma (LPS used as positive control for C3 activation to C3a and Cobra Venom Factor used as positive control for C3 activation to C3b). High activation of C3 can be indicative of immune activation and anaphylaxis risks [73]. As evident from the results, Lyo‐SP did not demonstrate any major risk for this. Figure S17 shows analysis of hemolytic risk for Lyo‐SP, indicating that Lyo‐SP does not cause significant hemolysis and therefore potentially safe toward RBC health. Altogether, these data indicate that therapeutic dose of Lyo‐SP poses minimal biosafety risks in the vascular environment which is important in the context of systemic safety considerations for intended in vivo applications.

### 2.6 Lyo‐SP Reduces Bleeding in Tail‐cip Model in Thrombocytopenic Mice

**FIGURE 7:**
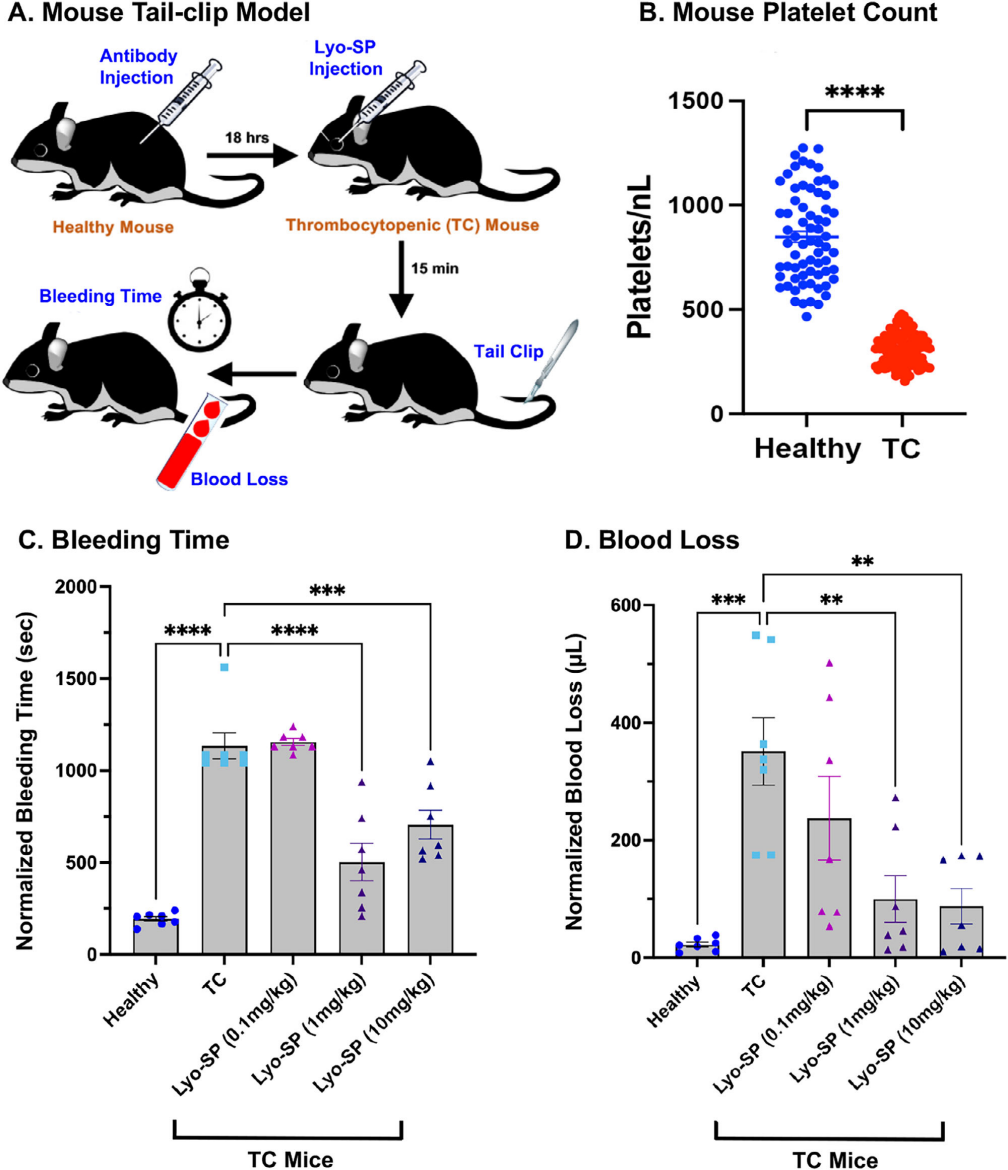
Lyo‐SP reduces bleeding time and blood loss in a tail‐clip bleeding model in thrombocytopenic mice. (A) Experimental design schematic of mouse tail‐clip bleeding model where mice were first made thrombocytopenic (TC mouse) by intraperitoneal dosing of anti‐GPIbα antibody to induce platelet clearance from circulation, followed by dosing of Lyo‐SP treatment and 15 min later a surgical tail‐clip injury was performed to measure bleeding time and blood loss (n = 7 per group); (B) Platelet count analysis confirming that the antibody injection results in significant drop in platelet count resulting in the TC mice; (C) Quantitative analyses of bleeding time and (D) blood loss show that compared to healthy mice the TC mice exhibited a significantly increased bleeding time as well as blood loss due to compromised hemostatic ability stemming from the reduced platelet count; Treating TC mice with Lyo‐SP at 0.1 mg/kg did not reduce bleeding time or blood loss, but with Lyo‐SP at 1 mg/kg and 10 mg/kg significantly reduced both bleeding time and blood loss. * p ≤ 0.05, ** p ≤ 0.01, *** p ≤ 0.001 and **** p ≤ 0.0001.

Building on the hemostatic effects observed in our in vitro studies, Lyo‐SP was studied in vivo to evaluate hemostatic efficacy in genetically identical strains of male C57 black 6 (C57/BL6) mice. In these studies, platelet depletion (i.e. thrombocytopenia) was induced by anti‐GPIbα antibody injection, and standard tail‐clip bleeding was used to assess bleeding time and blood loss [74, 75]. Figure 7A shows the experimental set‐up for the mouse studies. As shown in Figure 7B, anti‐GPIbα antibody injection resulted in a significant decrease in platelet count in the mice, thus confirming severe thrombocytopenia (TC). Figure 7C shows the bleeding time analyses and Figure 7D shows the blood loss analyses from the tail‐clip studies in healthy mice vs. TC mice vs. TC mice treated with either 0.1 mg/kg or 1 mg/kg or 10 mg/kg Lyo‐SP (n = 7 per geroup) administered retro‐orbitally 15 min prior to injury. Figure S18 also shows the bleeding time data in Kaplan‐Meier format. As evident from the results, healthy mice had a tail bleeding time of 193 ± 13 s, while bleeding time for TC mice increased significantly to 1155 ± 52 s. Correspondingly, healthy mice showed a blood loss of 22 ± 4.1 µl while TC mice showed a significantly increased blood loss of 390 ± 53 µl. Treatment of the TC mice with Lyo‐SP at increasing doses (0.1, 1 or 10 mg/kg), showed that the dose of 0.1 mg/kg was sub‐optimal to have a statistically significant effect on reducing bleeding time. However, Lyo‐SP doses at 1 mg/kg and 10 mg/kg were both able to significantly reduce bleeding time (502 ± 101 s and 706 ± 79 s respectively) and blood loss (100 ± 40 µl and 88 ± 30 µl respectively). It is important to note that, in this species and model, increasing the dose from 1 mg/kg to 10 mg/kg did not increase further hemostatic response and this can be an indication of dose effect saturation in the current model. Figure S19 shows bleeding time and blood loss data for TC mice treated with non‐lyophilized SP, and as evident from the data, SP dose of 1 mg/kg significantly reduced bleeding time and blood loss in TC mice at levels comparable to that observed for Lyo‐SP‐treated TC mice, confirming that the in vivo hemostatic effect of SP remains conserved in Lyo‐SP. Additionally, Figure S20 shows representative histology images (H&E and Carstairs staining) of clearance organs from mice dosed with 10 mg/kg Lyo‐SP compared with mice dosed with vehicle (n = 5 per group). As evident from the images, no signs of off‐target thrombosis was found in the tissue beds of the clearance organs for Lyo‐SP‐dosed mice (organ histology images of Lyo‐SP dosed mice looked similar to that for vehicle‐dosed mice). These results demonstrate the safety and efficacy of Lyo‐SP at therapeutic doses in vivo to reduce bleeding in a thrombocytopenic setting in mice.

## 3 Discussion

Donor‐independent storage‐stable easily portable therapeutic agents that recapitulate specific hemostatic mechanisms of platelets are clinically highly sought after as ‘bridge technologies’ for treating bleeding complications when biologic platelet products are not sufficiently available for transfusion [76, 77, 78, 79]. To this end, we have developed a ‘synthetic platelet’ (SP) technology that utilizes surface‐decoration of liposomal nanoparticles with a combination of three fully synthetic peptides that integratively render platelet‐mimetic adhesive and aggregatory functions for hemostatic clot formation. SP is manufactured as a sterile aqueous suspension of the peptide‐decorated liposomal nanocostructs, and we have previously reported the mechanistic validation of SP in vitro and in vivo. Building on this, with a vision to enhance the storage‐stability, portability, and on‐demand availability of SP both within hospitals as well as in emergency use outside hospitals, we investigated the advancement of SP into a lyophilized powder product (Lyo‐SP).

Lyophilization has emerged as an effective strategy for several therapeutics including liposomal drug formulations to enhance storage‐stability and portability. Lyophilization involves removal of water in the form of ice via sublimation under reduced pressure. For liposomal systems this process can induce lipid packing instabilities as well as ice crystal induced damages to the vesicle membrane, such that upon rehydration the vesicles can exhibit morphological defects (and cargo leakage for drug‐loaded liposomes). This has prompted the utilization of natural anhydrobiosis‐inspired unique *lyoprotectant* molecules in the formulations that impart vitrification and thermodynamic stability, resulting in the protection of liposome morphology and cargo. Carbohydrates like trehalose, sucrose, dextrose, HPβCD etc. have been identified as effective lyoprotectants and are currently used in several FDA‐approved parenteral therapeutic formulations [54, 80]. Drawing from this knowledge, we evaluated several different lyoprotectant molecules for Lyo‐SP and only HPβCD was able to preserve optimal post‐lyophilization and storage‐stable size criteria. Accordingly, we established a two‐stage lyophilization process to freeze‐dry SP utilizing HPβCD as the lyoprotectant. HPβCD is listed by FDA as an inert excipient and is currently used in a variety of approved therapeutic formulations as a parenteral solubilizing excipient with published regulatory safety benchmarks [81, 82]. The European Medicines Agency (EMA) has noted that amounts of approximately 250 mg/kg/day are safe in humans older than 2 years when administered intravenously for up to 21 days, with contraindication only in severe renal impairment (Document EMA/CHMP/495747/2013). Consistent with this, US regulatory documentation for *tecovirimat* also references the EMA position and discusses substantial HPβCD exposure (8 g of HPβCD per 200 mg tecovirimat/20mL solution) to be safe in clinical evaluation. For our current Lyo‐SP formulation, the HPβCD dose is quite low (10% w/v) and is well below the safety limits discussed in the above documents, especially considering that Lyo‐SP shows hemostatic potency at doses as low as 1 mg/kg. Therefore, the HPβCD concentration used in Lyo‐SP aligns well within the established safety thresholds, in anticipated clinical doses.

SP is a unique liposomal therapeutic system in that it does not contain an encapsulated drug cargo per se, but its therapeutic function (i.e. hemostatic activity) is rendered by the heteromultivalent combination of surface‐decorated platelet function‐mimicking biointeractive peptides (the APIs). Therefore, it was important not only to confirm that the size and morphological stability is maintained in the aqueous‐reconstituted Lyo‐SP, but also that its combination peptide‐mediated hemostatic bioactivities are conserved. Our in vitro studies established that Lyo‐SP has enhanced storage stability at various temperature conditions, can be aqueous‐reconstituted rapidly, and retains the physicochemical and morphological aspects of SP. The fact that Lyo‐SP showed storage‐stability at room temperature similar to when stored at 4°C for at least up to 12 months, presents the possibility of storage and utilization of Lyo‐SP not only in hospital settings where cold‐chain storage may be adequately available, but also in resource‐limited settings where cold‐storage capabilities may be limited, and this is a substantial logistical benefit. Additionally, the indication that Lyo‐SP remains morphologically stable at much higher temperatures (50°C) for up to at least 2 months, indicates its storage and application potential in extreme hot weather regions of the world without the need for cold‐chain. Future studies will continue to investigate the functional conservation of Lyo‐SP activity when stored at such extreme temperatures for long periods of time. In microfluidic and flow cytometry analysis, Lyo‐SP demonstrated functional conservation of the biointeractive ability of SP, in terms of platelet‐mimetic adhesion to VWF and collagen (via surface‐decorated VBP and CBP peptides) and co‐aggregation with activated platelets (via surface‐decorated FMP peptides), even with long‐term (12‐month) storage. The hemostatic properties of Lyo‐SP remained highly viable in a whole blood setting, which is important for a nanoparticle‐based therapeutic system that is envisioned to operate in the vascular compartment. Lyo‐SP did not show major biosafety concerns regarding hemolytic effects, activation effects on endothelial cells and neutrophils, and activation of complement C3 to C3a and C3b. It is important to note here that Lyo‐SP is envisioned to be used as a platelet‐mimetic hemostatic product in patients that either have an inherent hemostatic compromise (e.g., thrombocytopenia) or have the risk or incidence of significant hemorrhage (e.g., surgery or trauma). For patients that may have a systemic inflammation phenotype (e.g., sepsis, atherosclerosis etc.) Lyo‐SP may be contraindicated in future clinical use. Therefore, within the current scope of our studies, we have not focused on evaluating Lyo‐SP in an already pre‐inflamed vascular environment setting, but rather confirmed that Lyo‐SP itself does not induce inflammatory activation on otherwise healthy vascular cells.

Our in vivo studies in mouse thrombocytopenia model further demonstrated that at optimum doses Lyo‐SP is safe and can render significant hemostatic effect (reduction in bleeding). An important aspect to note here is that the hemostatic effect of Lyo‐SP in the current in vivo model showed dose‐response (increased hemostatic effect) in going from 0.1 mg/kg to 1 mg/kg, but further increasing the dose to 10 mg/kg did not show further enhancement in hemostatic effect. This indicates a possible saturation of therapeutic effect. We have previously observed this aspect for SP too [40]. The platelet‐mimetic hemostatic function of SP (and hence Lyo‐SP) are all surface‐mediated (via VBP, CBP and FMP peptides). The SP nanoparticle surface area is approximately 100 times smaller than a platelet, such that theoretically 100 SP particles can provide surface‐relevant functional equivalence of 1 platelet. Our previous studies with SP have shown that the hemostatic restoration effect of SP is rendered ideally in the 1:100 to 1:1000 theoretical range of platelet:particle ratio, but beyond that range the overdosing of particles can cause crowding around native platelets as well as self‐competition for particle binding interactions, such that the overall functional outcome can not only reach saturation limits but can even become slightly inhibited. Such aspects can get reflected in the in vivo dose response as observed in the current TCP mouse tail‐clip model, where going from dose of 0.1 mg/kg to 1 mg/kg there was enhanced hemostatic effect, but then further increasing dose to 10 mg/kg does not show further enhancement of hemostatic effect. Such dose response profiles may change in going from one species to another, as well as from one bleeding model to another, and these will be part of our continued research with Lyo‐SP to find therapeutically effective (min‐to‐max) dosing range as well as maximum tolerated dose (MTD). Another important aspect to note here is that the blood loss and bleeding time of Lyo‐SP‐treated thrombocytopenic (TC) animals did not get restored back to the levels of healthy (control) animals. However, this is not surprising because the aim of intravenous hemostatic agents is to reduce bleeding significantly compared to the ‘defect’ condition, rather than be able to restore it to the level of wild type control. In fact, even transfusing natural platelets in hemostatically impaired mice does not restore bleeding profiles all the way back to wild type levels and this is also observed clinically in humans [83, 84]. Compared to bleeding in untreated TC mice, Lyo‐SP‐treated mice showed significant reduction in bleeding and this is considered a successful demonstration of hemostatic efficacy. Comparative studies with non‐lyophilized SP showed similar reduction in bleeding in the tail‐clip model in TC mice, further confirming that the hemostatic effect of SP remains conserved in Lyo‐SP.

We also note that in parallel to the studies reported here on evaluating intravenously (IV) administered Lyo‐SP in the tail‐clip model in TC mice, in a separate collaborative study Lyo‐SP was also investigated to explore feasibility of intraosseous (IO) delivery administered post‐injury in a rat trauma model of liver laceration hemorrhage, simulating an emergency use setting. These studies were recently published, and the results demonstrated that Lyo‐SP could be administered IO in an acute injury treatment setting (relevant to civilian and military emergency framework) and such administration led to reduced hemorrhage and increased survival [85]. Interestingly, in these studies we saw indications of endothelial glycocalyx protection in Lyo‐SP‐treated rats (circulating Syndecan signal was reduced in Lyo‐SP‐treated rats). Endothelial protection has been reported previously when bleeding is hemostatically treated with platelet transfusion [86]. Lyo‐SP, being a platelet‐mimicking system, may have similar aspects of secondary endothelial‐protective properties beyond its direct primary hemostatic function. The mechanistic possibilities of such protective effects are part of our ongoing research and thus beyond the scope of the current manuscript.Therefore, altogether these results establish the feasibility of utilizing Lyo‐SP in prophylactic (e.g. prior to surgery) as well as emergency (e.g. after acute injury) settings to mitigate bleeding complications and potentially reduce mortality where applicable.

In summary, advancement of SP to Lyo‐SP and subsequent evaluation of Lyo‐SP represent several innovative aspects including: (1) The first‐in‐kind investigation of freeze‐drying a liposomal system that is surface‐decorated with the combination of three different biointeractive peptides and confirming functional preservation post‐lyophilization and in long‐term storage; (2) The only ‘synthetic platelet’ technology to be advanced to a lyophilized product, and (3) The only *lyophilized synthetic platelet* technology to be evaluated in vitro in human blood and in vivo in prophylactic and emergency models of bleeding. To further advance Lyo‐SP toward clinical application, future studies will need to be focused on establishing good manufacturing practice (GMP) based large‐scale manufacturability of Lyo‐SP, while ensuring that such scale‐up processes do not affect the reconstitution time, physicochemical stability, and bioactivity of the product. Additionally, future studies will focus on establishing the detailed pharmacokinetic and toxicity profiles of the GMP‐manufactured Lyo‐SP batches. Potential adverse effects that may occur at high doses of nanoparticle‐based therapeutics include transient effect on blood cells (e.g. hemolysis, neutropenia, thrombocytopenia etc.), immune activation (leukocytes and complement system) and microvascular thrombotic risks. Such analyses, will be critical in advancing Lyo‐SP toward investigational new drug (IND) application and approval from the FDA to initiate clinical trials. Future studies will also provide the opportunity to evaluate Lyo‐SP across a range of clinically relevant bleeding complications beyond thrombocytopenia and traumatic hemorrhage, including antiplatelet or anticoagulant drug induced clotting dysfunctions, congenital platelet disorders, traumatic brain injuries etc. such that its hemostatic potential can be established across a broad spectrum of bleeding pathologies. Beyond serving as a stand‐alone platelet‐mimetic hemostatic therapy, Lyo‐SP can also be combined with other blood components, both natural and biosynthetic, such as plasma and oxygen carriers, to potentially achieve a lyophilized storage‐stable biosynthetic whole blood technology that can alleviate the constant challenges of shelf‐life and timely availability associated with donor‐derived whole blood for transfusion applications. The promise of such combinatorial transfusion products is underscored by recent reports on the utilization of freeze‐dried plasma to treat trauma‐associated coagulopathies and the development of freeze‐dried hemoglobin‐based RBC mimetics [87, 88]. There also remains the future opportunity to utilize Lyo‐SP as a complementary hemostatic adjunct with donor‐derived platelet products such as cold‐stored or cryopreserved platelets once all of these technologies become established as clinical products. With this vision, the current studies have achieved a translationally important milestone to establish Lyo‐SP as a shelf‐stable portable on‐demand aqueous‐reconstitutable platelet‐mimetic hemostatic technology for therapeutic management of bleeding complications.

## 4 Materials and Methods

### 4.1 Materials

The VBP sequence TRYLRIHPQSWVHQI, the CBP sequence [GPO]_7_ and the FMP sequence cyclo‐CNPRGDY[‐OEt]RC were selected based on the rationale described in detail in previous reports [43, 44, 45, 46, 47]. For current studies, VBP, CBP and FMP conjugated to distearoyl‐phosphatidylethanolamine via 2,000 Da molecular weight poly‐ethylene glycol spacers (i.e. DSPE‐PEG_2K_‐FMP, DSPE‐PEG_2K_‐VBP, DSPE‐PEG_2K_‐CBP), were custom‐synthesized by Bachem AG (St. Helens, United Kingdom). Distearoylphosphatidylcholine (DSPC), DSPE‐mPEG_1K_, DSPE‐Cy5, and Cholesterol were purchased from Avanti Polar Lipids Inc. (Alabaster, AL, USA). Hydroxy‐propyl‐beta‐cyclodextrin (HPβCD, used as lyoprotectant), 2‐[4‐(2‐hydroxyethyl)piperazin‐1‐yl]ethanesulfonic acid (HEPES) buffer, von Willebrand Factor (Factor VIII free), and apyrase were purchased from Sigma Aldrich (Saint Louis, MO, USA). Tyrode's buffer was purchased from Boston BioProducts (Ashland, MA, USA). Prostaglandin was purchased from Cayman Chemical (Ann Arbor, MI, USA). Phycoerythrin (PE) labeled Anti‐human CD62P was from BioLegend (San Diego, CA, USA). TRAP‐6 was purchased from BioData (Horsham, PA, USA). ROTEM supplies were purchased from Werfen (San Diego, CA, USA). 48‐well high‐shear microfluidic plates were purchased from Cell Microsystems (Durham, CA, USA). Equine Collagen was purchased from Chronolog (Havertown, PA, USA). Calcein AM was purchased from Molecular Probes (Eugene, OR, USA). AlexaFluor 350 NHS ester, AlexaFluor 488 NHS ester, Phosphate Buffered Saline (PBS), Bovine Serum Albumin (BSA), SYTOX Green, VECTASHIELD Antifade Mounting Media with DAPI, DMEM/F‐12, chamber slides, and NHS‐Fluorescein were purchased from Thermo Fischer Scientific (Waltham, MA, USA). Sodium citrate was purchased from Ricca Chemicals (Arlington, TX, USA). For neutrophil isolation from whole blood, EasySep Neutrophil Isolation Kit was purchased from STEMCELL (Vancouver, BC, Canada). Primary Umbilical Vein Endothelial Cells, Normal, Human (HUVEC) was obtained from ATCC (ATCC PCS‐100‐010) and cell line was contamination‐free. For HUVEC culture, vascular cell basal medium, endothelial cell growth kit‐VEGF, Trypsin‐EDTA solution, Trypsin neutralizing solution, Dulbecco's phosphate buffered saline (D‐PBS), were all purchased from ATCC (Manassas, VA, USA). Recombinant human TNF alpha protein (active), was purchased from Abcam (Waltham, MA, USA). Polyclonal Rabbit Anti‐Human von Willebrand Factor antibody was purchased from Dako Omnis, Agilent (Santa Clara, CA, USA). VE‐Cadherin (D87F2) Rabbit monoclonal antibody #2500, was purchased from Cell Signaling Technology, (Danvers, MA, US). Goat anti‐Rabbit IgG (H+L) cross‐adsorbed secondary antibody, Alexa Fluor 555 and 4´,6‐diamidino‐2‐phenylindole (DAPI) were purchased from Invitrogen (ThermoFisher, Waltham, US). Alexa Fluor 647 Donkey anti‐rabbit IgG antibody was purchased from BioLegend (San Diego, CA, USA). Paraformaldehyde Sol 4% and Corning Costar Not Treated Multiple Well Plate were purchased from Sigma Aldrich (Saint Louis, MO, USA). Human Complement C3 and C3a des Arg ELISA Kits were purchased from Abcam (Waltham, MA, USA). For mouse studies, anti‐CD42b antibody was purchased from Emfret (Wurzburg, Germany). Total hemoglobin reagent was from Pointe Scientific (Canton, MI, USA). C57BL/6J male mice between 18‐28 grams body weight was purchased from Charles River Laboratories (Hollister, CA, USA).

### 4.2 Equipment

For Lyo‐SP manufacture, the Lyovapor L‐200 Pro Lyophilizer and Rotavapor R‐200 roto‐evaporator was obtained from Buchi (New Castle, DE, USA), and 10 mL barrel extruder was obtained from Evonik (Essen, Germany). For Dynamic Light Scattering (DLS) based size and zeta potential based charge analyses of Lyo‐SP, the Litesizer 500 instrument and Omega Zeta Potential Cuvettes (Model 225228) were obtained from Anton Paar (Vernon Hills, IL, USA). Synergy MX fluorescent plate reader was obtained from BioTek (Winooski, VT, USA). For cryo‐TEM analyses the Tecnai T12 Cryo‐TEM (FEI company, Hillsboro, OR, USA) was used in the Case Western Reserve University School of Medicine (SOM) microscopy core. BioFlux 200 microfluidic equipment and plates were obtained from Cell Microsystems (Durham, CA, USA). Microscopy experiments were performed on a Zeiss Axio Observer epifluorescence microscope from Carl Zeiss (Oberkochen, Germany) for assessment of Lyo‐SP effect on endothelial VWF secretion and on neutrophil activation, and on a Leica Stellaris 5 confocal microscope from Leica (Wetzlar, Germany)] for all BioFlux microfluidic studies. BD Accuri C6 Flow Cytometer was obtained from BD Biosciences (Mississauga, ON, Canada). T‐TAS instrumentation and platelet (PL) chips were obtained from Diapharma (West Chester, OH, USA). ROTEM Delta was from Werfen (San Diego, CA, USA). HemaVet 950 was from Drew Scientific (Miami Lakes, FL, USA).

### 4.3 Manufacturing of Lyo‐SP

SP was manufactured using a thin film rehydration and extrusion technique as described previously but with a process modification of incorporating the lyoprotectant HPβCD in HEPES buffer to enable Lyo‐SP generation [89]. Briefly, DSPC, cholesterol, DSPE‐PEG_2K_‐VBP, DSPE‐PEG_2K_‐CBP, DSPE‐PEG_2K_‐FMP and DSPE‐mPEG_1K_ were mixed in 1:1 chloroform:methanol. Solvent was removed via rotary evaporation, and the thin lipid film was rehydrated using 2 mg/mL HEPES + 10% w/v HPβCD buffer (pH 7.0) at a final lipid concentration of 6 mg/mL. The lipid suspension was subjected to 10 freeze/thaw cycles and subsequent extrusion through 200 and 100nm pore diameter polycarbonate membrane using a pneumatic extruder (Evonik, Essen, Germany) to create unilamellar SP vesicles in suspension. The liquid SP samples were shell frozen in liquid nitrogen for 5 min and subjected to lyophilization by setting at a temperature of −45°C and a pressure vacuum of 0.2 mTorr for 960 min on the lyophilizer. Once lyophilization was initiated, the temperature was allowed to reach 0°C via heat transfer over the course of 60 min. Following the lyophilization cycle, the lyophilizer was nitrogen flushed and the vials were stoppered and crimped for storage until further use in the various experiments.

### 4.4 Characterization of Particle Size Charge Morphology and Storage‐stability of Lyo‐SP

All size and surface charge testing of SP and reconstituted Lyo‐SP were carried out with sample lipid concentration at 0.1 mg/mL, refractive index of 1.34, and viscosity of 0.00099 Pa(s). Mean intensity diameter (z‐average), poly‐dispersity index (PDI), and zeta potential were measured in at least triplicate for each sample. The storage stability of Lyo‐SP, reflected by size distribution analysis with DLS measurement, was evaluated under RT (∼25°C) and 4°C up to 12 months, as well as extreme low (−20°C) and high (50°C) temperatures up to 2 months. For morphology characterization with cryo‐TEM, fresh‐made SP or reconstituted Lyo‐SP samples were prepared at 0.5 mg/mL and adsorbed onto a glow‐discharged carbon coated copper grid for 1 min. The grid was blotted with filter paper and plunged into liquid ethane. Images were acquired between 9,000–30,000x magnification to characterize the nanoparticle sizes using ImageJ software.

### 4.5 Evaluation of SP and Lyo‐SP Binding to Platelets by Flow Cytometry

Human blood was drawn via venipuncture into 3 mL vacutainer tubes containing 3.2% sodium citrate anticoagulant, by a certified phlebotomist from NSAID‐refraining healthy donors, utilizing IRB‐approved protocol (IRB No. 20‐HAIM‐101 for Haima, IRB No. STUDY20191092 for Case Western). Platelet‐rich plasma (PRP) was obtained from this blood by centrifugation at 150 x g for 15 min. PRP was then diluted in a 1:1 (v/v) ratio with Tyrode's buffer. Next, 3 µL of 0.03 units/mL apyrase was added per mL of total volume, and the mixture was centrifuged at 100 x g for 15 min at 25°C. The supernatant was removed, and 3 µL of 1 µg/mL prostacyclin was added per mL of total volume. The mixture was centrifuged at 600 x g for 15 min at 25°C. The supernatant was removed, and the platelet pellet was resuspended in 1 mL of Tyrode's buffer and allowed to equilibrate on the benchtop for 20–30 min prior to testing. Platelets were incubated with 5 µL of SP or reconstituted Lyo‐SP at 1:500 platelets‐to‐particle ratio for 20 min at 25°C in the absence or presence of 5 µM of TRAP‐6. 5 µL of 100 µg/mL PE‐labeled anti‐human CD62P was added to stain activated platelets. Samples were transferred to FACS tubes through cell strainer cap and analyzed on a Flow Cytometer at 50,000 counts per sample, with platelet populations gating using side scatter (SSC) and forward scatter (FSC). Data shown in the manuscript was collected for the same batch of SP (and Lyo‐SP thereform) tested on 3 biological replicates (three different blood donors from whom platelets were isolated) per test condition.

### 4.6 BioFlux Microfluidic Characterization of Lyo‐SP Function and Hemostatic Effects

In the BioFlux microfluidic setup, microchannels are coated with hemostasis‐relevant proteins (e.g. collagen), test fluid (e.g. SP or Lyo‐SP suspension, plasma, blood, etc.) is flowed through the channel at controlled shear conditions using a pneumatic flow pump, and the processes within the channels are imaged in real‐time using fluorescence microscopy as depicted in the schematics of Figures S6 and S11 [65, 90]. To this end, microfluidic channels were first coated by flowing 50 µL of 100 µg/mL equine fibrillar Type I collagen solution at 30 dyn/cm^2^ for 30 s, then stopping the flow to allow the collagen to incubate at 37°C for 1 h. Next, 600 µL of 1% BSA was flowed through the collagen‐coated channel for 10 min at 30 dyn/cm^2^ to prevent nonspecific binding in subsequent experiments. The collagen coating was confirmed by labeling with 50 µL of 100 µg/mL Alexa Flour 350 NHS ester solution flowed through the channel at 30 dyn/cm^2^ for 30 s, then stopping the flow to allow the dye to incubate with collagen at 37°C for 15 min, then removing the dye solution by washing the channel with PBS flowed at 30 dyn/cm^2^ for 5 min, and finally imaging the fluorescently‐labeled (indigo blue) collagen fibers (example image in Figure S7A) to confirm the collagen coating. The ability of VWF to bind to this collagen‐coated surface was then tested, by first fluorescently labeling the VWF by adding Alexa Flour 488 NHS ester to 100 µL of 1 mg/mL VWF solution, incubating for 4 h on ice, removing the unlabeled dye via dialysis overnight, and then flowing 1 mL of the resultant fluorescent VWF over the collagen‐coated microfluidic channel at 60 dyn/cm^2^ for 10 min with real‐time imaging (example image Figure S7B). To study whether Lyo‐SP retains the platelet‐mimetic ability of SP to adhere to collagen in the presence of soluble VWF under flow, the collagen‐coated channels were prepared as above but without staining the collagen, and Cy5‐labeled (red fluorescent) SP or Lyo‐SP nanoparticles (40 µg/ml) along with 5 µg/mL AF488‐labeled VWF in PBS was flowed over the collagen at 60 dyn/cm^2^ for 10 min, and particle adhesion was imaged in real time under inverted fluorescence microscope. Next, microfluidic studies were performed to compare the ability of SP vs. Lyo‐SP to augment platelet recruitment and aggregation on collagen‐coated microchannels in the presence of soluble VWF in a platelet‐depleted (thrombocytopenic) setting, under high shear flow. Thrombocytopenia condition is an appropriate and clinically relevant test bed to study hemostatic effect of SP and Lyo‐SP, since platelet depletion results in compromised hemostatic outcomes and this has been clinically associated with various bleeding complications [91]. For this, human whole blood was centrifuged at 150g for 15 min at 25°C to obtain platelet rich plasma (PRP) and the PRP was further centrifuged at 13000g for 5 min at 25°C to obtain platelet‐free plasma (PFP). PRP platelet count was confirmed using a Coulter Counter and adjusted to 200,000/µL for the ‘normal’ condition. Additionally, PRP was diluted with PFP to get a platelet count of 20,000 platelets/µL for the thrombocytopenic plasma (TCP) condition. Prior to microfluidic experiments, PRP and TCP were incubated with Calcein AM for 20 min at 37°C to fluorescently label (green) platelets [92]. In each microfluidic experiment, 1.6% (v/v) 10µg/µL of Factor VIII‐free VWF was introduced as a soluble factor, to avoid donor‐to‐donor variability in native plasma VWF amount. SP or reconstituted Lyo‐SP was added to TCP at a platelet‐to‐particle ratio of 1:1000. PRP or TCP or ‘TCP + SP’ or ‘TCP + Lyo‐SP’ samples were allowed to flow over the collagen‐coated channels at 60 dyn/cm^2^ for 10 min with imaging under an inverted fluorescence microscope to monitor the interaction of green‐fluorescent platelets with red‐fluorescent SP or Lyo‐SP. The resultant videos were analyzed using Image J (NIH) and customized MATLAB code to determine platelet accumulation kinetics and platelet‐particle colocalization. Additional microfluidic studies were carried out to confirm that Lyo‐SP retains hemostatic bioactivity under a whole blood (WB) environment. Here, citrated whole blood obtained from a healthy donor was first centrifuged (150 g, 15 min, 25°C) to obtain the RBC fraction and PRP. The RBC fraction was washed three times at RBC:PBS 1:1 at 500g for 5 min. The PRP was further centrifuged (13000 g, 5 min, 25°C) to obtain PFP. The washed RBC fraction was then combined back with a reduced volume of PRP and a higher volume of PFP to maintain 40% ± 5% hematocrit, and either healthy (200k/µL ± 25k/µL) or thrombocytopenic (20k/µL ± 5k/µL) platelet count. Reconstituted WB samples (healthy or thrombocytopenic) were incubated with 1 µg/mL Calcein AM to fluorescently label platelets and subjected to BioFlux microfluidic studies on collagen‐coated channels as before, at 60 dyn/cm^2^. The thrombocytopenic WB (TC‐WB) was treated with Lyo‐SP (platelet‐to‐particle ratio 1:1000), and ‘TC‐WB + Lyo‐SP’ samples under flow were imaged with confocal fluorescence microscopy to quantify the platelet accumulation.

### 4.7 Clotting Kinetics Evaluation With Lyo‐SP in T‐TAS

The Total Thrombus Analysis System (T‐TAS) is a clinically relevant microfluidic system that uses whole blood samples to flow through microfluidic capillary channels coated with collagen (for platelet chip or PL‐chip), to enable the temporal evaluation of platelet‐mediated clot formation [67]. The instrument setup is shown in Figure S14A. The assay is performed under arterial flow conditions (flow of 18 µL/min, shear rate 1500 s^−1^) using benzylsulfonyl‐D‐Arg‐Pro‐4‐amidinobenzylamide (BAPA)‐anticoagulated whole blood samples. BAPA inhibits thrombin and Factor Xa, thereby blocking the contribution of the coagulation cascade and enabling the PL‐Chip assay to specifically measure only the platelet contribution to clot formation. The growing platelet thrombus causes occlusion of the microcapillary channels, causing an increase in the flow pressure within the assay chip that is continuously monitored by a pressure sensor. Results are calculated automatically for 10 min or until the pressure reading reaches 60 kPa above the baseline pressure, whichever occurs first. The characteristic parameters measured, namely Occlusion Start Time (OST, reflective of the initiation of clot formation), Occlusion Time (OT, reflective of time for complete channel occlusion due to clot) and area‐under‐curve (AUC, reflective of overall clot formation), are shown in Figure S14B. The effect of Lyo‐SP on clot kinetics in T‐TAS PL‐Chip was evaluated in thrombocytopenic whole blood (TC‐WB), and healthy whole blood (WB, no platelet depletion) was used as the positive control. 5 µL of reconstituted Lyo‐SP samples were added to 1000 µL of TC‐WB to maintain a platelet:particle ratio of 1:75. 320 µL of this mixture was added to the PL Chip and analyzed by T‐TAS. Occlusion Start Time (OST, reflective of clot initiation capability), Occlusion Time (OT, reflective of clot growth), and area under curve (AUC, reflective of overall clotting capability), were recorded for WB vs. TC‐WB vs. Lyo‐SP treated TC‐WB samples.

### 4.8 Clotting Kinetics Evaluation With Lyo‐SP in ROTEM

Rotational Thromboelastometry (ROTEM) is a clinically relevant viscoelastometric assay that measures the clotting kinetics of whole blood [68]. The experiment involves placing blood sample in a cuvette and lowering an oscillating pin into this cuvette such that the pin oscillation is impeded by coagulating blood, and this mechanical impedance is optically detected to obtain specific parameters reflective of clot kinetics (experimental set‐up shown in Figure S15A). The characteristic measurement profile and associated parameters relevant to the current studies are shown in Figure S15B where Clot Formation Time (CFT) is reflective of initial clot kinetics, alpha angle (α) is reflective of clot growth rate and amplitude at 10 min after clotting time (A10) is reflective of the clot stability as it grows. Lyo‐SP was evaluated using the Non‐activated Thromboelastometry (NATEM) mode in ROTEM, where coagulation of recalcified citrated blood occurs without the addition of any specific reagent to induce activation of specific coagulation factors. In NATEM, the presence of calcium ions activate platelets (including induction of procoagulant platelets) and subsequent activation of coagulation factors to form thrombin (and hence fibrin), without any additional direct activation of coagulation factors. Therefore, we have found this modality to be more sensitive specifically to the count and function of platelets, especially regarding the kinetics of clot formation in healthy versus thrombocytopenic conditions. Since the ability of SP is to compensate for and rescue platelet activity (adhesion and aggregation) in thrombocytopenic setting (as demonstrated by our BioFlux studies), we hypothesized that this rescue effect will also be captured in the NATEM mode in ROTEM. Therefore, the effect of Lyo‐SP on clot kinetics was evaluated in TC‐WB, and healthy WB (no platelet depletion) was used as the positive control. For this, 1.5 µL of reconstituted Lyo‐SP samples were added to 300 µL of TC‐WB to maintain a platelet: particle ratio of 1:100 and analyzed by ROTEM in NATEM mode. Specific kinetic parameters, namely, CFT (in seconds), A10 (in mm) and α (in degrees) were recorded for WB vs. TC‐WB vs. Lyo‐SP treated TC‐WB.

### 4.9 Evaluation of Lyo‐SP Effect on Endothelial Cells

Human umbilical vein endothelial cells (HUVECs) in monolayer culture are commonly used to test endothelial activation and toxicity risks associated with nanoparticles, and therefore this approach was adapted to study the effect of Lyo‐SP on HUVECs [93]. Tumor Necrosis Factor alpha (TNF‐α) is a potent activator of ECs and was used as a positive control for these studies [94]. For analysis of endothelial status, two complimentary analyses were performed, namely, analysis of VE‐Cadherin as a marker of endothelial barrier function disruption and analysis of VWF secretion as a marker of endothelial activation [94, 95]. In a typical experiment for VE‐Cadherin analysis, HUVEC cells (1.2 × 10^5^ cells/mL) were seeded two days prior to the experiment in a 24‐well plate to reach 80%–90% confluency. Once confluent, the HUVECs were incubated with aqueous‐reconstituted non‐fluorescent Lyo‐SP (40 µg/mL) for 24 h prior to fluorescence staining of the VE‐Cadherin on the cells. Cells without TNF‐α activation (resting endothelial cells) served as the negative control, while cells incubated for 4 h with TNF‐α (40 ng/mL) were used as the positive control. Post‐incubation, cells were washed with 500 µL DPBS and fixed with 4% paraformaldehyde for 15 min at RT (300 µL added per well of the 24‐well plate). After fixation, the cells were rinsed 2‐3 times with DPBS. Cells were blocked with 2% BSA for 1h at RT. 10 min before the end of the blocking step, the primary antibody was prepared by diluting VE‐Cadherin antibody at 1:400 in 1% BSA. Following blocking, the cells were incubated with the primary antibody solution overnight at 4°C. Cells were then washed 2–3 times with DPBS. The secondary antibody (AF647‐conjugated donkey anti‐rabbit IgG, 406414, BioLegend) and DAPI (1µgm/mL) were diluted in 1% BSA, and incubated with the cells overnight at 4°C. Post‐incubation, the cells were gently washed and imaged using a confocal fluorescence microscope. For activated endothelium, the inter‐cell barrier is disrupted and VE‐Cadherin is delocalized from the membrance barrier into the cytosol, and this phenomen was used to assess barrier disruption. To quantify the spatial location of VE‐cadherin, we used stratified random sampling to select representative cells in ImageJ, obtained a profile plot of the fluorescence intensity along a segment spanning each cell, then represented the spatial distribution of VE‐cadherin as a ratio of fluorescence at the membrane to that of the cytoplasm. In a typical experiment to analyze VWF secretion, HUVEC cells (1.2 × 10^5^ cells/mL) were seeded two days prior to the experiment in a 24‐well plate to reach 80%–90% confluency. The confluent HUVECs were incubated with aqueous‐reconstituted Lyo‐SP (40 µg/mL) for 24 h prior to staining. Cells without TNF‐α activation (resting endothelial cells) served as the negative control, while cells incubated for 4 h with TNF‐α (40 ng/mL) were used as the positive control. Post‐incubation, the cells were washed with 500 µL DPBS and blocked with 2% BSA in PBS for 30 min. Poly rabbit anti‐human VWF antibody, used directly without further dilution, was added (300 µL added per well of the 24‐well plate) and incubated for 1h at RT. The cells were then washed 1–2 times with DPBS. Thereafter, the cells were fixed with 4% PFA for 15 min at RT (300 µL added per well of the 24‐well plate). After fixation, cells were incubated with secondary antibody (goat anti‐rabbit IgG Alexa Fluor 555, A‐21428, Invitrogen) and DAPI (1µgm/mL) diluted in 1% BSA for 1h at RT in the dark. The cells were then washed 2–3 times with DPBS and finally imaged using an epifluorescence microscope. VWF fluorescence intensity was quantified using ImageJ (NIH).

### 4.10 Evaluation of Lyo‐SP Effect on Neutrophils

Neutrophils are the most abundant innate immune cells in circulation, and they are involved in protection against pathogens. The defense mechanism of neutrophils against pathogens involves neutrophil activation and a complex cascade of signals that ultimately result in neutrophil membrane permeability and extrusion of the neutrophil DNA outside the cell, a process termed neutrophil extracellular trap (NET) formation or NET‐osis [96]. While NET‐osis is an obligatory and vital immune function against pathogens, aberrant neutrophil activation and NET formation can lead to harmful thromboinflammatory pathology. Therefore, any risk of Lyo‐SP toward neutrophil activation and NET‐osis was tested in vitro using isolated human neutrophils. Phorbol Myristate Acetate (PMA) was used as a positive control since it is a potent agent for neutrophil activation and NET‐osis, and SYTOX Green was used to stain permeabilized activated neutrophils and NETs [61, 62]. In a typical experiment, neutrophils were isolated from whole blood by immunomagnetic separation, resuspended in DMEM/F‐12 containing 1% bovine serum albumin, 2 mM CaCl_2_ and 2 mM MgCl_2_ at a concentration of 5 x 10^5^ cells/mL, and seeded on fibrinogen‐coated slides. Neutrophils were stimulated for 1 h with 100nM PMA (positive control), 40µg/mL Lyo‐SP, or normal saline (negative control). Post‐incubation, neutrophils were stained with 167nM SYTOX Green for 15 min, washed with PBS, fixed with 4% paraformaldehyde, and mounted with VACTASHIELD mounting media containing DAPI. Slides were imaged using a confocal fluorerscence microscope and quantified on ImageJ (NIH). The experiment was performed with neutrophils isolated from the blood of 3 different donors.

### 4.11 Evaluation of Lyo‐SP Effect on Complement C3 Activation

In the context of assessing immune risks associated with nanoparticles, activation of complement factor C3 to C3a is considered a risk factor for potential anaphylactic toxicity [73]. Therefore, the risk of C3 activation by Lyo‐SP was assessed in human plasma using appropriate ELISA assay. Lipopolysachharide (LPS) is a potent activator of C3 to C3a and was thus used as a positive control. Aqueous‐reconstututed Lyo‐SP (40 µg/mL) was incubated with healthy human plasma for 1 h, then samples were assayed using the appropriate ELISA kit. The ratio of C3 and C3a des Arg (a metabolite of activated C3a) was used to analyze and quantify complement activation.

### 4.12 Evaluation of Lyo‐SP in Tail‐Clip Bleeding Model in Thrombocytopenic (TCP) Mice

The thrombocytopenia model in mice was adapted from established model standardization reports [74, 75]. All studies involving animals strictly followed the Case Western Reserve University Institutional Animal Care and Use Committee (IACUC: 2021‐0034). Genetically identical strains of male C57 black 6 (C57/BL6), mice were used. Intraperitoneal injection of Ketamine (150 mg/kg) and Xylazine (10 mg/kg) were used to anesthetize the mice. A 0.1 mL jugular blood draw was conducted for baseline platelet counting via HemaVet 950. Anti‐CD42b, an anti‐GPIbα antibody, was administered intraperitoneally at a dose of 0.2 µg/g to induce TCP. Eighteen (18) h after antibody injection, mice were re‐anesthetized with Ketamine (150 mg/kg) and Xylazine (10 mg/kg) via intraperitoneal injection. Retroorbital blood draws were conducted to assess post antibody platelet counts and verification of TCP on HemaVet 950. Following confirmation of TCP at 500 platelets/nL or lower, 150 µL of reconstituted Lyo‐SP was administered via retroorbital injection at doses of 0.1, 1, and 10 mg/kg total lipids (n = 7 per group). Fifteen minutes after treatment, a tail transection was performed 1 mm from the tip with a surgical blade, and the tail was immersed in 1200 µL of saline at 37°C. The time for bleeding to stop was measured and capped at 20 min. To assess blood loss volume, hemoglobin content in the shed blood was measured. Specifically, 150 µL of total hemoglobin reagent was mixed with 50 µL of sample (or standard) and incubated for 3 min prior to absorbance reading using a plate reader. Samples were run in triplicate. Blood loss (in µL) was calculated by subtracting the background absorbance reading from the blood sample absorbance reading, normalized by the hemoglobin standard curve.

## 5 Statistics

For all analyses GraphPad (version 10.3.1) (San Diego, CA, USA) was used. All experiments were performed in at least triplicate in order to obtain mean and standard deviation. The standard deviation (σ), along with Z of 1.96 (95% confidence interval), and the margin of 25% (E) was used to calculate the appropriate sample sizes (n) using Cochrane's power analysis. Percent difference values were calculated to demonstrate changes in physiochemical metrics when comparing SP vs. Lyo‐SP groups. Statistical significance of functional metrics compared to controls was determined using a one‐way or two‐way ANOVA, as appropriate, followed by Tukey's post hoc test for multiple comparisons, using GraphPad Prism software. Bleeding time and blood loss were analyzed using a one‐way ANOVA with a Tukey's multiple comparisons test and normalized to the weight of the mice tested.

## Author Contributions

A.S.G., C.L.P. and M.B. contributed to the conceptualization, experimental supervision and manuscript supervision. A.S.G., C.L.P., U.D.S.S., D.D, S.M.S., P.C.S., M.D.N. contributed to manuscript compilation and editing. U.D.S.S., S.D., B.T., E.G., E.Q., A. Dornback, A. Ditto contributed to SP and Lyo‐SP manufacture. U.D.S.S., E.G., R.A., E.Q., S.S., A. Ditto, B.C.P. contributed to SP and Lyo‐SP physico‐chemical characterization. E.G., B.C.P., R.A., E.Q. contributed to SP vs. Lyo‐SP ligand function characterization. D.D. and S.S. contributed to SP vs. Lyo‐SP analysis using BioFlux microfluidics in human plasma. D.D., K.A., R.A., U.D.S.S. contributed to Lyo‐SP analysis in human blood. S.Rohiwal and N.F.L. contributed to Lyo‐SP analysis on HUVEC. S. Raghunathan contributed to Lyo‐SP analysis on Neutrophils. N.F.L. contributed to Lyo‐SP analysis on Complement C3. U.D.S.S., E.G. and S.S. contributed to Lyo‐SP evaluation in mouse thrombocytopenia model. U.D.D.S. and B.C.P. compiled Biorender schematic. All authors have read and approved the manuscript for submission.

## Funding

United States Department of Defense grant W81XWH‐20‐1‐0628 to A.S.G. and W81XWH‐17‐2‐0064 to Haima Therapeutics.

## Conflicts of Interest

A.S.G. is an inventor and co‐founder of Haima Therapeutics LLC, a biotechnology start‐up company focused on the research and pre‐clinical development of bioinspired hemostatic technologies. A.S.G. serves as the Chief Technology Officer of Haima. C.L.P. is a co‐inventor and co‐founder of Haima and serves as the Chief Operations Officer of the company. M.B. serves as the Chief Executive Officer of Haima. U.D.S.S serves as a senior scientist at Haima. B.T., E.G., E.Q., and K.A. are staff scientists at Haima. S.D., S.S., A.D., and A.D, have served as scientists at Haima. R.A. has served as a research intern at Haima. M.D.N. serves as the Chief Medical Officer of Haima. M.D.N. and P.C.S. serve in the scientific advisory board of Haima. A.S.G. is a co‐inventor on patents US 9107845, US 9636383, US 10426820, US 10434149, licensed to Haima.

## Supporting information




**Supporting File 1**: advs75336‐sup‐0001‐SuppMat.docx.

**Supporting File 2**: advs75336‐sup‐0002‐MovieS1.mp4.

**Supporting File 3**: advs75336‐sup‐0003‐MovieS2.mp4.

**Supporting File 4**: advs75336‐sup‐0004‐MovieS3.mp4.

**Supporting File 5**: advs75336‐sup‐0005‐MovieS4.mp4.

**Supporting File 6**: advs75336‐sup‐0006‐MovieS5.mp4.

**Supporting File 7**: advs75336‐sup‐0007‐MovieS6.mp4.

**Supporting File 8**: advs75336‐sup‐0008‐MovieS7.mp4.

**Supporting File 9**: advs75336‐sup‐0009‐MovieS8.mp4.

**Supporting File 10**: advs75336‐sup‐0010‐MovieS9.mp4.

**Supporting File 11**: advs75336‐sup‐0011‐MovieS10.mp4.

**Supporting File 12**: advs75336‐sup‐0012‐MovieS11.mp4.

## Data Availability

The data that support the findings of this study are available from the corresponding author upon reasonable request.

## References

[1] M. Hoffman , “Monrow 3^rd^, D.M. A Cell‐based Model of Hemostasis,” Thromb Haemost 85 (2001): 958–965. 11434702

[2] H. H. Versteeg , J. W. M. Heemskerk , M. Levi , and P. H. Reitsma , “New Fundamentals in Hemostasis,” Physiological Reviews 93 (2013): 327–358. 23303912 10.1152/physrev.00016.2011

[3] M. Cattaneo , “Inherited Platelet‐based Bleeding Disorders,” Journal of Thrombosis and Haemostasis 1 (2003): 1628–1636. 12871299 10.1046/j.1538-7836.2003.00266.x

[4] P. J. Vinholt , “The Role of Platelets in Bleeding in Patients With Thrombocytopenia and Hematological Disease,” Clinical Chemistry and Laboratory Medicine (CCLM) 57 (2019): 1808–1817. 31465290 10.1515/cclm-2019-0380

[5] P. H. Sloos , P. Vulliamy , C. van t′ Veer , et al., “Platelet Dysfunction After Trauma: From Mechanisms to Targeted Treatment,” Transfusion 62 (2022): S281–S300. 35748694 10.1111/trf.16971 PMC9546174

[6] A. Kumar , R. Mhaskar , B. J. Grossman , et al., “AABB Transfusion Guideline Panel. AABB Platelet Transfusion Guidelines Panel, Platelet Transfusion: A Systematic Review of the Clinical Evidence,” Transfusion 55 (2015): 1116–1127. 25387589 10.1111/trf.12943

[7] E. W. Etchill , S. P. Myers , J. S. Raval , A. Hassoune , A. Sen Gupta , and M. D. Neal , “Platelet transfusion in critical care and surgery: Evidence‐based review of contemporary practice and future directions,” Shock 47 (2017): 537–549. 27849676 10.1097/SHK.0000000000000794

[8] A. Newland , R. Bentley , A. Jakubowska , et al., “A Systematic Literature Review on the Use of Platelet Transfusions in Patients With Thrombocytopenia,” Hematology (Amsterdam, Netherlands) 24 (2019): 679–719. 31581933 10.1080/16078454.2019.1662200

[9] J. C. Cardenas , X. Zhang , E. E. Fox , et al., “Platelet transfusions improve hemostasis and survival in a substudy of the prospective, randomized PROPPR trial,” Blood Advances 2 (2018): 1696–1704. 30030268 10.1182/bloodadvances.2018017699 PMC6058234

[10] T. Thiele and A. Greinacher , “Platelet Transfusion in Perioperative Medicine,” Seminars in Thrombosis and Hemostasis 46 (2020): 50–61. 31830766 10.1055/s-0039-1697951

[11] E. K. Storch , B. S. Custer , M. R. Jacobs , J. E. Menitove , and P. D. Mintz , “Review of Current Transfusion Therapy and Blood Banking Practices,” Blood Reviews 38 (2019): 100593. 31405535 10.1016/j.blre.2019.100593

[12] G. Vit , H. Klüter , and P. Wuchter , “Platelet Storage and Functional Integrity,” Journal of Laboratory Medicine 44 (2020): 285–293.

[13] C. Humbrecht , D. Kientz , and C. Gachet , “Platelet Transfusion: Current Challenges,” Transfusion Clinique et Biologique 25 (2018): 151–164. 30037501 10.1016/j.tracli.2018.06.004

[14] P. S. Alcaina , “<p>Platelet Transfusion: And Update on Challenges and Outcomes</p>,” Journal of Blood Medicine 11 (2020): 19–26. 32158298 10.2147/JBM.S234374 PMC6986537

[15] J. H. Levy , M. D. Neal , and J. H. Herman , “Bacterial Contamination of Platelets for Transfusion: Strategies for Prevention,” Critical Care 22 (2018): 271. 30367640 10.1186/s13054-018-2212-9 PMC6204059

[16] P. C. Spinella , J. Dunne , G. J. Beilman , et al., “Constant Challenges and Evolution of US Military Transfusion Medicine and Blood Operations in Combat,” Transfusion 52 (2012): 1146–1153. 22575063 10.1111/j.1537-2995.2012.03594.x

[17] M. P. Lambert , S. K. Sullivan , R. Fuentes , D. L. French , and M. Poncz , “Challenges and promises for the development of donor‐independent platelet transfusions,” Blood 121 (2013): 3319–3324. 23321255 10.1182/blood-2012-09-455428 PMC3976218

[18] N. Roberts , S. James , M. Delaney , and C. Fitzmaurice , “The Global Need and Availability of Blood Products: A Modelling Study,” The Lancet Haematology 6 (2019): e606–e615. 31631023 10.1016/S2352-3026(19)30200-5

[19] J. R. Stubbs , B. H. Shaz , R. R. Vassallo , and J. D. Roback , “Expanding the Platelet Inventory to Mitigate the Impact of Severe Shortages,” Hematology (Amsterdam, Netherlands) 2022 (2022): 424–429. 10.1182/hematology.2022000379 PMC9821291 36485081

[20] A. M. P. Brito and M. Schreiber , “Prehospital Resuscitation,” Trauma Surgery & Acute Care Open 6 (2021): 000729. 10.1136/tsaco-2021-000729 PMC8112406 34041365

[21] R. M. Schaefer , E. A. Bank , J. R. Krohmer , et al., “Removing the Barriers to Prehospital Blood: A Roadmap to Success,” Journal of Trauma and Acute Care Surgery 97 (2024): S138–S144. 38689393 10.1097/TA.0000000000004378

[22] A. Magron , J. Laugier , P. Provost , and E. Boilard , “Pathogen Reduction Technologies: The Pros and Cons for Platelet Transfusion,” Platelets 29 (2018): 2–8. 28523956 10.1080/09537104.2017.1306046

[23] K. M. Hoffmeister , E. C. Joseffson , N. A. Isaac , H. Clausen , J. H. Hartwig , and T. P. Stossel , “Glycosylation Restores Survival of Chilled Blood Platelets,” Science 301 (2003): 1531–1534. 12970565 10.1126/science.1085322

[24] K. M. Reddoch‐Cardenas , J. A. Bynum , M. A. Meledeo , et al., “Cold‐stored platelets: A product With function optimized for hemorrhage control,” Transfusion and Apheresis Science 58 (2019): 16–22. 30704925 10.1016/j.transci.2018.12.012

[25] T. M. Getz , “Physiology of Cold‐stored Platelets,” Transfusion and Apheresis Science 58 (2019): 12–15. 30639086 10.1016/j.transci.2018.12.011

[26] D. C. Marks and L. Johnson , “Assays for Phenotypic and Functional Characterization of Cryopreserved Platelets,” Platelets 30 (2019): 48–55. 30252562 10.1080/09537104.2018.1514108

[27] D. J. B. Kleinveld , P. H. Sloos , F. Noorman , et al., “The use of cryopreserved platelets in a trauma‐induced hemorrhage model,” Transfusion 60 (2020): 2079–2089. 32592423 10.1111/trf.15937 PMC7540664

[28] A. P. Cap and J. G. Perkins , “Lyophilized Platelets: Challenges and Opportunities,” Journal of Trauma 70 (2011): S59–S60. 21841577 10.1097/TA.0b013e31821a606d

[29] J. A. Bynum , M. A. Meledeo , G. C. Peltier , et al., “Evaluation of a lyophilized platelet‐derived hemostatic product,” Transfusion 59 (2019): 1490–1498. 30980737 10.1111/trf.15167

[30] B. J. Kuhn , A. Swanson , A. S. Cherupalla , et al., “Mechanisms of Action of an Investigational New Freeze‐dried Platelet‐derived Hemostatic Product,” Journal of Thrombosis and Haemostasis 22 (2024): 686–699. 38072376 10.1016/j.jtha.2023.11.022

[31] J. L. Sperry , F. X. Guyette , R.‐R. BL , et al., “Early Cold Stored Platelet Transfusion Following Severe Injury,” Annals of Surgery 280 (2024): 212–221. 38708880 10.1097/SLA.0000000000006317 PMC11224567

[32] J. Leung , C. Strong , K. E. Badior , et al., “Genetically Engineered Transfusable Platelets Using mRNA Lipid Nanoparticles,” Science Advances 9 (2023): adi0508. 10.1126/sciadv.adi0508 PMC10691771 38039367

[33] J. N. Thon , L. Mazutis , S. Wu , et al., “Platelet bioreactor‐on‐a‐chip,” Blood 124 (2014): 1857–1867. 25606631 10.1182/blood-2014-05-574913 PMC4168343

[34] Y. Ito , S. Nakamura , N. Sugimoto , et al., “Turbulence Activates Platelet Biogenesis to Enable Clinical Scale Ex Vivo Production,” Cell 174 (2018): 636–648.e18. 30017246 10.1016/j.cell.2018.06.011

[35] L. Tozzi , P.‐A. Laurent , C. A. Di Buduo , et al., “Multi‐channel Silk Sponge Mimicking Bone Marrow Vascular Niche for Platelet Production,” Biomaterials 178 (2018): 122–133. 29920404 10.1016/j.biomaterials.2018.06.018 PMC6082392

[36] S. Mookerjee , H. R. Foster , A. K. Waller , and C. J. Ghevaert , “In Vitro‐derived Platelets: The Challenges We Will Have to Face to Assess Quality and Safety,” Platelets 31 (2020): 724–730. 32486997 10.1080/09537104.2020.1769051

[37] C. Modery‐Pawlowski , L. L. Tian , V. Pan , K. R. McCrae , S. Mitragotri , and A. Sen Gupta , “Approaches to Synthetic Platelet Analogs,” Biomaterials 34 (2013): 526–541. 23092864 10.1016/j.biomaterials.2012.09.074

[38] A. C. Brown , S. E. Stabenfeldt , B. Ahn , et al., “Ultrasoft Microgels Displaying Emergent Platelet‐Like Behaviours,” Nature Materials 13 (2014): 1108–1114. 25194701 10.1038/nmat4066 PMC4239187

[39] A. C. Anselmo , C. L. Modery‐Pawlowski , S. Menegatti , et al., “Platelet‐Like Nanoparticles: Mimicking Shape, Flexibility, and Surface Biology of Platelets To Target Vascular Injuries,” ACS Nano 8 (2014): 11243–11253. 25318048 10.1021/nn503732m PMC4246005

[40] U. D. S. Sekhon , K. Swingle , A. Girish , et al., “Platelet‐mimicking Procoagulant Nanoparticles Augment Hemostasis in Animal Models of Bleeding,” Science Translational Medicine 14 (2022): abb8975. 10.1126/scitranslmed.abb8975 PMC9179936 35080915

[41] N. F. Luc , N. Rohner , A. Girish , U. D. S. Sekhon , M. D. Neal , and A. Sen Gupta , “Bioinspired Artificial Platelets: Past, Present and Future,” Platelets 33 (2022): 35–47. 34455908 10.1080/09537104.2021.1967916 PMC8795470

[42] D. Disharoon , S. Rohiwal , S. Hernandez , et al., “Biosynthetic Blood Surrogates: Current Status and Future Opportunities,” Bioengineering & Translational Medicine 10 (2025): 70084. 10.1002/btm2.70084 PMC12617565 41244334

[43] M. Ravikumar , T. Wong , C. Modery , and A. Sen Gupta , “Peptide‐decorated Liposomes Promote Arrest and Aggregation of Activated Platelets Under Flow on Vascular Injury Relevant Protein Surfaces in Vitro,” Biomacromolecules 13 (2012): 1495–1502. 22468641 10.1021/bm300192t

[44] C. L. Modery‐Pawlowski , L. L. Tian , M. Ravikumar , T. L. Wong , and A. Sen Gupta , “In vitro and in vivo hemostatic capabilities of a functionally integrated platelet‐mimetic liposomal nanoconstruct,” Biomaterials 34 (2013): 3031–3041. 23357371 10.1016/j.biomaterials.2012.12.045

[45] G. Huang , Z. Zhou , R. Srinivasan , et al., “Affinity Manipulation of Surface‐conjugated RGD Peptide to Modulate Binding of Liposomes to Activated Platelets,” Biomaterials 29 (2008): 1676–1685. 18192005 10.1016/j.biomaterials.2007.12.015 PMC2278119

[46] M. Ravikumar , C. L. Modery , T. L. Wong , M. Dzuricky , and A. Sen Gupta , “Mimicking Adhesive Functionalities of Blood Platelets Using Ligand‐decorated Liposomes,” Bioconjugate Chemistry 23 (2012): 1266–1275. 22607514 10.1021/bc300086d

[47] H. Haji‐Valizadeh , C. L. Modery‐Pawlowski , and A. Sen Gupta , “A Factor VIII‐derived Peptide Enables von Willebrand Factor (VWF)‐binding of Artificial Platelet Nanoconstructs Without Interfering With VWF‐adhesion of Natural Platelets,” Nanoscale 6 (2014): 4765–4773. 24658160 10.1039/c3nr06400j PMC4300948

[48] M. Shukla , U. D. S. Sekhon , V. Betapudi , et al., “In vitro characterization of SynthoPlate™ (synthetic platelet) technology and its in vivo evaluation in severely thrombocytopenic mice,” Journal of Thrombosis and Haemostasis 15 (2017): 375–387. 27925685 10.1111/jth.13579 PMC5305617

[49] M. R. Dyer , D. Hickman , N. Luc , et al., “Intravenous Administration of Synthetic Platelets (SynthoPlate) in a Mouse Liver Injury Model of Uncontrolled Hemorrhage Improves Hemostasis,” Journal of Trauma and Acute Care Surgery 84 (2018): 917–923. 29538234 10.1097/TA.0000000000001893 PMC5970031

[50] D. A. Hickman , C. L. Pawlowski , A. Shevitz , et al., “Intravenous synthetic platelet (SynthoPlate) nanoconstructs reduce bleeding and improve ‘golden hour’ survival in a porcine model of traumatic arterial hemorrhage,” Scientific Reports 8 (2018): 3118. 29449604 10.1038/s41598-018-21384-z PMC5814434

[51] S. Roullet , N. Luc , J. Rayes , et al., “Efficacy of platelet‐inspired hemostatic nanoparticles on bleeding in Von Willebrand disease murine models,” Blood 141 (2023): 2891–2900. 36928925 10.1182/blood.2022018956 PMC10315625

[52] A. J. Srinivasan , Z. A. Secunda , R. I. Mota‐Alvidrez , et al., “Platelet‐inspired Synthetic Nanoparticles Improve Hemostasis and Hemodynamics in a Rabbit Model of Abdominal Hemorrhage,” Journal of Trauma and Acute Care Surgery 96 (2024): 101–108. 38057963 10.1097/TA.0000000000003938 PMC10746291

[53] C. Chen , D. Han , C. Cai , and X. Tang , “An Overview of Liposome Lyophilization and Its Future Potential,” Journal of Controlled Release 142 (2010): 299–311. 19874861 10.1016/j.jconrel.2009.10.024

[54] Y. Wang and D. W. Grainger , “Lyophilized Liposome‐based Parenteral Drug Development: Reviewing Complex Product Design Strategies and Current Regulatory Environments,” Advanced Drug Delivery Reviews 151‐152 (2019): 56–71. 10.1016/j.addr.2019.03.003 30898571

[55] E. Trenkenschuh and W. Friess , “Freeze‐drying of Nanoparticles: How to Overcome Colloidal Instability by Formulation and Process Optimization,” European Journal of Pharmaceutics and Biopharmaceutics 165 (2021): 345–360. 34052428 10.1016/j.ejpb.2021.05.024

[56] J. H. Crowe , J. F. Carpenter , and L. M. Crowe , “The Role of Vitrification in Anhydrobiosis,” Annual Review of Physiology 60 (1998): 73–103. 10.1146/annurev.physiol.60.1.73 9558455

[57] J. M. van den Hoven , J. M. Metselaar , G. Storm , J. H. Beijnen , and B. Nuijen , “Cyclodextrin as membrane protectant in spray‐drying and freeze‐drying of PEGylated liposomes,” International Journal of Pharmaceutics 438 (2012): 209–216. 22960501 10.1016/j.ijpharm.2012.08.046

[58] N. M. Payton , M. F. Wempe , Y. Xu , and T. J. Anchordoquy , “Long‐Term Storage of Lyophilized Liposomal Formulations,” Journal of Pharmaceutical Sciences 103 (2014): 3869–3878. 25308534 10.1002/jps.24171 PMC4441342

[59] M. Friede , M. H. V. Van Regenmortel , and F. Schuber , “Lyophilized Liposomes as Shelf Items for the Preparation of Immunogenic Liposome‐Peptide Conjugates,” Analytical Biochemistry 211 (1993): 117–122. 8323023 10.1006/abio.1993.1241

[60] H. Lee , D. Jiang , and W. M. Pardridge , “Lyoprotectant Optimization for the Freeze‐drying of Receptor‐targeted Trojan Horse Liposomes for Plasmid DNA Delivery,” Molecular Pharmaceutics 17 (2020): 2165–2174. 32315188 10.1021/acs.molpharmaceut.0c00310

[61] M. T. Mabrouk , K. Chiem , E. Rujas , et al., “Lyophilized, thermostable Spike or RBD immunogenic liposomes induce protective immunity against SARS‐CoV‐2 mice,” Science Advances 7 (2021): abj1476. 10.1126/sciadv.abj1476 PMC8635435 34851667

[62] R.‐M. Amarandi , A. Ibanescu , E. Carasevici , L. Marin , and B. Dragoi , “Liposomal‐Based Formulations: A Path From Basic Research to Temozolomide Delivery Inside Glioblastoma Tissue,” Pharmaceutics 14 (2022): 308. 35214041 10.3390/pharmaceutics14020308 PMC8875825

[63] J. J. Sonju , P. Srestha , A. Dahal , et al., “Lyophilized liposomal formulation of a peptidomimetic‐Dox conjugate for HER2 positive breast and lung cancer,” International Journal of Pharmaceutics 639 (2023): 122950. 37059241 10.1016/j.ijpharm.2023.122950

[64] T. Wang , T.‐C. Sung , T. Yu , et al., “Next‐generation materials for RNA–lipid nanoparticles: Lyophilization and targeted transfection,” Journal of Materials Chemistry B 11 (2023): 5083–5093. 37221913 10.1039/d3tb00308f

[65] C. G. Conant , M. C. Schwartz , J. E. Beecher , R. C. Rudoff , C. Iomesco‐Zanetti , and J. T. Nevill , “Well Plate Microfluidic System for Investigation of Dynamic Platelet Behavior Under Variable Shear Loads,” Biotechnology and Bioengineering 108 (2011): 2978–2987. 21702026 10.1002/bit.23243

[66] A. C. Söderström , M. Nybo , C. Nielsen , and P. J. Vinholt , “The Effect of Centrifugation Speed and Time on Preanalytical Platelet Activation,” Clinical Chemistry and Laboratory Medicine 54 (2016): 1913–1920. 27227708 10.1515/cclm-2016-0079

[67] J. Sikora , A. Karczmarska‐Wódzka , J. Bugieda , and P. Sobczak , “The Use of Total Thrombus Formation Analysis System as a Tool to Assess Platelet Function in Bleeding and Thrombosis Risk—A Systematic Review,” International Journal of Molecular Sciences 22 (2021): 8605. 34445311 10.3390/ijms22168605 PMC8395324

[68] D. Whiting and J. A. DiNardo , “TEG and ROTEM: Technology and Clinical Applications,” American Journal of Hematology 89 (2014): 228–232. 24123050 10.1002/ajh.23599

[69] V. Aragon‐Sanabria , S. E. Pohler , V. J. Eswar , M. Bierowski , E. W. Gomez , and C. Dong , “VE‐Cadherin Disassembly and Cell Contractility in the Endothelium Are Necessary for Barrier Disruption Induced by Tumor Cells,” Scientific Reports 7 (2017): 45835. 28393886 10.1038/srep45835 PMC5385522

[70] T. Nightingale and D. Cutler , “The secretion of von Willebrand factor From endothelial cells; an increasingly complicated story,” Journal of Thrombosis and Haemostasis 11 (2014): 192–201. 10.1111/jth.12225 PMC4255685 23809123

[71] T. Hoppenbrouwers , A. S. A. Autar , A. R. Sultan , et al., “In Vitro induction of NETosis: Comprehensive live imaging comparison and systematic review,” PLoS One 12 (2017): 0176472. 10.1371/journal.pone.0176472 PMC5423591 28486563

[72] N. de Buhr and M. von Köckritz‐Blickwede , “How Neutrophil Extracellular Traps Become Visible,” Journal of Immunology Research 2016 (2016): 1–13. 10.1155/2016/4604713 PMC4884809 27294157

[73] N. M. La‐Beck , M. R. Islam , and M. M. Markiewski , “Nanoparticle‐induced Complement Activation: Implications for Cancer Nanomedicine,” Frontiers in Immunology 11 (2021): 603039. 33488603 10.3389/fimmu.2020.603039 PMC7819852

[74] Y. Morodomi , S. Kanaji , E. Won , Z. M. Ruggeri , and T. Kanaji , “Mechanisms of Anti‐GPIbα Antibody–induced Thrombocytopenia in Mice,” Blood 135 (2020): 2292–2301. 32157300 10.1182/blood.2019003770 PMC7316218

[75] T. K. Greene , A. Schiviz , W. Hoellriegl , M. Poncz , and E. M. Muchitsch , “Towards a standardization of the murine tail bleeding model,” Journal of Thrombosis and Haemostasis 8 (2010): 2820–2822. 21138523 10.1111/j.1538-7836.2010.04084.x

[76] A. Sen Gupta , “Bio‐inspired Nanomedicine Strategis for Artificial Blood Components,” Wiley Interdisciplinary Reviews: Nanomedicine and Nanobiotechnology 9, no. 10 (2017): 1002. 10.1002/wnan.1464 PMC5599317 28296287

[77] L. Shaffer , “Making and storing blood to save lives,” Proceedings of the National Academy of Sciences 117 (2020): 7542–7545. 10.1073/pnas.2001649117 PMC7148556 32238555

[78] T. J. Pichon , N. J. White , and S. H. Pun , “Engineered Intravenous Therapies for Trauma,” Current Opinion in Biomedical Engineering 27 (2023): 100456. 37456984 10.1016/j.cobme.2023.100456 PMC10343715

[79] L. J. Tucker , K. Hilmas , and A. C. Brown , “Structure‐based Design of Therapeutics to Control Hemostasis,” Blood 146, no. 12 (2025): 1431–1439. 40132157 10.1182/blood.2024025323 PMC12883855

[80] M. S. Gatto and W. Najahi‐Missaoui , “Lyophilization of Nanoparticles, Does It Really Work? Overview of the Current Status and Challenges,” International Journal of Molecular Sciences 24 (2023): 14041. 37762348 10.3390/ijms241814041 PMC10530935

[81] S. Gould and R. C. Scott , “2‐Hydroxypropyl‐β‐cyclodextrin (HP‐β‐CD): A toxicology review,” Food and Chemical Toxicology 43 (2005): 1451–1459. 16018907 10.1016/j.fct.2005.03.007

[82] S. S. C. Braga , “Cyclodextrins: Emerging Medicines of the New Millennium,” Biomolecules 9 (2019): 801. 31795222 10.3390/biom9120801 PMC6995511

[83] R. H. Lee , R. Piatt , A. Dhenge , et al., “Impaired Hemostatic Activity of Healthy Transfused Platelets in Inherited and Acquired Platelet Disorders: Mechanisms and Implications,” Science Translational Medicine 11 (2019): aay0203. 10.1126/scitranslmed.aay0203 PMC10824274 31826978

[84] C. Karlström , G. Gryfelt , L. Schmied , S. Meinke , and P. Höglund , “Platelet Transfusion Improves Clot Formation and Platelet Function in Severely Thrombocytopenic Haematology Patients,” British Journal of Haematology 196 (2022): 224–233. 34528253 10.1111/bjh.17820

[85] Z. Liu , A. Abdullah , Z. A. Secunda , et al., “Intraosseous Administration of Lyophilized Syntheticplatelets Renders Hemostatic Efficacy in Rat Model of Traumatic Hemorrhage,” Journal of Thrombosis and Haemostasis 24 (2026): 877–889. 41224176 10.1016/j.jtha.2025.10.030

[86] M. Barry and S. Pati , “Targeting Repair of the Vascular Endothelium and Glycocalyx After Traumatic Injury With Plasma and Platelet Resuscitation,” Matrix Biology Plus 14 (2022): 100107. 35392184 10.1016/j.mbplus.2022.100107 PMC8981767

[87] S. J. Feuerstein , K. Skovmand , A. M. Møller , and K. Wildgaard , “Freeze‐dried plasma in major haemorrhage: A systematic review,” Vox Sang 115 (2020): 263–274. 32090336 10.1111/vox.12898

[88] A. Zaleski , “There Will be Blood,” Science 385 (2024): 16–20. 38963853 10.1126/science.adr4067

[89] H. Zhang , “Thin‐film Hydration Followed by Extrusion Method for Liposome Preparation,” Methods in Molecular Biology 1522 (2017): 17–22. 27837527 10.1007/978-1-4939-6591-5_2

[90] C. G. Conant , J. T. Nevill , Z. Zhou , J.‐F. Dong , M. A. Schwartz , and C. Ionescu‐Zanetti , “Using Well‐plate Microfluidic Devices to Conduct Shear‐based Thrombosis Assays,” JALA: Journal of the Association for Laboratory Automation 16 (2011): 148–152. 10.1016/j.jala.2010.10.005 21609696

[91] I. Ashworth , L. Thielemans , and T. Chevassut , “Thrombocytopenia: The Good, the Bad and the Ugly,” Clinical Medicine 22 (2022): 214–217. 35584828 10.7861/clinmed.2022-0146 PMC9135082

[92] A. M. Lima , D. S. Saint Auguste , F. Cuenot , et al., “Standardization and Validation of Fluorescence‐based Quantitative Assay to Study human Platelet Adhesion to Extracellular‐matrix in a 384‐well Plate,” International Journal of Molecular Sciences 21 (2020): 6539. 32906775 10.3390/ijms21186539 PMC7554887

[93] Y. Cao , Y. Gong , L. Liu , et al., “The Use of human Umbilical Vein Endothelial Cells (HUVECs) as an in Vitro Model to Assess the Toxicity of Nanoparticles to Endothelium: A Review,” Journal of Applied Toxicology 37 (2017): 1359–1369. 28383141 10.1002/jat.3470

[94] B. Marcos‐Ramiro , D. García‐Weber , and J. Millián , “TNF‐induced Endothelial Barrier Disruption: Beyond Actin and Rho,” Thromb Haemost 112 (2014): 1088–1102. 25078148 10.1160/TH14-04-0299

[95] L. A. Madge and J. S. Pober , “TNF Signaling in Vascular Endothelial Cells,” Experimental and Molecular Pathology 70 (2001): 317–325. 11418010 10.1006/exmp.2001.2368

[96] V. Papayannopoulos , “Neutrophil Extracellular Traps in Immunity and Disease,” Nature Reviews Immunology 18 (2018): 134–147. 10.1038/nri.2017.105 28990587

