# Supplementary material for: Lyophilized Synthetic Platelets: In Vitro Characterization and In Vivo Evaluation in Mouse Thrombocytopenia Model
Source: Adv Sci (Weinh). 2026 Apr 20;13(38):e00002. doi: 10.1002/advs.202600002 (PMC13335430; doi:10.1002/advs.202600002)
Supplement: Supplementary file 1 — Supporting File 1: advs75336‐sup‐0001‐SuppMat.docx. [file ADVS-13-e00002-s007.docx]

**Lyophilized Synthetic Platelets: In Vitro Characterization**

**and In Vivo Evaluation in Mouse Thrombocytopenia Model**

*Ujjal Didar Singh Sekhon^1^, Dante Disharoon^2^, Shrijal S. Desai^1^, Baylee Traylor^1^,*

*Emily Gahagan^1^, Emma Quill^1^, Kristin Aldridge^1^, Norman Luc^2^, Sonali Rohiwal^2^,*

*Shruti Raghunathan^2^, Rebecca Ahn^1^, Sana Syed^1^, Alexander Dornback^1^,*

*Bipin Chakravarthy Paruchuri^2^, Andrew Ditto^1^, Susan M. Shea^3,4^, Philip C. Spinella^3^,*

*Matthew D. Neal^3^, Michael A. Bruckman^1^, Christa L. Pawlowski^1,*^, Anirban Sen Gupta^1,2,*^*

**Affiliations**

^1^Haima Therapeutics LLC, Cleveland, OH 44106, USA

^2^ Case Western Reserve University, Department of Biomedical Engineering, Cleveland, OH

44106, USA

^3^University of Pittsburgh, Trauma and Transfusion Medicine Research Center, Department of

Surgery, Pittsburgh, PA 15123, USA

^4^University of Pittsburgh, Department of Bioengineering, Pittsburgh, PA 15213, USA

* Co-Corresponding Authors:

Anirban Sen Gupta, PhD

Case Western Reserve University

10900 Euclid Avenue, Wickenden Building Rm 202

Cleveland Ohio 44106, USA

Phone: (01) 216-368-4564

E-Mail: [axs262@case.edu](mailto:axs262@case.edu)

Christa Pawlowski, PhD

Haima Therapeutics

11000 Cedar Avenue, Suite 280

Cleveland Ohio 44106, USA

Phone: (01) 803-727-9487

E-mail: [cpawlowski@haimatherapeutics.com](mailto:cpawlowski@haimatherapeutics.com)

**Funding:** United States Department of Defense grant W81XWH-20-1-0628 to A.S.G. and

W81XWH-17-2-0064 to Haima Therapeutics.

Ujjal Didar Singh Sekhon and Dante Disharoon are co-first authors as they contributed equally.


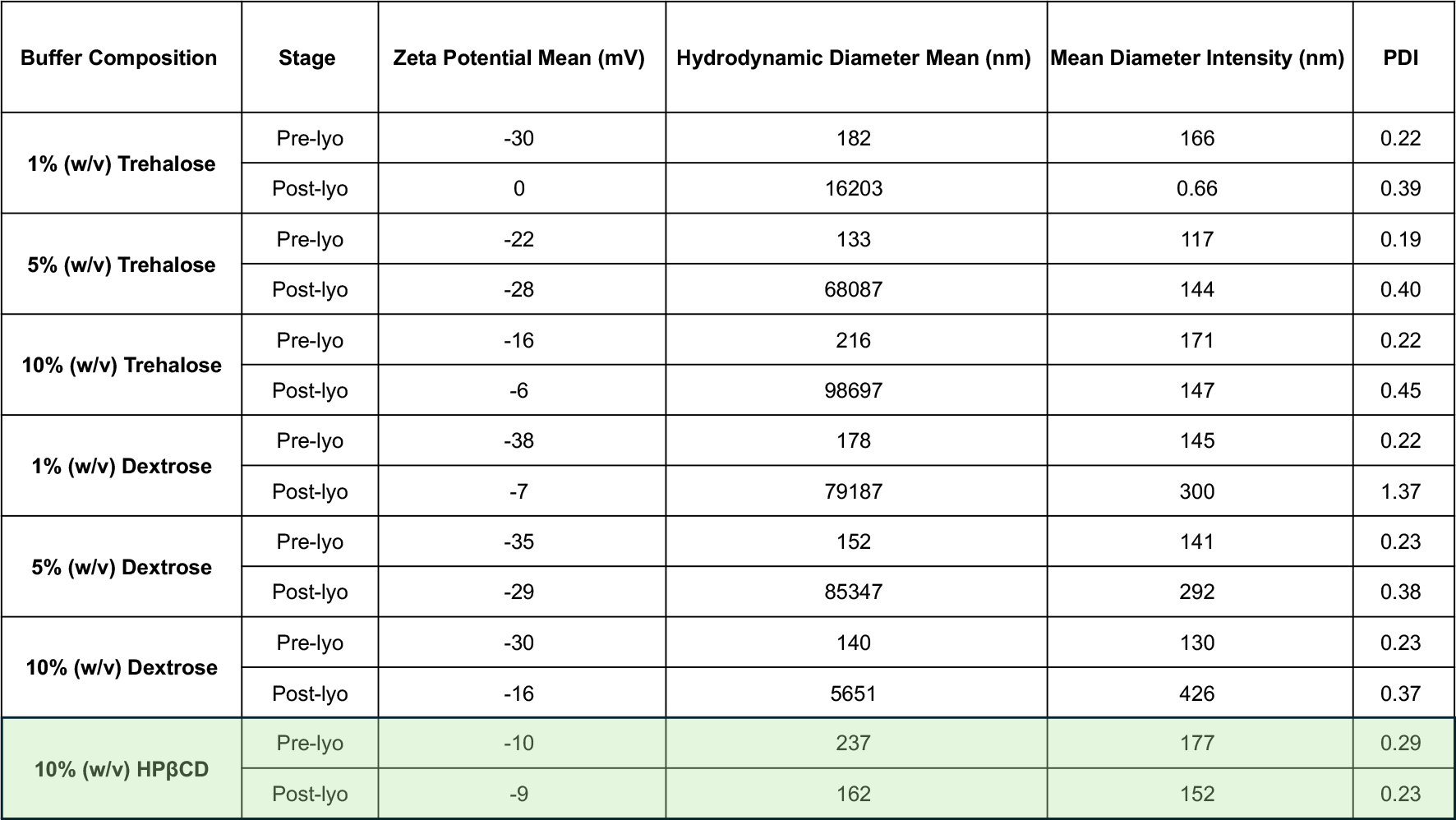


**Figure S1.** Representative dataset comparing size distribution, polydispersity and zeta potential of SP (pre-lyo) vs. Lyo-SP (post-lyo) utilizing Trehalose vs. Dextrose vs. HPβCD as lyoprotectants at various concentrations; Additional example comparative datasets are available in publicly released technical reports for contract W81XWH-20-1-0628 from the US Department of Defense that supported the process development and optimization of the SP-to-LyoSP advancement (e.g. <https://apps.dtic.mil/sti/trecms/pdf/AD1169478.pdf>); As evident from the data, Trehalose and Dextrose failed to conserve optimal size and morphological parameters of reconstituted Lyo-SP, but HPβCD was able to successfully achieve this, leading to its selection as the lyoprotectant of choice for all Lyo-SP manufacture and evaluation.


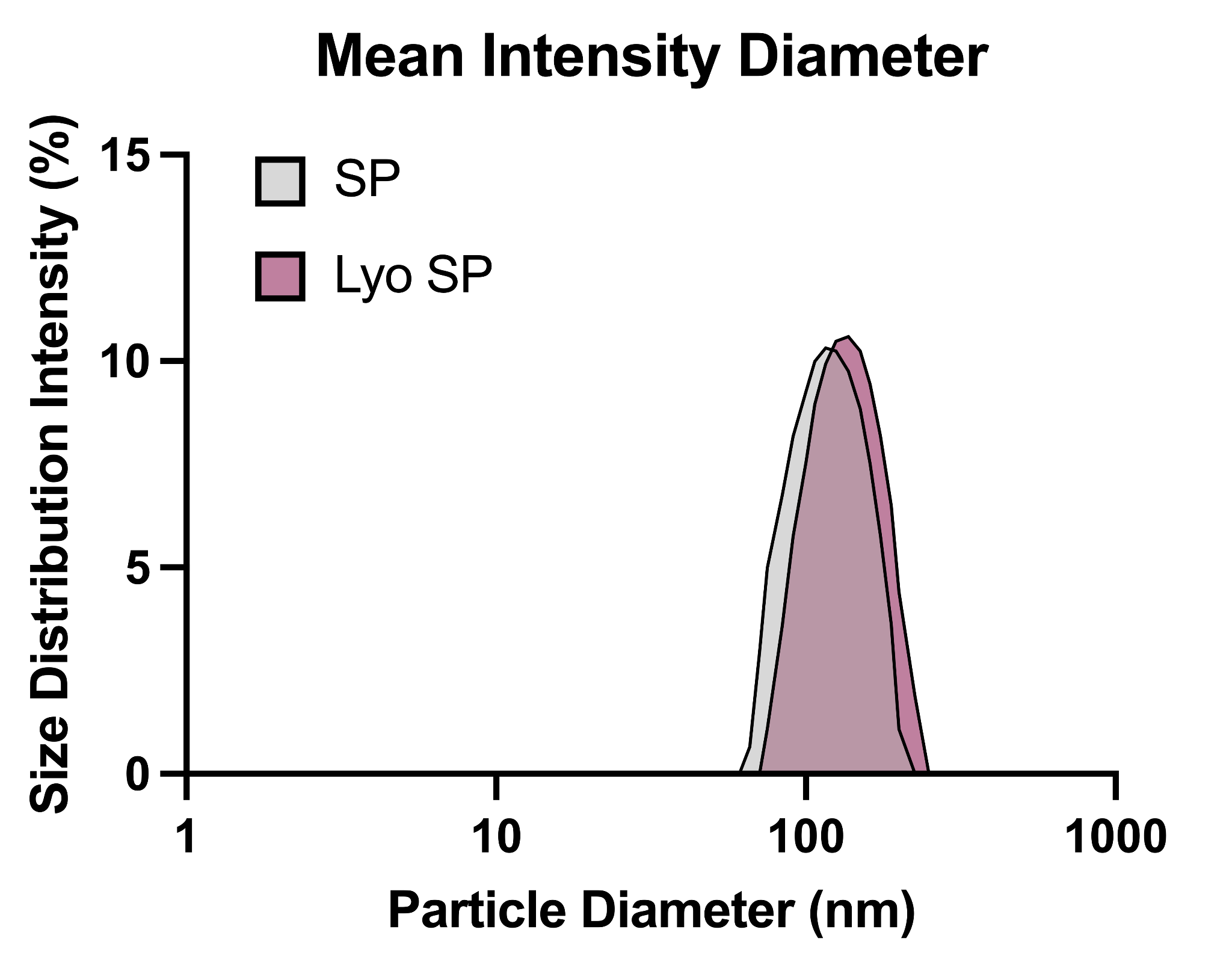


**Figure S2.** Representative data for Dynamic Light Scattering (DLS) based characterization of fresh-made SP and aqueous-reconstituted Lyo-SP, demonstrating that the size distribution of SP is conserved in reconstituted Lyo-SP, indicating particle stability post-lyophilization.


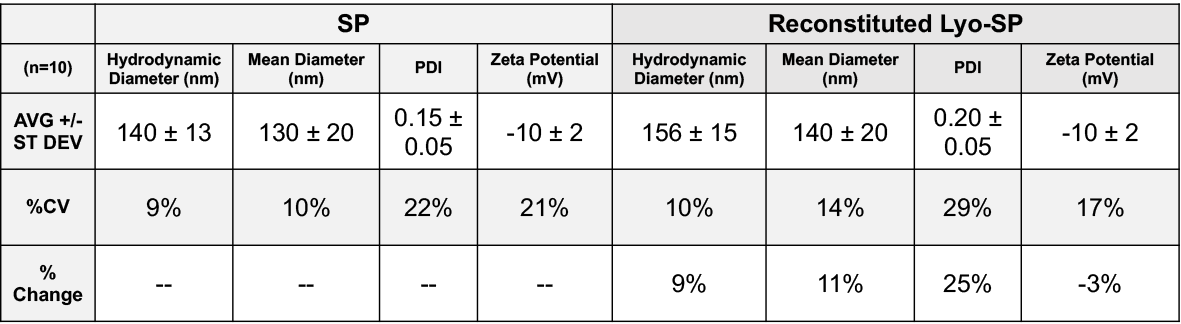


**Figure S3.** Representative quantitative data table for Dynamic Light Scattering (DLS) based characterization of fresh-made SP and aqueous-reconstituted Lyo-SP, demonstrating that the particle diameter of SP is conserved in reconstituted Lyo-SP, indicating morphological stability.


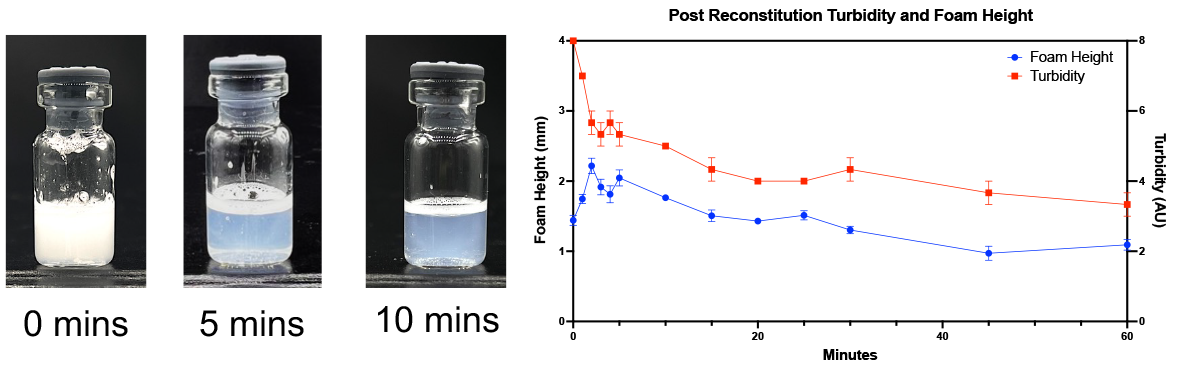


**Figure S4.** Representative photographs and data for turbidity and foam height of reconstituted Lyo-SP (reconstitution process shown in **Movie M1**), indicating rapid and stable reconstitution.


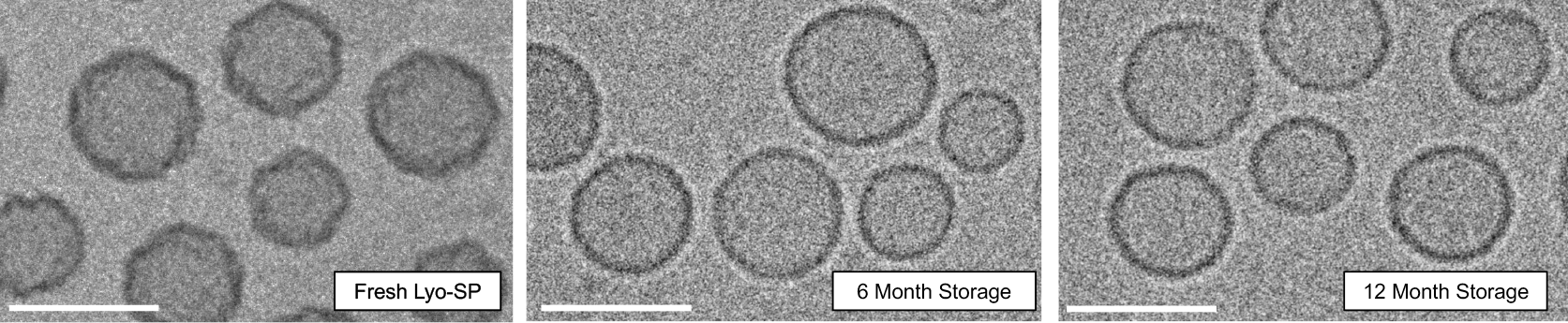


**Figure S5.** Representative cryo-TEM images of aqueous-reconstituted Lyo-SP immediately after manufacture (fresh Lyo-SP), after 6-month storage and after 12-month storage at room temperature indicating storage-stability of morphology and size..


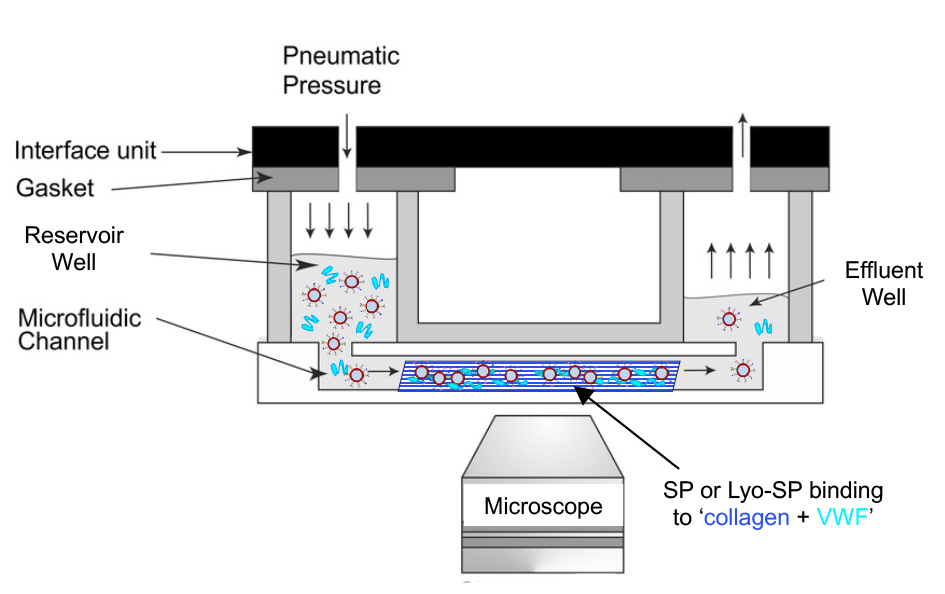


**Figure S6. BioFlux microfluidic set-up to study binding of SP vs. Lyo-SP on ‘collagen + VWF’ surface:** Experimental set-up of BioFlux microfluidic system where microfluidic channels were coated with collagen (indigo blue), and soluble VWF (cyan) was flowed over the collagen-coated channel at high shear (60 dyn/cm^2^) to image and confirm the assembly of VWF on collagen; The flow velocity was pneumatically controlled so as to allow sufficiently high shear (60 dyn/cm^2^) for VWF unfolding and assembly on collagen; Using the same set-up, red fluorescent SP or Lyo-SP nanoparticles were flowed over the collagen-coated channel in presence of soluble VWF and the nanoparticle binding to the surface was imaged to quantify the fluorescence intensity of the surface-bound nanoparticles; Flow of SP or Lyo-SP in the same set-up but over BSA-coated channel was used as the negative control.


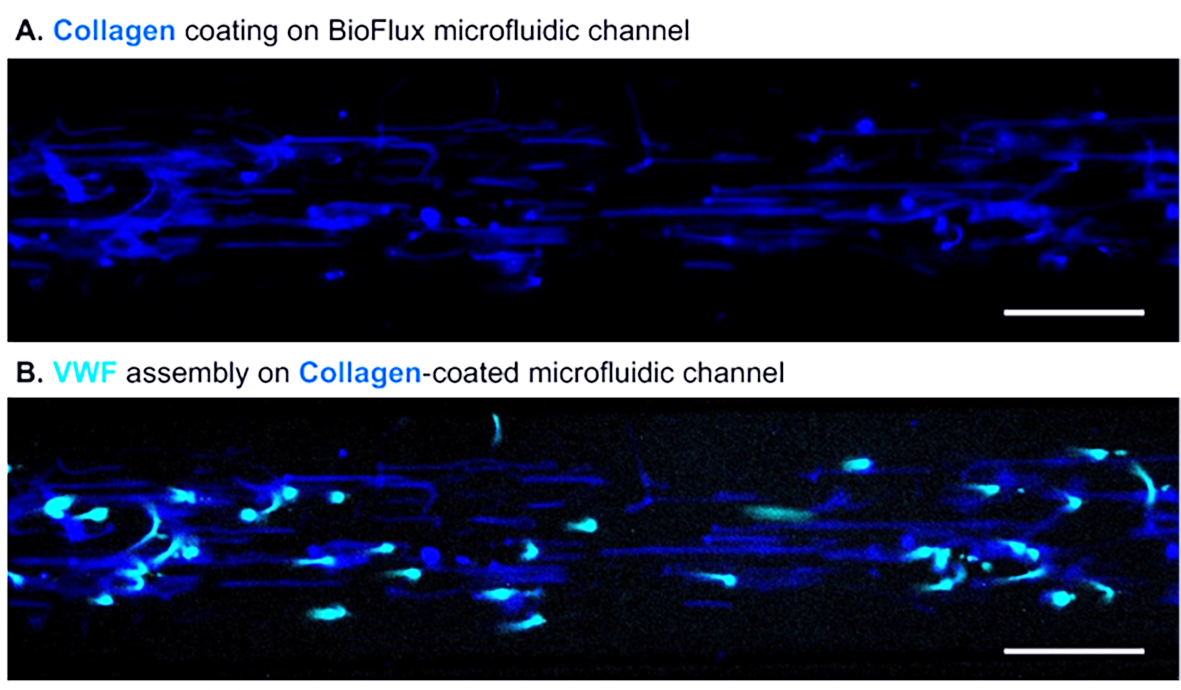


**Figure S7.** **A**: Representative image of collagen (indigo blue) coated on BioFlux microfluidic channel surface; **B**: Representative image of VWF (cyan) assembled on the collagen-coated microfluidic channel surface; The results confirm that ‘collagen + VWF’ are present on the channel surface to allow adherence of SP or Lyo-SP.

**
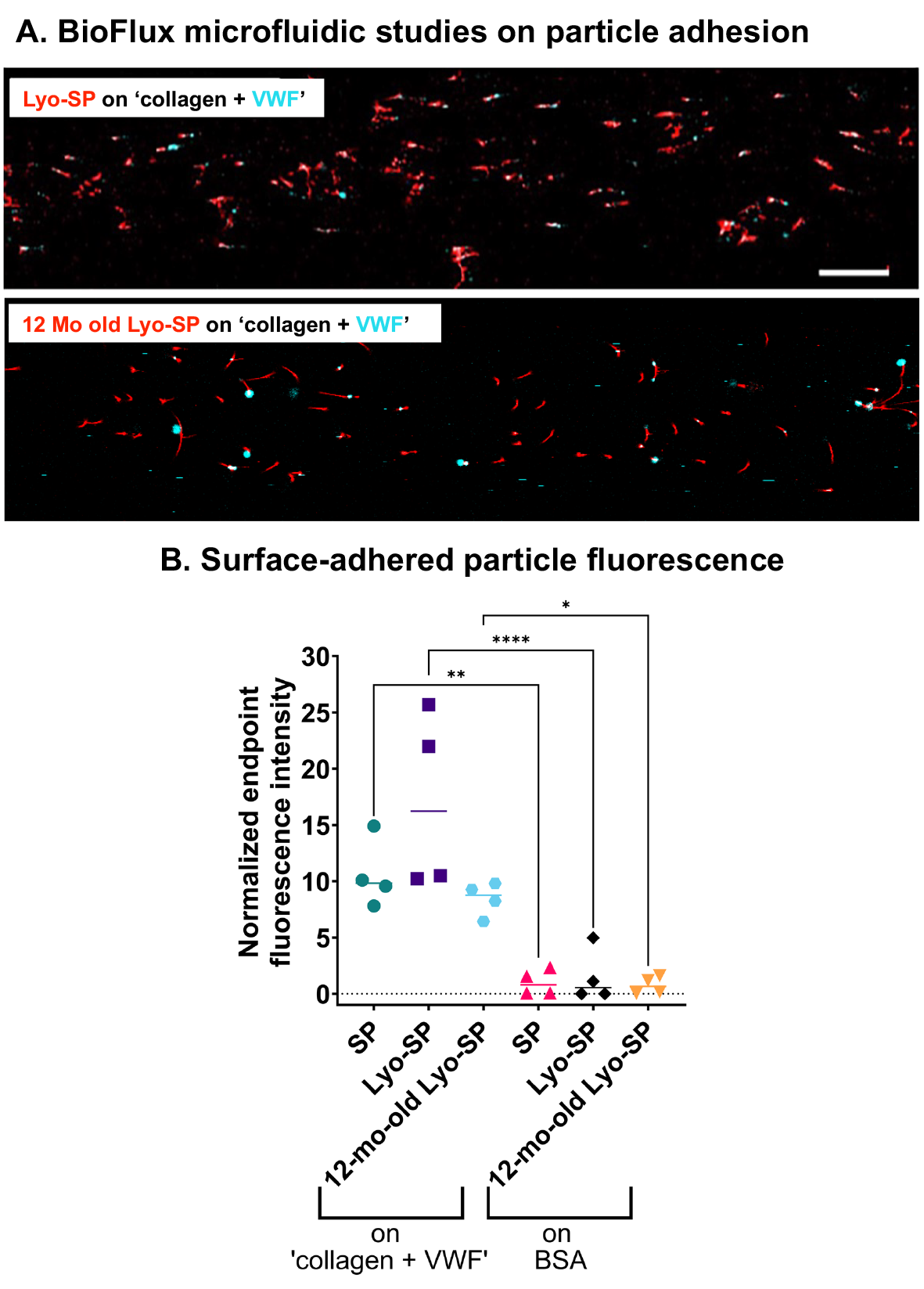
**

**Figure S8.** BioFlux microfluidics-based analysis comparing the adhesion of 12-month stored Lyo-SP vs. fresh Lyo-SP binding to collagen-coated microfluidic channel in presence of soluble VWF under high shear flow, confirms that 12-month stored Lyo-SP retains the ability to significantly adhere to the ‘collagen + VWF’ surface at levels similar to fresh Lyo-SP, which is also equivalent to pre-lyophilized SP (see data shown in **Main Figure 3A** and **3B**); SP, fresh Lyo-SP and 12-month stored Lyo-SP all undergo minimal adhesion on albumin (BSA)-coated ‘negative control’; surface; These results confirm that the activity of VBP and CBP peptides to render platelet-mimetic adhesion to VWF and collagen respectively are conserved in 12-month stored Lyo-SP.


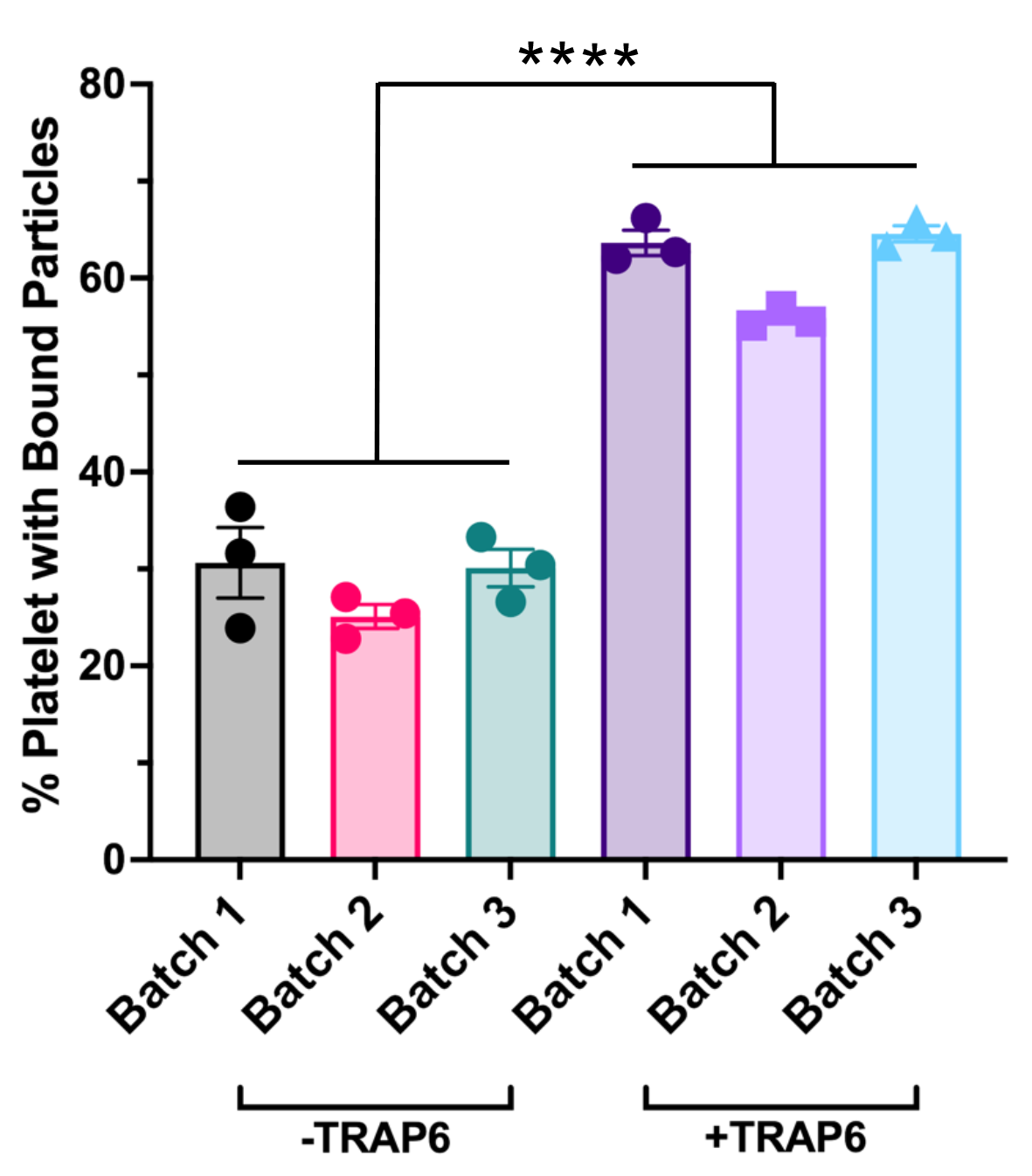


**Figure S9.** Flow cytometry analysis of three different batches of Lyo-SP binding to platelets isolated from three different blood donors on different days for each batch shows that without TRAP-6 induced activation all three batches of Lyo-SP have low binding to platelets while with TRAP-6 induced platelet activation all three batches have enhanced binding to platekets; The results confirm that Lyo-SP can reproducibly undergo enhanced binding to activated platelets even though there may be slight variabilities between technical replicates (different batches of Lyo-SP) and biological replicates (different blood donors).


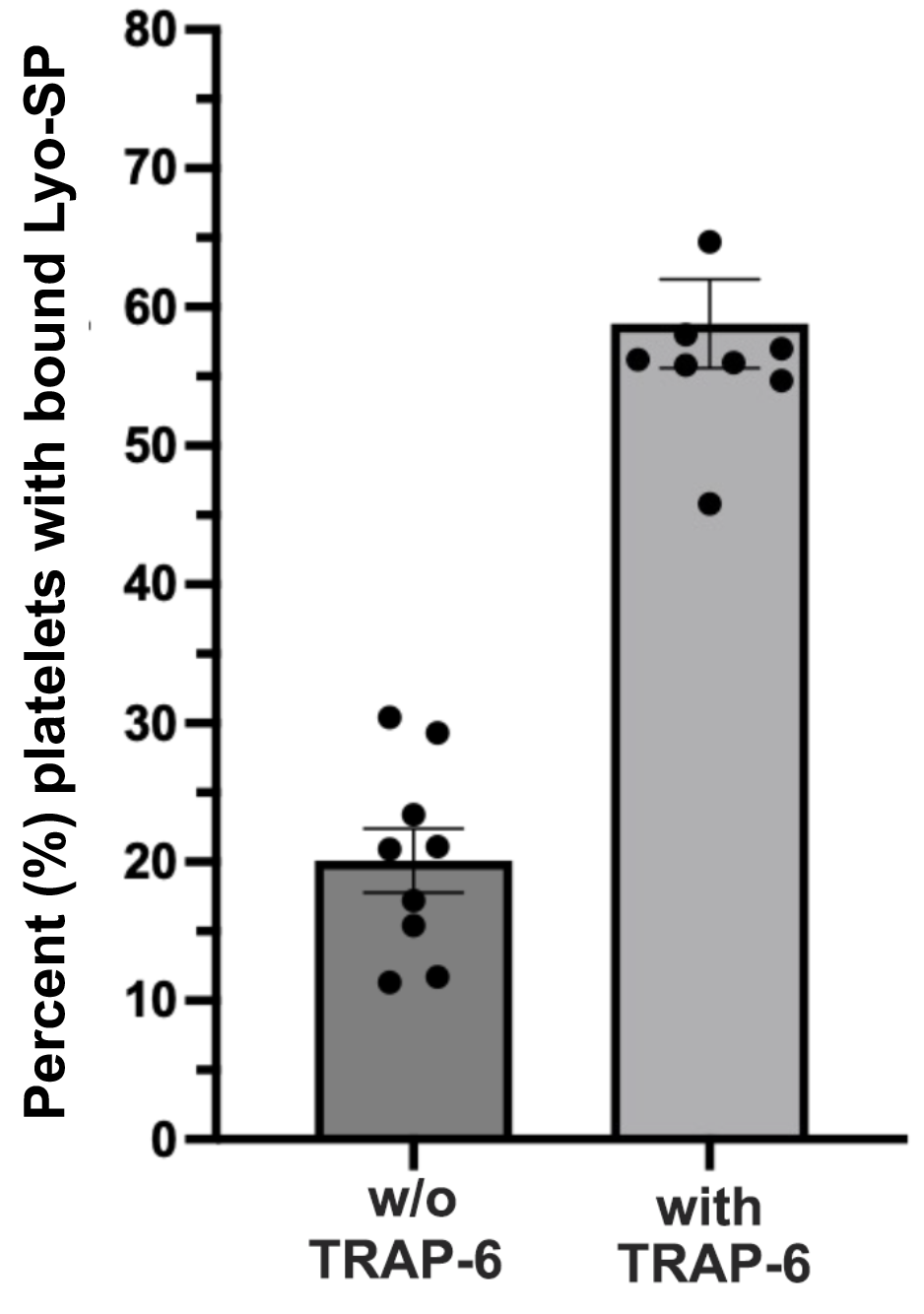


**Figure S10.** Flow cytometry analysis of 12-month stored Lyo-SP binding to predominantly inactive platelets (w/o TRAP-6 activation) vs. agonist-activated platelets (with TRAP-6 activation) shows that Lyo-SP binding was markedly higher in the activated (with TRAP-6) platelet population (58.79 +/- 3.19 %) compared to that w/o TRAP-6 activation (20.08 +/- 2.30%); These results confirm that the FMP activity for platelet GPIIb/IIIa binding is conserved on 12-month stored Lyo-SP at levels comparable to fresh Lyo-SP (see **Main Figure 3D** for reference).


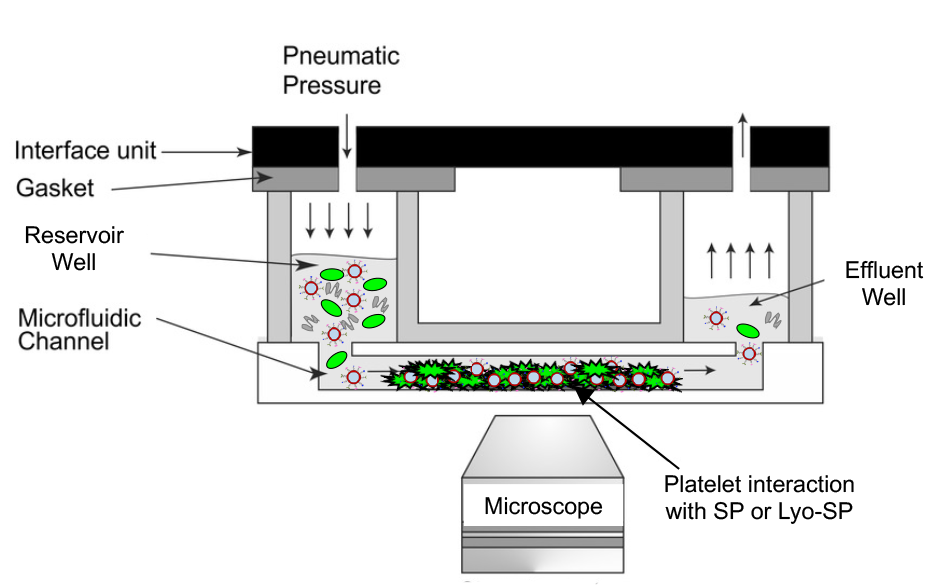


**Figure S11. BioFlux microfluidic set-up to study effect of SP vs. Lyo-SP in thrombocytopenic human plasma:** Calcein-stained (green fluorescent) platelets were flowed over the channel surface along with Cy5-labeled (red fluorescent) SP or Lyo-SP nanoparticles, in presence of soluble VWF (not fluorescent) in plasma; The flow velocity (hence shear) was pneumatically controlled so as to render high shear (60 dyn/cm^2^) for VWF unfolding; Fluorescence microscopy based real time imaging was carried out to analyze platelet accumulation on the ‘collagen + VWF’ surface in PRP vs. TCP condition, as well as to analyze SP or Lyo-SP interaction with platelets in TCP condition for hemostatic effect; Platelet accumulation kinetics and endpoint image analyses were used to quantify channel surface coverage by platelets and platelet-particle colocalization.


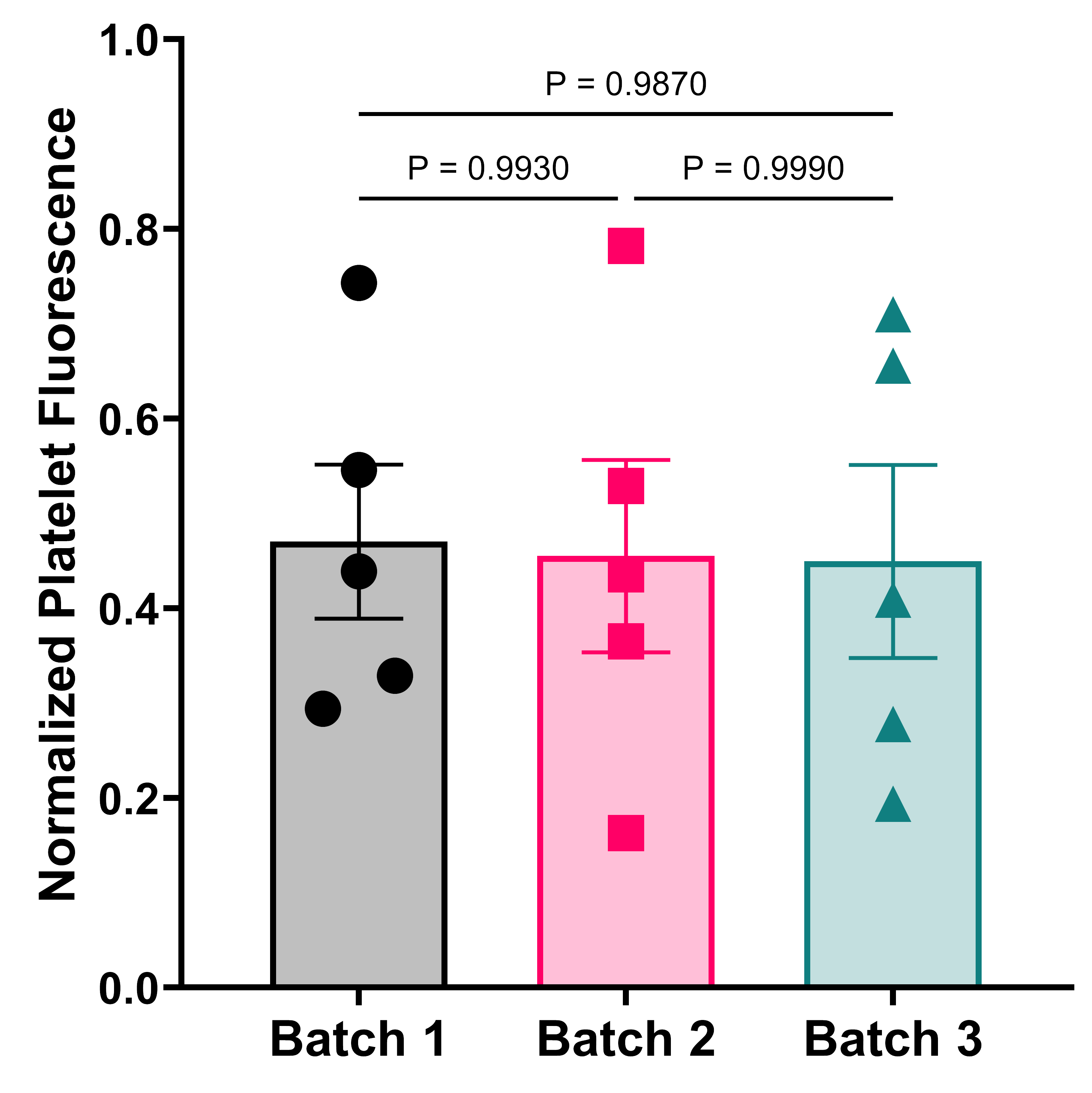


**Figure S12. Reproducibility of Lyo-SP effect regarding rescue of platelet accumulation in TCP condition in BioFlux:** Effect of three batches of Lyo-SP on platelet accumulation (reflected by normalized platelet fluorescence) in thrombocytopenic plasma (TCP) setting tested for five different donors per batch shows batch-to-batch reproducibility of Lyo-SP hemostatic effect.


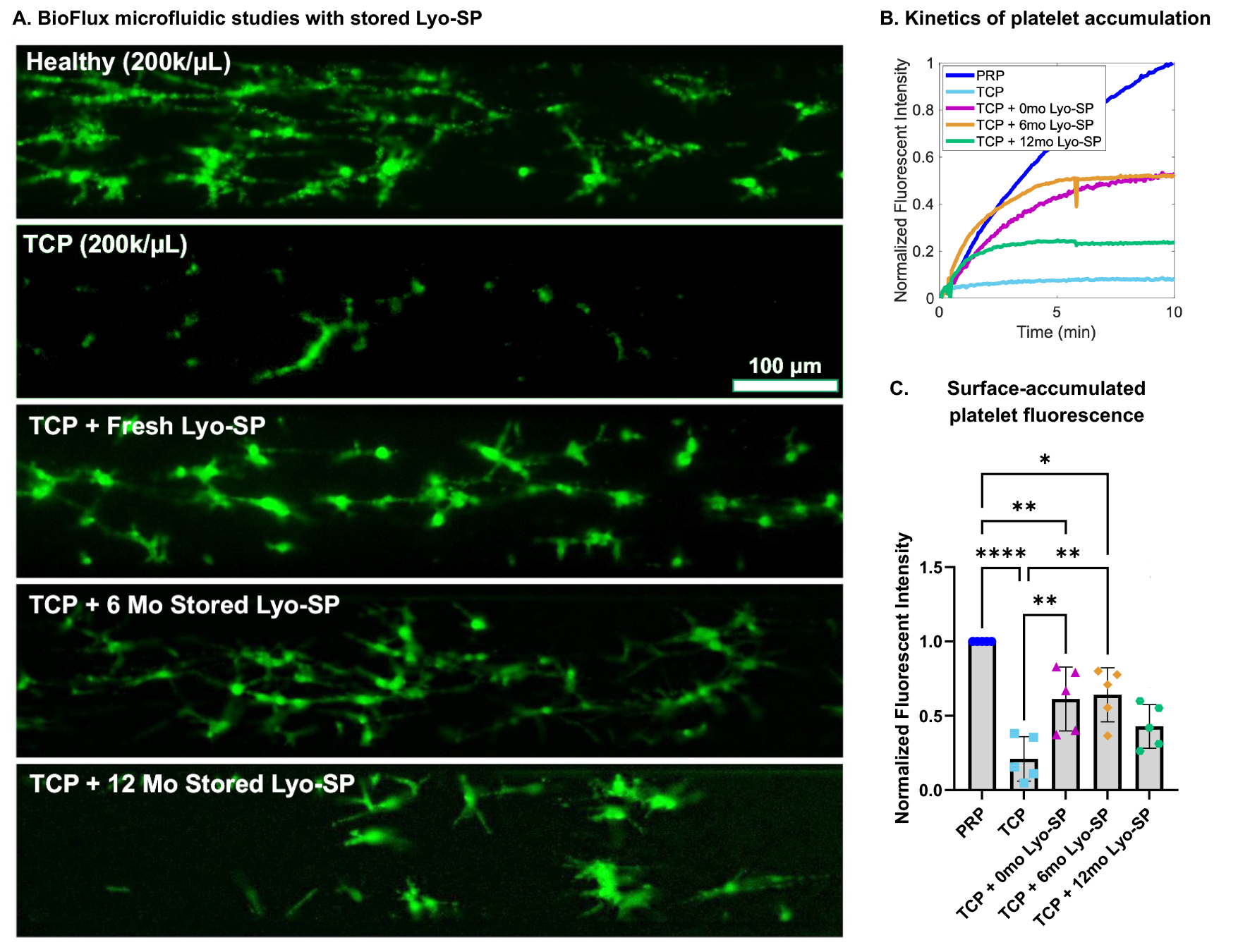


**Figure S13. BioFlux microfluidic studies to analyze effect of fresh Lyo-SP (0 months) vs. 6-month stored and 12-month stored Lyo-SP in thrombocytopenic human plasma:** Calcein-stained (green fluorescent) platelets in plasma were flowed at 60 dyn/cm^2^ over collagen-coated BioFlux microfluidic channel surface along with fresh (0 months) or 6-month stored or 12-month stored Lyo-SP nanoparticles, in the presence of soluble VWF (note: collagen, vWF and Lyo-SP were not fluorescently labeled in these studies); **A:** Representative endpoint images of platelet accumulation on the channel surface in healthy platelet-rich-plasma (PRP) vs. thrombocytopenic plasma (TCP) vs. TCP treated with fresh (0 months) or 6-month stored or 12-month stored Lyo-SP; **B:** Representative kinetic curves of platelet accumulation from the above studies over 10-minute flow period on channel surface; **C:** Statistical analysis of channel surface-accumulated platelet fluorescence at the endpoint of experiment.


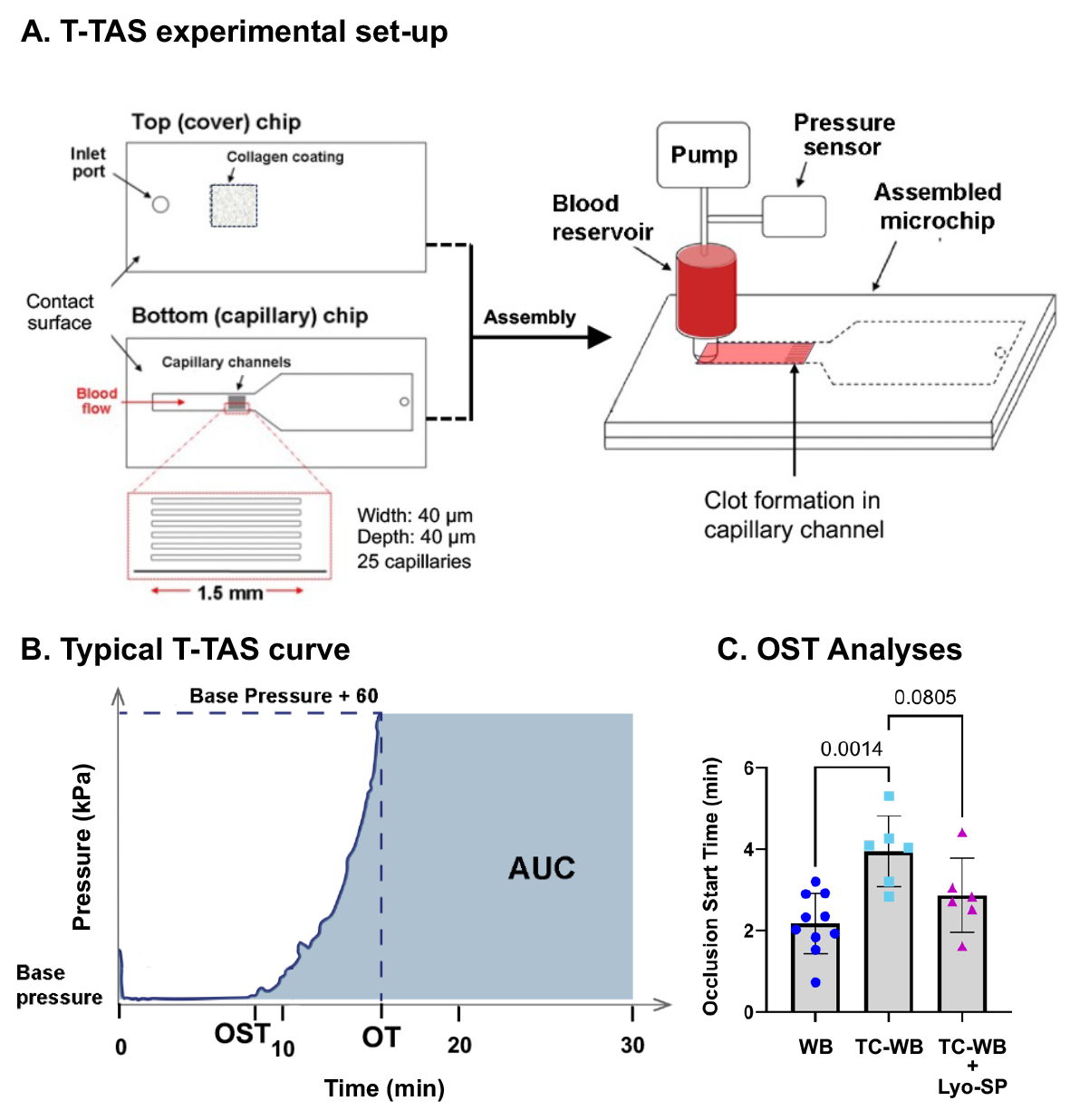
**Figure S14. A:** T-TAS experimental set-up to study effect of Lyo-SP in thrombocytopenic human whole blood; **B:** Typical T-TAS PL-chip Pressure vs. Time curve showing analyzed parameters of Occlusion Start Time (OST), Occlusion Time (OT) and Area under Curve (AUC); **C:** OST analyses results for WB vs TC-WB vs ‘TC-WB + Lyo-SP’ shows that platelet-depleted WB (i.e. TC-WB) had a significant delay in the start of channel occlusion compared to healthy WB, and treatment of TC-WB with Lyo-SP showed a substantial trend toward rescuing the clotting kinetics from this initial delay.


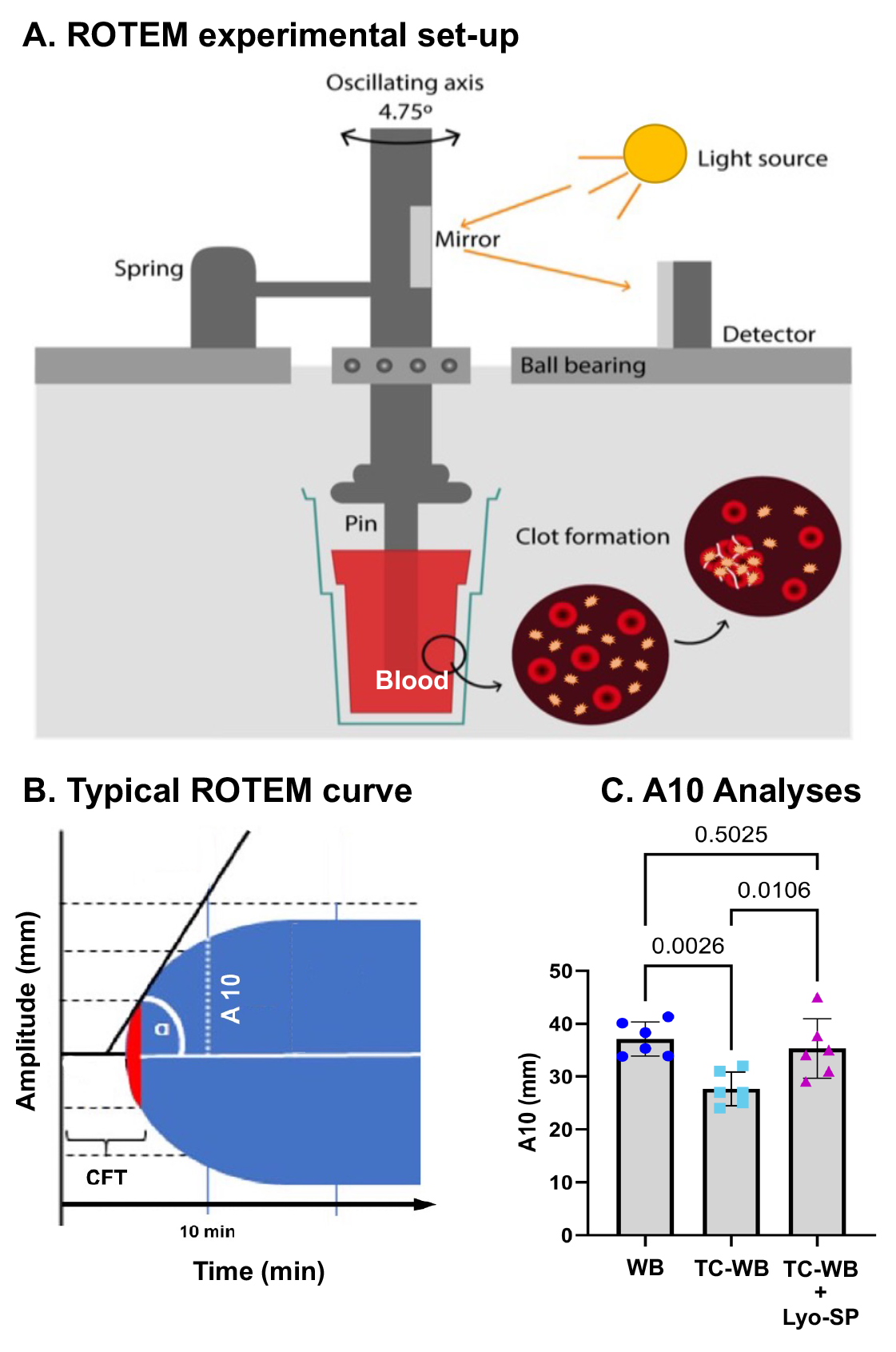


**Figure S15. A:** ROTEM experimental set-up to study effect of Lyo-SP in human whole blood; **B:** Typical ROTEM curve (TEM-ogram) showing analyzed parameters of Clot Formation Time (CFT), alpha angle (α) and amplitude 10 min after clotting time (A10); **C:** A10 analyses results for WB vs TC-WB vs ‘TC-WB + Lyo-SP’ shows that platelet-depleted WB (i.e. TC-WB) has reduced A10 (reflective of compromised clot growth), and treatment of TC-WB with Lyo-SP showed a significant rescue of this parameter, indicating hemostatic rescue.


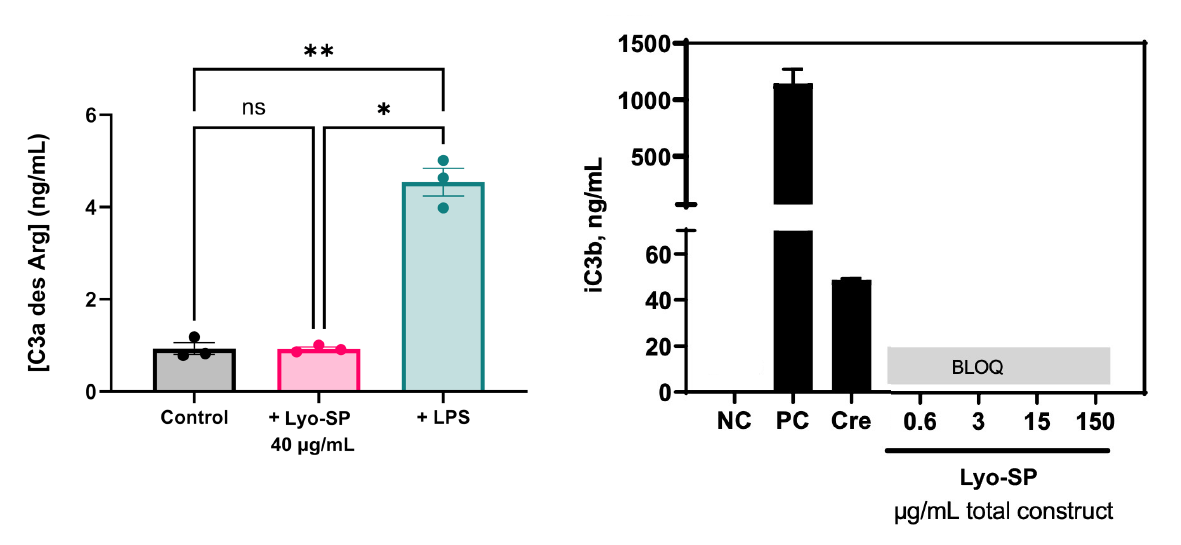


**Figure S16.** In vitro analysis of Lyo-SP on Complement C3 activation in human plasma studied by C3a ELISA (conducted at CWRU by co-author Norman Luc) and C3b ELISA (conducted via Haima contract at Nanotechnology Characterization Laboratory, NIH); Lipopolysaccharide (LPS) was used as positive control (PC) for C3🡪C3a activation, and Cobra Venom Factor (Quidel Corp., A600) was used as a positive control (PC) for C3 🡪 C3b activation; Cremophor EL (Cre) was used as a second comparison control for C3b analysis; Negetive control (NC) in both tests used phosphate-buffered saline; Compared to negative control baseline, positive control treated plasma showed significantly higher C3 activation (both C3a and C3b), while Lyo-SP-incubatd plasma showed signal statistically similar C3a level to negative control and C3b signal below level of quantification (BLOQ), indicating minimal risk of C3 activation to both C3a and C3b.


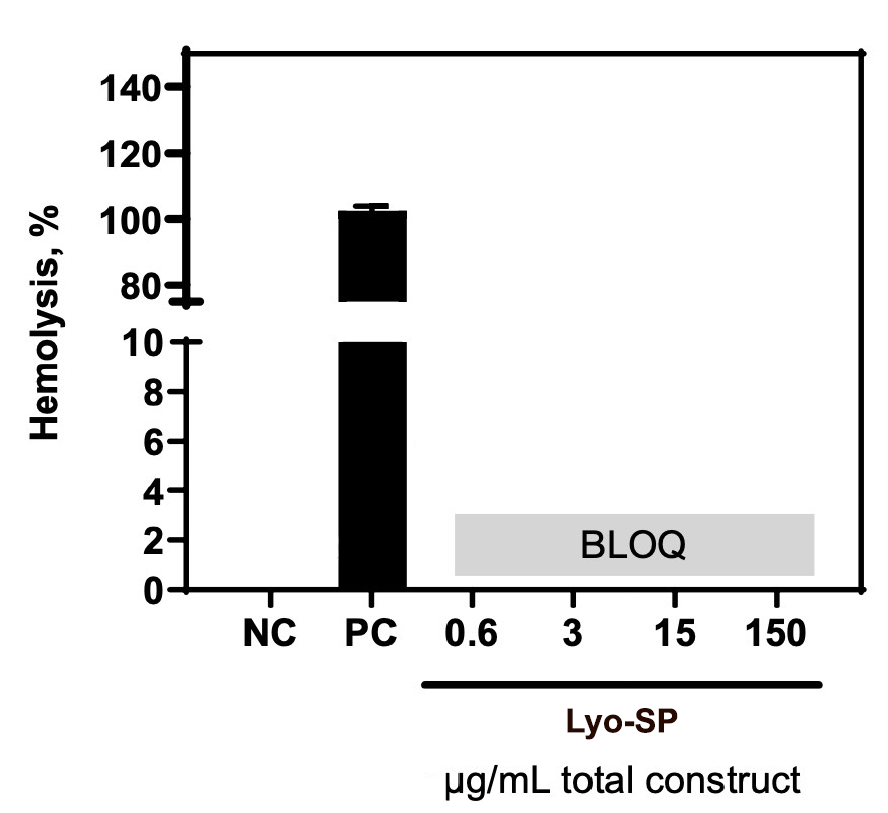


**Figure S17.** Hemolysis assessment with human blood (conducted via Haima contract at Nanotechnology Characterization Laboratory, NIH) of Lyo-SP at increasing concentrations show minimal hemolytic effect (BLOQ: Below Level of Quantification) compared to positive control (PC); Considering average human blood volume to be 70 ml/kg, a Lyo-SP concentration of 150 μg/ml corresponds to approximately 10.5 mg/kg dose which is on the higher side of therapeutic dose and this still shows minimal hemolysis, indicating safety signature of Lyo-SP in blood.


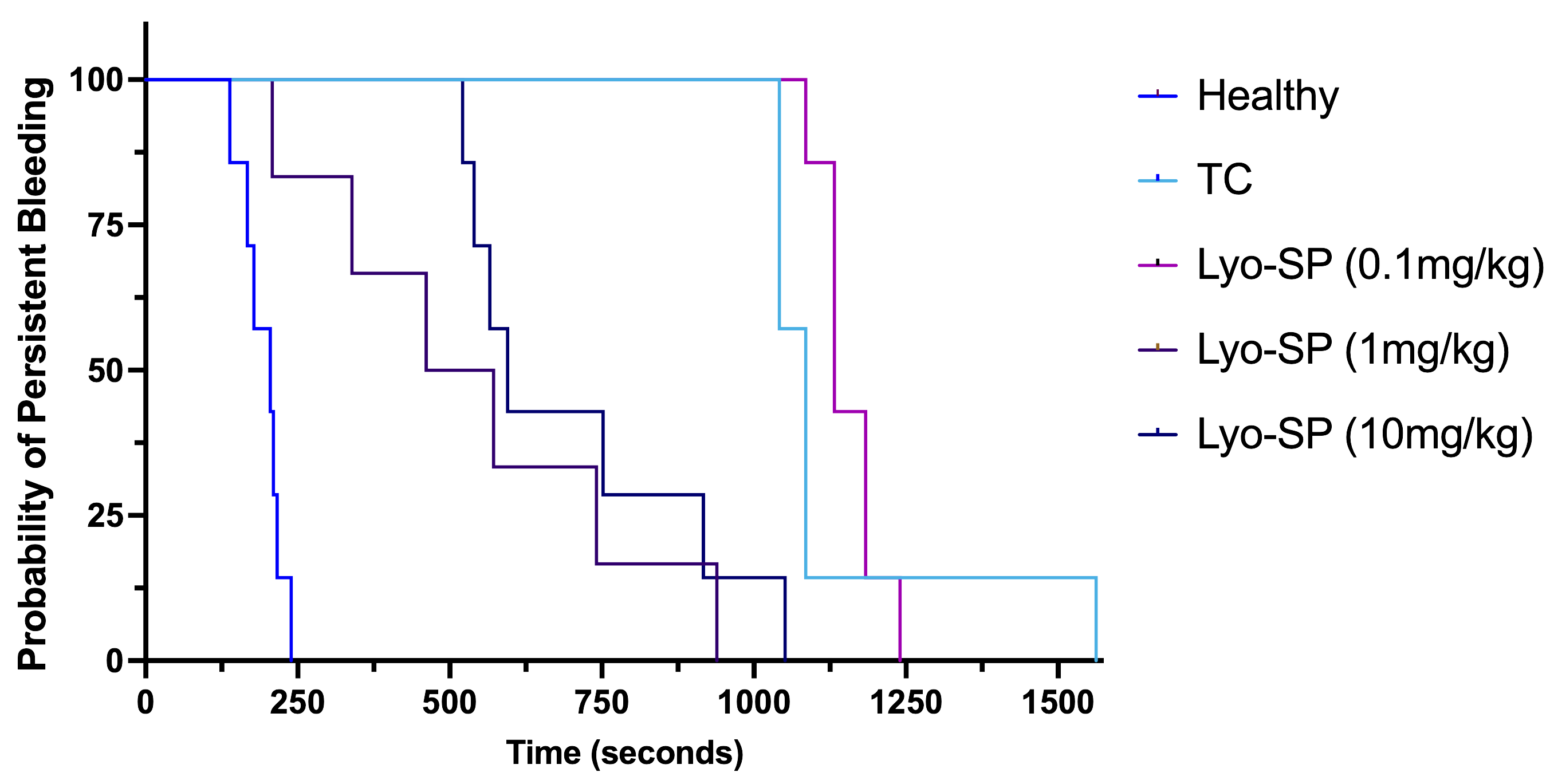


**Figure S18.** Mouse tail-clip bleeding time data shown in Kaplan-Meier format for Lyo-SP treatment of thrombocytopenic (TC) mice, demonstrates that Lyo-SP dose at 1 mg/kg and 10 mg/kg render substantial hemostatic efficacy to reduce tail-bleeding.


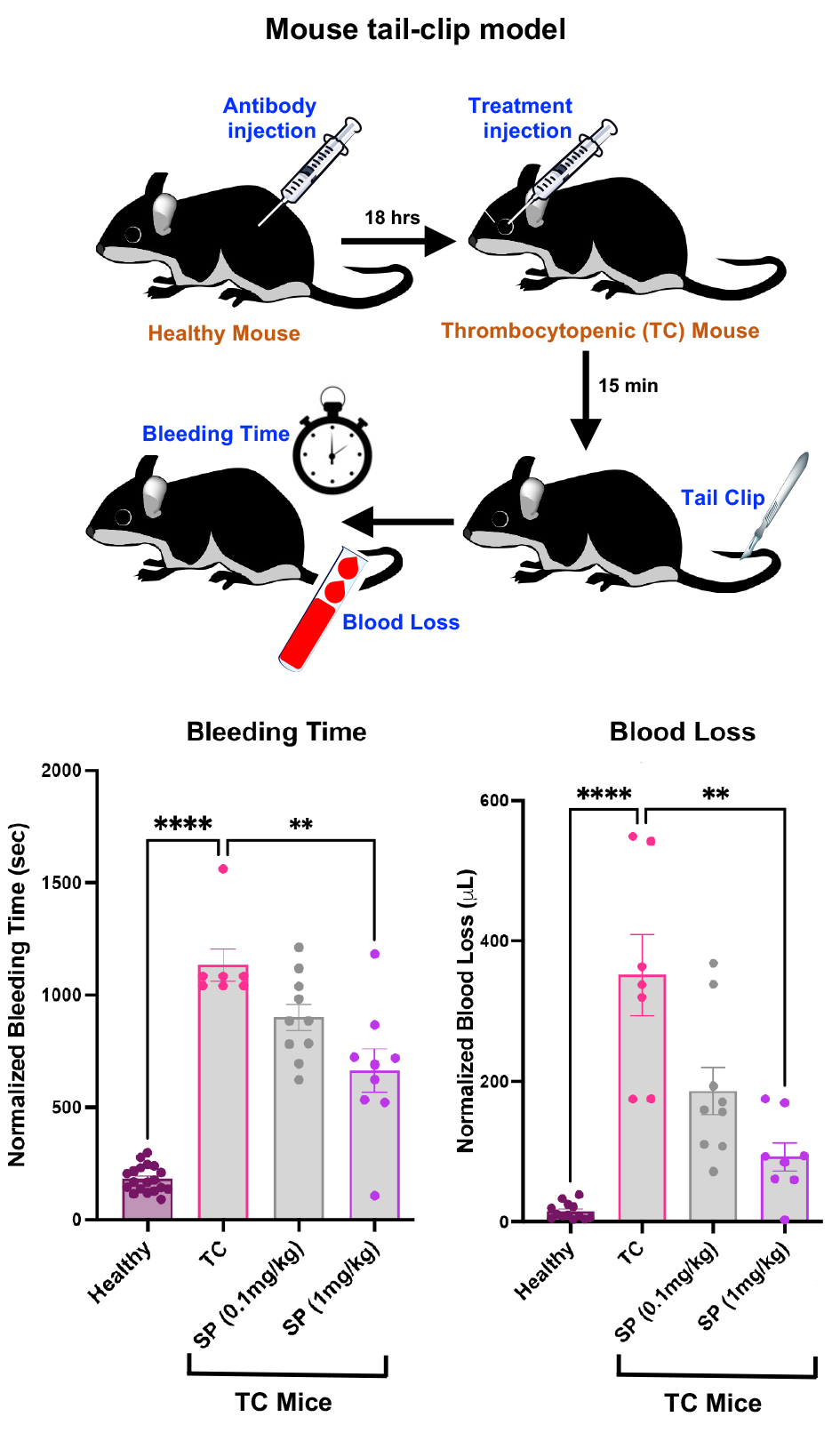


**Figure S19.** Evaluation of Bleeding Time and Blood Loss in tail-clip model in thrombocytopenic mice treated with non-lyophilized synthetic platelet (SP) at doses comparative to Lyo-SP (0.1 mg/kg and 1 mg/kg); SP dose of 1 mg/kg significantly reduced Bleeding Time and Blood Loss in thrombocytopenic (TC) mice at levels comparable to that observed for Lyo-SP-treated TC (shown in Main Figure 7 in the manuscript), confirming that the *in vivo* hemostatic effect of SP remains conserved in Lyo-SP.


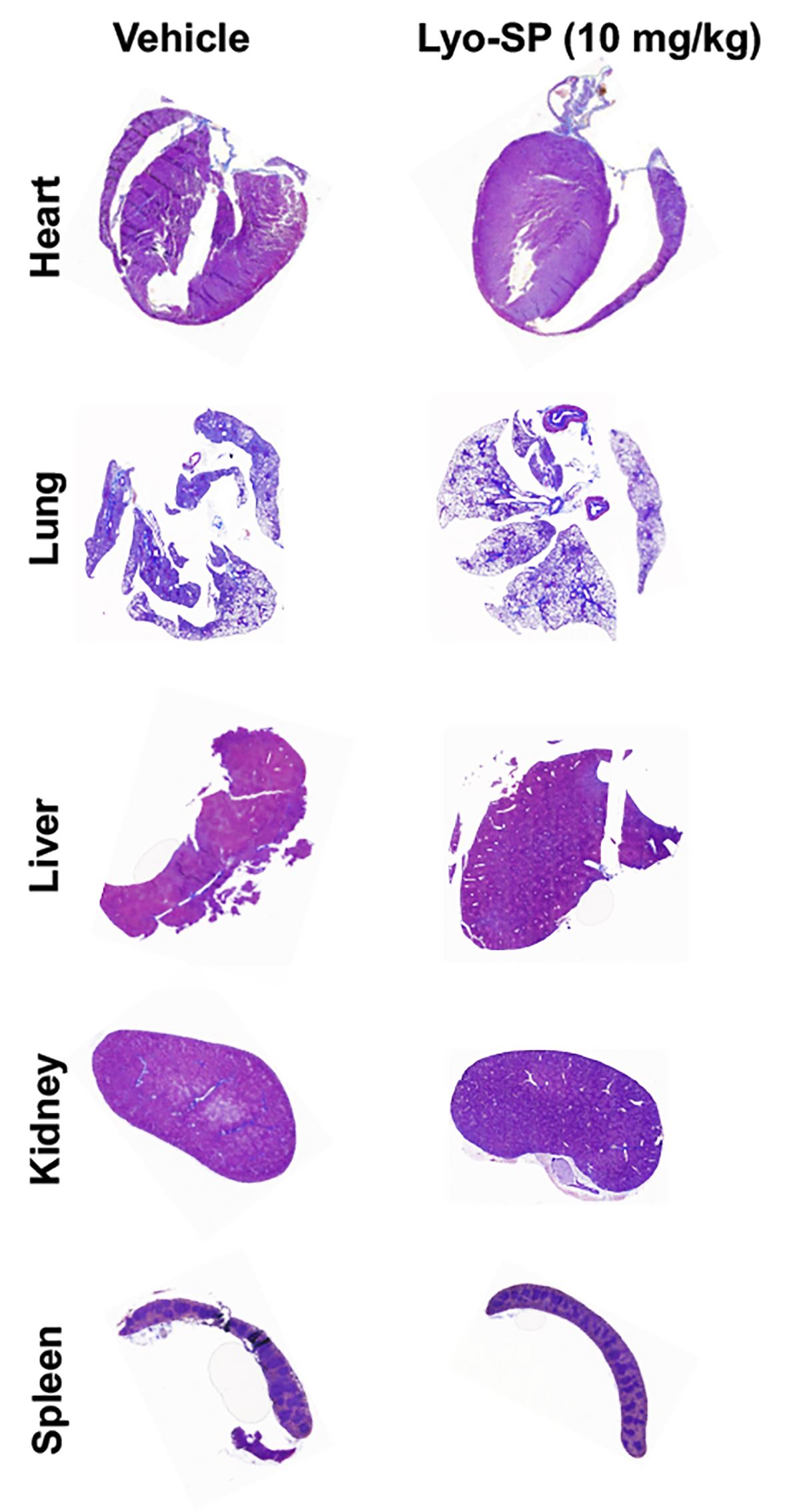


**Figure S20.** Representative histology images (H&E and Carstairs staining) of various clearance organs harvested from mice injected with vehicle compared to that from mice injected with Lyo-SP (n = 5 per group,10 mg/kg dose); No signs of clots (no presence of red-orange fibrin and grey-blue platelets in Carstairs staining) were found in any clearance organ beds for vehicle-dosed or Lyo-SP-dosed mice, suggesting that the therapeutic dose of Lyo-SP has minimal thrombotic risk.

**Movie M1.** Demonstration video of aqueous reconstitution of Lyo-SP.

**Movie M2.** Representative microfluidic imaging video of vWF (cyan) assembly on collagen (indigo blue) coated microfluidic channel at 60 dyn/cm^2^ shear flow.

**Movie M3.** Representative microfluidic video of SP (red) binding to collagen-coated microfluidic surface (collagen not stained) in presence of soluble vWF (cyan) at 60 dyn/cm^2^ shear flow.

**Movie M4.** Representative microfluidic video of Lyo-SP (red) binding to collagen-coated microfluidic surface (collagen not stained) in presence of soluble vWF (cyan) at 60 dyn/cm^2^ shear.

**Movie M5.** Representative microfluidic video of Calcein AM labeled (green) platelets in platelet-rich plasma (PRP) binding to collagen-coated microfluidic surface (collagen not stained) at 60 dyn/cm^2^ shear flow.

**Movie M6.** Representative microfluidic video of Calcein AM labeled (green) platelets in thrombocytopenic plasma (TCP) binding to collagen-coated microfluidic surface (collagen not stained) at 60 dyn/cm^2^ shear flow.

**Movie M7.** Representative microfluidic video of Calcein AM labeled (green) platelets in thrombocytopenic plasma (TCP) treated with red fluorescent SP binding to collagen-coated microfluidic surface (collagen not stained) at 60 dyn/cm^2^ shear flow.

**Movie M8.** Representative microfluidic video of Calcein AM labeled (green) platelets in thrombocytopenic plasma (TCP) treated with red fluorescent Lyo-SP binding to collagen-coated microfluidic surface (collagen not stained) at 60 dyn/cm^2^ shear flow.

**Movie M9.** Representative microfluidic video of Calcein AM labeled (green) platelets in healthy whole blood (WB) binding to collagen-coated microfluidic surface at 60 dyn/cm^2^ shear flow.

**Movie M10.** Representative microfluidic video of Calcein AM labeled (green) platelets in thrombocytopenic whole blood (TC-WB) binding to collagen-coated microfluidic surface (collagen not stained) at 60 dyn/cm^2^ shear flow.

**Movie M11.** Representative microfluidic video of Calcein AM labeled (green) platelets in thrombocytopenic whole blood (TC-WB) treated with red fluorescent Lyo-SP binding to collagen-coated microfluidic surface (collagen not stained) at 60 dyn/cm^2^ shear flow.
